# Identification of Anti-*Mycobacterium* and Anti-*Legionella* Compounds With Potential Distinctive Structural Scaffolds From an HD-PBL Using Phenotypic Screens in Amoebae Host Models

**DOI:** 10.3389/fmicb.2020.00266

**Published:** 2020-02-21

**Authors:** Nabil Hanna, Sébastien Kicka, Gianpaolo Chiriano, Christopher Harrison, Hajer Ouertatani Sakouhi, Valentin Trofimov, Agata Kranjc, Jahn Nitschke, Marco Pagni, Pierre Cosson, Hubert Hilbi, Leonardo Scapozza, Thierry Soldati

**Affiliations:** ^1^Department of Biochemistry, Faculty of Sciences, University of Geneva, Geneva, Switzerland; ^2^Pharmaceutical Biochemistry/Chemistry, School of Pharmaceutical Sciences, University of Geneva, Geneva, Switzerland; ^3^Max von Pettenkofer Institute, Ludwig Maximilian University of Munich, Munich, Germany; ^4^Department of Cell Physiology and Metabolism, Faculty of Medicine, University of Geneva, Geneva, Switzerland; ^5^Swiss Institute of Bioinformatics, Lausanne, Switzerland; ^6^Institute of Medical Microbiology, University of Zurich, Zurich, Switzerland

**Keywords:** *Mycobacterium*, *Legionella*, anti-infective, ChemGPS, amoebae

## Abstract

Tubercular *Mycobacteria* and *Legionella pneumophila* are the causative agents of potentially fatal respiratory diseases due to their intrinsic pathogenesis but also due to the emergence of antibiotic resistance that limits treatment options. The aim of our study was to explore the antimicrobial activity of a small ligand-based chemical library of 1255 structurally diverse compounds. These compounds were screened in a combination of three assays, two monitoring the intracellular growth of the pathogenic bacteria, *Mycobacterium marinum* and *L. pneumophila*, and one assessing virulence of *M. marinum*. We set up these assays using two amoeba strains, the genetically tractable social amoeba *Dictyostelium discoideum* and the free-living amoeba *Acanthamoeba castellanii*. In summary, 64 (5.1%) compounds showed anti-infective/anti-virulence activity in at least one of the three assays. The intracellular assays hit rate varied between 1.7% (*n* = 22) for *M. marinum* and 2.8% (*n* = 35) for *L. pneumophila* with seven compounds in common for both pathogens. In parallel, 1.2% (*n* = 15) of the tested compounds were able to restore *D. discoideum* growth in the presence of *M. marinum* spiked in a lawn of food bacteria. We also validated the generality of the hits identified in the *A. castellanii*–*M. marinum* anti-infective screen using the *D. discoideum*–*M. marinum* host–pathogen model. The characterization of anti-infective and antibacterial hits in the latter infection model revealed compounds able to reduce intracellular growth more than 50% at 30 μM. Moreover, the chemical space and physico-chemical properties of the anti-*M. marinum* hits were compared to standard and candidate *Mycobacterium tuberculosis* (Mtb) drugs using ChemGPS-NP. A principle component analysis identified separate clusters for anti-*M. marinum* and anti-*L. pneumophila* hits unveiling the potentially new physico-chemical properties of these hits compared to standard and candidate *M. tuberculosis* drugs. Our studies underscore the relevance of using a combination of low-cost and low-complexity assays with full 3R compliance in concert with a rationalized focused library of compounds to identify new chemical scaffolds and to dissect some of their properties prior to taking further steps toward compound development.

## Introduction

The emergence of frequent antibiotic-resistant bacteria is reaching a critical point. Classical strategies to identify new antibiotics based on their inhibitory effect on *in vitro* bacterial growth were successful during the 50–60s to identify the main antibiotic classes used today, but they are now reaching their limits. The vast majority of promising chemical scaffolds identified *in vitro* and/or against validated molecular targets failed to show anti-infective activity in infected cells or organisms. This is due largely to unfavorable pharmacokinetic properties or toxicity problems that are revealed at later stages during animal testing or clinical trials. It is necessary today to design new screening procedures, as well as new chemical libraries (Pethe et al., 2010). In addition, the development of new curative treatments against pathogenic bacteria coupled to rationalized political choices constitutes a major challenge for the future of public health (Carlet et al., 2014; Perez et al., 2015).

Over the years, millions of compounds have been synthesized or extracted from natural sources worldwide and are now available for biological screens (Farnsworth et al., 1985; Diop et al., 2018). In addition, the general concept behind the re-screening or repurposing of compounds with new assay systems is that small molecules have an intrinsic ability to interact with different targets with different potency and that an identified chemical scaffold can be developed for a new indication. At the same time, new phenotypic screening methodologies have been established, allowing the detailed study of small molecules interfering with host–pathogen interactions (Wambaugh et al., 2017). These types of assays are amenable to low or medium throughput screens. Taking into account the availability of compounds and the existence of new assays, two strategies can potentially be followed. The first one is based on random screening of millions of compounds, while the second one is based on screening a representative selection enriched for potential hits by using a virtual screening approach (Westermaier et al., 2015). Random, high throughput screening (HTS) campaigns yield a hit rate of ∼1% and are expensive. Screening a selected database yields similar hit rates at a lower cost, with a maximized chemical backbone diversity, and allows the use of low to medium throughput screening systems (Macarron et al., 2011). Indeed, for the design of such small, highly diverse libraries, chemical information scientists have identified unique scaffolds by analyzing the chemical diversity of all the available compounds. Furthermore, microbiology provides information on the pathways and their ligands involved in host–pathogen interactions that allow enriching the highly diverse library with compounds possessing a pharmacophore known to interact with targets of these pathways (Loregian and Palu, 2013).

In drug discovery projects, the chemical entities that are prioritized for biological assessment may encompass a large chemodiversity. In order to visualize the various physico-chemical properties of these compounds, various descriptors such as molecular mass, lipophilicity, and topological features can be computed. They are used to define a multi-dimensional descriptor space known as “Chemical space” (Saldivar-Gonzalez et al., 2019). ChemGPS-NP is an eight-dimensional standardized space that can contribute to compound selection and prioritization notably by cluster analysis and neighborhood mapping (Rosen et al., 2009). Previous studies used the chemical space navigation tool ChemGPS-NP to compare 60 anti-mycobacteria-active natural products and 39 anti-Mtb drugs and drug candidates with respect to physico-chemical properties and their occupation of chemical space. In physico-chemical space, both sets largely overlapped and defined a sub-region of Chem-GPS-NP space (Liu et al., 2016).

In recent years, several phenotypic screens performed in the context of infected animal host cells have identified compounds active against pathogenic mycobacteria and *Legionella pneumophila* (Escoll et al., 2013; Foo et al., 2018; Machelart et al., 2019). Interestingly, elucidation of the mode of action of hits revealed that intracellular bacteria proliferation can be affected by targeting bacterial metabolic pathways key to their intracellular life, but also by modulating host pathways (Sundaramurthy et al., 2014; Delorme et al., 2015; VanderVen et al., 2015). Alternative screening systems have also been established to take advantage of previously underexplored cellular amoeba host models (Harrison et al., 2013, 2015a,b; Kicka et al., 2014). Free-living amoebae (FLA), naturally present in soil and water, predate on bacteria and fungi that they ingest by phagocytosis (Cosson and Soldati, 2008; Scheid, 2014). Indeed, amoebae share ecological niches with most bacteria, and are putative cellular reservoirs for pathogenic bacteria (Greub et al., 2004; Hilbi et al., 2007). In addition, FLA have been described as “trojan horses” for many pathogens including *Legionella* and *mycobacteria* species (Molmeret et al., 2005). Due to the extreme conservation of phagosomal composition and function with human phagocytic cells (Boulais et al., 2010), *Dictyostelium discoideum* is used as a host cell to investigate interactions with pathogenic bacteria such as *Salmonella*, *Mycobacteria*, *Legionella*, or *Pseudomonas* (Solomon et al., 2003; Steinert and Heuner, 2005; Hagedorn and Soldati, 2007; Dunn et al., 2018). It has also been used to identify anti-virulence compounds against *Pseudomonas aeruginosa* (Bravo-Toncio et al., 2016).

At the cellular level, *L. pneumophila* and *Mycobacterium marinum* subvert cellular compartments and machineries to establish a permissive replication niche. After their uptake by the host cell, each pathogen develops specific strategies. *M. marinum*, like *Mycobacterium tuberculosis* (Mtb), induces phagosome maturation arrest and restricts its acidification. Then, the phagosome becomes an active interface between the host cell and the mycobacteria, by interacting with host machineries such as autophagy, endosomal, and other compartments, and finally loses its integrity, giving *M. marinum* access to the host cytosol (Cardenal-Munoz et al., 2017, 2018; Lopez-Jimenez et al., 2018). On the other hand, *L. pneumophila* establishes a structurally unique spacious vacuole, the LCV, that intimately interacts with the host endoplasmic-reticulum compartment (Escoll et al., 2013; Prashar and Terebiznik, 2015; Steiner et al., 2018; Swart et al., 2018). To establish the LCV, *Legionella* deeply modifies the host vesicular traffic by delivering, via a type-IV secretion system, a panel of effectors interacting with host components (Finsel and Hilbi, 2015; Simon and Hilbi, 2015). In addition, the fates of each pathogen are also different. *L. pneumophila* resides and proliferates in the LCV and bacteria are possibly released by host cell lysis, while *M. marinum* escapes to the cytosol and finally egresses the host cell using various routes including a specific structure named “ejectosome” (Hagedorn et al., 2009). In the present study, we focused on two pathogenic bacteria that currently represent a human threat: *L. pneumophila*, the causative agent of a severe pneumonia known as legionellosis or Legionnaires’ disease (Cunha et al., 2015), and *M. marinum*, a close relative to Mtb that causes tuberculosis, a major health burden in human populations (Dheda et al., 2016). Both mycobacteria are considered facultative intracellular species because of their capacity to grow within the host cell and also in the extracellular space or the environment.

Since the primary target of Mtb is macrophages, amoebae that are also professional phagocytes, are a rational choice to study host–pathogen interactions. For example, *D. discoideum* and *Acanthamoeba castellanii* were used to characterize compounds of the GlaxoSmithKline (GSK) TB-set of anti-mycobacterial compounds (Trofimov et al., 2018). This study showed that most compounds previously selected for their antibiotic activity against *Mtb* and *Mycobacterium smegmatis* (Ballell et al., 2013) were active against the closely related *M. marinum*, but showed little or no activity in the intracellular context of an infection. Most importantly, it demonstrated that compounds with anti-infective activities were similarly active in the *M. marinum*–amoebae system and the more standard *M. marinum*–macrophage model. This study also underlined the relevance of using evolutionary distant pathogen and host models to reveal conserved mechanisms of virulence and defense (Trofimov et al., 2018).

Here, we tested compounds derived from a unique ligand-based virtual screen to determine their anti-infective properties in the two infection models *A. castellanii*–*L. pneumophila* (referred to as “AcLp screen”) and *A. castellanii–M. marinum* (referred to as “AcMm screen”) for their abilities to inhibit intracellular growth. In parallel, the “anti-virulence” activity of the compounds was determined by monitoring their capacity at reverting the growth arrest of *D. discoideum* on lawns of food bacteria spiked with pathogenic *M. marinum* (referred to as “DdMm screen”). Finally, the potency, specificity, and toxicity of the hits were evaluated. In addition, we conducted further analyses to evaluate the drug-like properties and chemical space of the anti-mycobacterial and anti-*Legionella* hit compounds using ChemGPS-NP.

## Results

### Design of a Chemically Highly Diverse Pathway-Based Library of Compounds

*Legionella* and *Mycobacteria* interact with a host through different pathways, which can be searched for potential drug targets. Instead of focusing on a single target for a drug design, we have explored the pathways involved in the host–pathogen interaction process, and not only those having a significant pathogen–host selectivity ratio. We selected in total 18 host and pathogen pathways as potential pharmacological targets. Ligands/metabolites known to interfere/interact with these host and pathogen pathways were identified from the available literature, and used as queries or search templates to prepare pharmacophores for launching a campaign of ligand-based virtual screening (LVS) of the ZINC database^1^ (Sterling and Irwin, 2015) using ROCS, a tool from the OpenEye software package^2^ (Swann et al., 2011).

**FIGURE 1:**
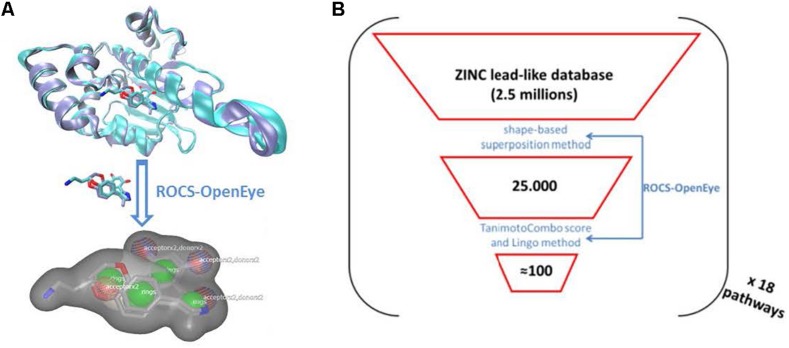
**(A)** Schematic representation of the LVS workflow applied per each pathway. **(B)** A visualization of the Ligand-based virtual screen of the ZINC lead-like database; (i) ranking of the 25,000 best virtually screened hits according to the ROCS TanimotoCombo score; (ii) selection of the first hit and then each next one if structurally dissimilar to already chosen ones by using the Lingo method; (iii) we chose at most two analogs per series from each screened pathway saving 100 selected compounds to the pool of potential hits composing the physical library for the experimental screen.

Subsequently, we applied the following workflow (Figure 1): (i) we screened the ZINC lead-like database composed of 2.5 million compounds saving the 25,000 best hits for each query; (ii) we ranked the hits according to the ROCS TanimotoCombo score; (iii) we selected the first hit and the following, if they passed a test of structural dissimilarity, using the Lingo method program (Vidal et al., 2005), thereby increasing the chemical diversity and maximizing the coverage of the chemical space of the ZINC lead-like database; (iv) we chose at most two analogs per series from each screened pathway, saving 100 selected compounds to the pool of potential hits composing the physical library for the experimental screen. The VS of the 18 different pathways yielded ∼1800 compounds of which 1255 were purchased to compose the final Highly Diverse Pathways-Based Library; we named HD-PBL (Westermaier et al., 2015; Slepikas et al., 2016).

### Characterization of the Pathways-Based Library Properties: Chemical Diversity and Drug Likeness

**FIGURE 2:**
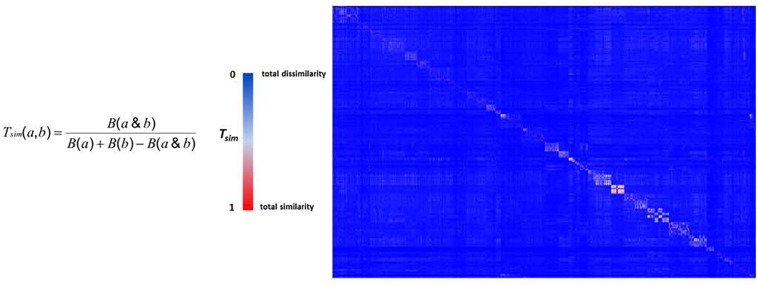
The heat map of pathways-based library. The Z-matrix was calculated according to T_sim_ values: T_sim_ = 0 for total dissimilarity (blue); T_sim_ = 1 for total similarity (red).

**FIGURE 3:**
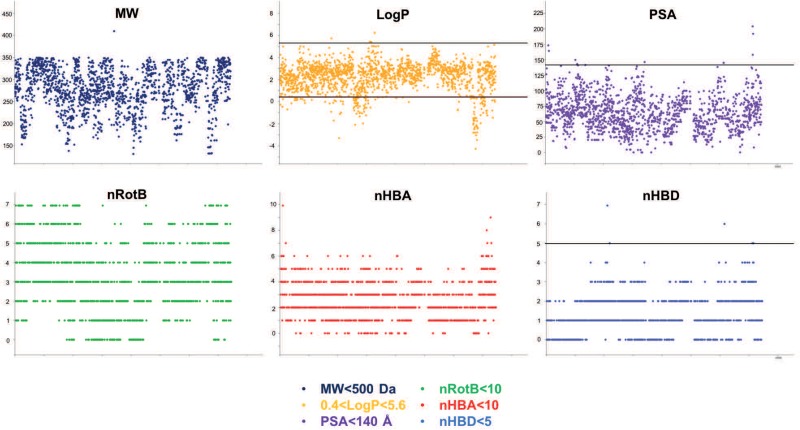
Physico-chemical prediction of the HD-PBL. The prediction descriptors of the library were performed using the cheminformatics package Canvas to evaluate the drug-likeness properties of library’s compounds (Ghose et al., 1999). MW: molecular weight, logP: partition coefficient, PSA: polar surface area, nRotB: number of rotatable bonds, nHBA: number of hydrogen bond acceptors, nHBD: number of hydrogen bond donors.

The chemical diversity of the pathways-based library was investigated and analyzed on the basis of the Z-matrix calculated according to Tanimoto’s chemical similarity metrics (*T*_sim_; see Figure 2) using Canvas, a tool from the Schrödinger software package (Sindhikara and Borrelli, 2018). The corresponding heat map drawn using Netwalker1.0 (Komurov et al., 2012) clearly shows that the library is highly diverse (Figure 2). We deliberately limited the number of analogs in the primary library to concentrate on the validated hits selected from the different screens. The analysis of the drug likeness of the compounds composing the library and the comparison with the known drug-likeness standards (Lipinski et al., 1997; Lipinski and Hopkins, 2004; Lipinski, 2016) reveals that the vast majority of the compounds have the properties to be drug-like (Figure 3).

### Screens Characteristics

The 1255 HD-PBL compounds were assayed using three biological phenotypic screens (see section “Materials and Methods”). The categories of pharmacophore queries are indicated in Table 1, and the hallmarks of the various screens are summarized in Table 2.

**TABLE 1:** Host and pathogen metabolic pathways used for selecting the query molecules.

Cellular pathways and targets	Query pharmacophores	References
Calcineurin 1	Pyrazolopyrimidines, CN585	Sieber and Baumgrass, 2009; Jayaraman et al., 2010
E3-Ligase	HLI98	Yang et al., 2005
AKT-PKB	Indoles, pyridopyrimidine, pyrazolopyrimidine, pyranonapthoquinones, quinolines, azacarabazoles, oxazole-2-amines	Tardy et al., 2014
PI3K	Wortmanin, LY294002, staurosporin, ATP, myricetin, quercetin	Walker et al., 2000
Autophagy	Small molecule enhancers of rapamycin = SMERs: SMER18 and 28 from, 2007. Diphenylbutylpiperidines—cpds 15d, 15i, 15j	Floto et al., 2007; Sarkar et al., 2007; Chen et al., 2011
ATPase	Salicylihalamide A, indole derivatives H^+^-ATPase; diaryl pyrazole resorcinol derivatives HSP90 ATPase; adociasulfate-2 kinesin ATPase	Sakowicz et al., 1998; Sharp et al., 2007; Supino et al., 2008; Design, 2009
CD36	Cilostazol, sulfo-*N*-succinimidyl derivatives	Coort et al., 2002
ABC transporters	Verapamil, LY335979, OC144-093	Sharom, 2008
Trp	CdRP + IGP; ATB107 IGPS target	Hennig et al., 2002; Shen et al., 2009
Cys	WNI tripeptide; 3-hydroxybutanoic acid, 5-oxohexanoic acid, 4-methyl valeric acid—*O*-acetyl serine synthethase target	Salsi et al., 2010
His	PRPP; PR-ATP; cpds 4 6 17 18; dicoumerol ATP-PRT target	Dall-Larsen et al., 1976; Cho et al., 2008
Gln	Purine analog; glutamine synthetase target	Nilsson et al., 2009
Lipase	Orlistat, glimepiride	Bertrand et al., 2010; Bustanji et al., 2011; Christelle et al., 2011; Pedicord et al., 2011; Schalk-Hihi et al., 2011
FAS-II	Triclosan, triclocarbon	Lu et al., 2011
Cations pumps	Acetazolamide, zonisamide, topiramate, phenytoin, lamatrigine, sumatriptan, verapamil	Long et al., 2005; Wu et al., 2010; Payandeh et al., 2011
Proteasome	Lactacystin, omuralid, MG-132, morphine	Yang et al., 2008; Yoshida et al., 2009
MeC	Spongotine A, bromotopsentin, bromodeoxytopsentin, *cis*-3,4-dihydrohamacanthin B	Zoraghi et al., 2011
Porins	Cadaverine, putrescine, spermine	Iyer et al., 2000

*The corresponding pharmacophores of the queries molecules have been used as reference molecules for the ligand-based virtual screen. Each pathway-based screen resulted in 100 new potential hits.*

**TABLE 2:** Screen assays characteristics.

A	Anti-infective screens		Anti-virulence screen
Host	*A. castellanii* and *D. discoideum*	*A. castellanii*	*D. discoideum*
Pathogen	*M. marinum*	*L. pneumophila*	*M. marinum* (mixed 1:50 with *K. pneumoniae*)
Duration	2–3 days	2 days	At least 1 week
Read-out (type)	(Quantitative) intracellular bacterial growth (GFP reporter)		Host growth on mixed bacterial lawns (semi-quantitative)
Compound concentration	30 μM (solution)		10 μM (agar)

**B**	**Hit categories**		

Anti-infective	Yes	Yes	Possibly^a)^
Anti-virulence	Yes	Yes	Yes
Pro-infective	Yes	Yes	No
Antibiotic	Yes^b)^	Yes^b)^	Yes (mycobacteria-specific)
Defense booster	Yes	Yes	Yes

***^*a)*^** Further investigations are needed to disentangle whether the activity of the compound is affecting the extracellular or the intracellular bacteria. **^*b)*^** The anti-infective screen on *M. marinum* detected mainly hits with low antibiotic activity, whereas the screen on *L. pneumophila* detected mainly potent antibiotics.*

**FIGURE 4:**
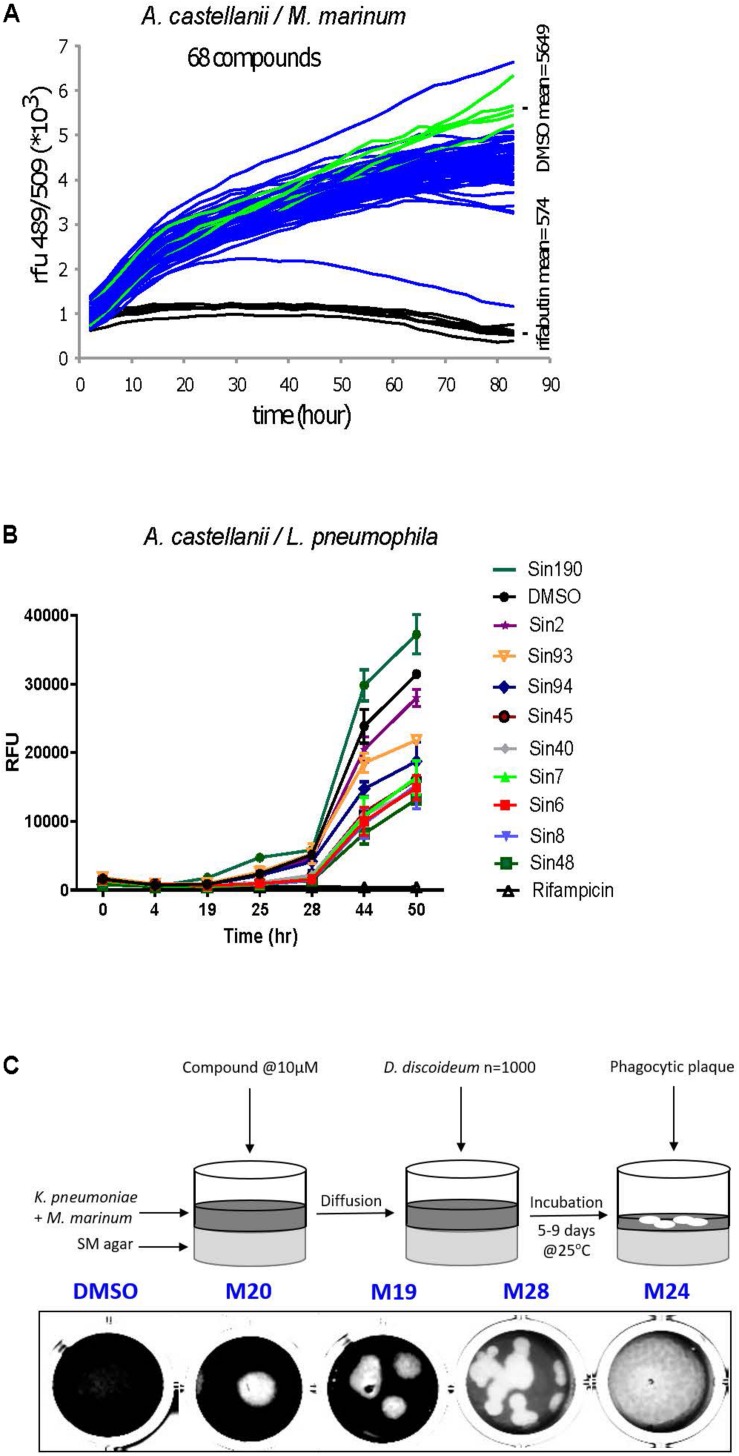
A representative selection of the HD-PBL compounds tested in the three different assays. Phagocytic host cell *A castellanii* was infected with **(A)** *M. marinum* msp12:GFP and **(B)** GFP-expressing *L. pneumophila*, 5 × 10^4^ infected cells were transferred to each well of a 96-well plate. The course of infection at 25°C was monitored by measuring fluorescence for 72 h in the presence of 30 μM of corresponding compounds compared to DMSO control (green) and 10 μM rifabutin—only for *M. marinum* assay (black). **(C)** Virulence assay. Each compound (10 μM) was added on SM-agar medium followed by the addition of *K. pneumoniae* and *M. marinum* mixture; 1000 *D. discoideum* cells were deposited in the center of the well and plates were incubated for 5–9 days at 25°C and the formation of phagocytic plaques was monitored visually.

Each of the three assays was established and optimized separately prior to testing the 1255 compounds (Froquet et al., 2009; Harrison et al., 2013; Kicka et al., 2014). The three screens were run simultaneously using the same compounds batch. The compounds’ concentration was adjusted to 30 μM for the two cell infection screening assays, taking into account the shielding effect of *A. castellanii* cells on intracellular bacteria (Tosetti et al., 2014), whereas a final concentration of 10 μM was used in the anti-virulence assay monitoring *D. discoideum* growth on bacteria lawns (Table 2). Considering screen stringency variations, the threshold for hit detection was arbitrarily fixed at a minimum of 20% reduction of the intracellular growth of *M. marinum*, and 40% reduction for intracellular growth of *L. pneumophila* compared to the DMSO control. Fluorescence from GFP-producing bacteria was used as a readout of intracellular growth for both anti-infective assays in the *A. castellanii* host (Figure 4). Briefly, *A. castellanii* cells were infected with GFP-producing *M. marinum* or *L. pneumophila*. Intracellular bacteria growth was monitored by measuring the fluorescence increase using a plate reader with time-points taken every 3 h for *M. marinum*, and at various time intervals for *L. pneumophila* as indicated in Figure 4. Extracellular *M. marinum* growth was inhibited by adding 10 μM amikacin to the medium, whereas no antibiotic was added to *L. pneumophila* since the PYG medium used for infections does not support extracellular bacterial growth. Examples of the intracellular bacterial growth curves are presented in Figures 4A,B for *M. marinum* and *L. pneumophila*, respectively. Normalization and analysis of the data generated in these two screens were performed as previously reported for the AcMm screen (Kicka et al., 2014; Ouertatani-Sakouhi et al., 2017) and the AcLp screen (Harrison et al., 2013) host–pathogen assays, respectively. In brief, normalization of bacterial growth in the presence of each compound was calculated related to the DMSO carrier (=1).

For the plaque assay, a semi-quantitative visual inspection and scoring of the compounds was applied, as described previously (Froquet et al., 2009). As shown in Figure 4C, the ability of hits to restore *D. discoideum* growth was quantified to range from inert molecules (=0) to compounds that fully restore host growth (=4). Following their identification in their respective initial screen, candidate hits were independently validated at least three times in their respective assay.

### Hit Frequency and Overlap Between Screens

**FIGURE 5:**
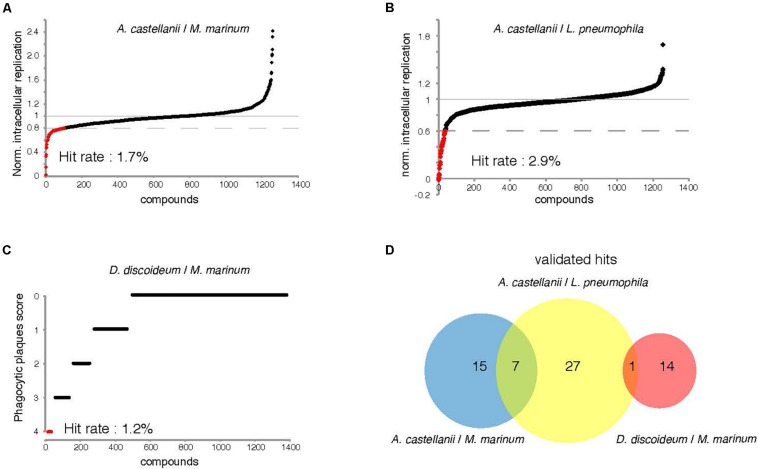
Percentage hit rate **(A–C)** and hits overlap of the three screens. All 1255 compounds were tested for potential intracellular growth inhibition. Library compounds were plotted based on their anti-infective properties. Compounds resulting in decreased intracellular *M. marinum* and *L. pneumophila* replication by over 20 and 40%, respectively, at the screening concentration (30 μM) were defined as hit candidates. **(C)** Compounds score from the “phagocytic plaque assay.” The potency of each compound to restore *D. discoideum* growth was evaluated, hits were determined as molecules that fully restore host growth (=4). **(D)** Venn diagram representing summarized results of the primary hits identified from the three assays. The analyzed set included compounds that passed the aforementioned cut-offs.

The two intracellular growth assays used for screening all 1255 compounds resulted in a broad range of inhibitory activities. The hit rate was considerably higher for *L. pneumophila*, where 2.8% (*n* = 35) of the compounds tested at 30 μM showed a growth inhibition of at least 40%, compared to 1.7% (*n* = 22) withat least 20% inhibition for *M. marinum* (Figures 5A,B). The DdMm plaque assay screen showed that 1.2% (*n* = 15) of the compounds at 10 μM are valuable hits restoring amoeba growth (Figure 5C). In comparison to infection assays, full restoration of amoeba growth on a bacteria lawn containing pathogenic mycobacteria appeared to be more restrictive and identified only 15 hits from the library. Taken together, 64 compounds showed anti-infective activity in one of the intracellular growth assays or attenuated mycobacterial virulence, respectively. As depicted in the Venn diagram, the AcLp screen shared eight hits with the other two screens, and among these, seven are common for the two infection assays and only one for the *L. pneumophila*–*A castellanii* test and the phagocytic plaque assay (Figure 5D). Surprisingly, no hit was common to the two assays using *M. marinum* as pathogenic bacterium. Notably, we also identified a certain number of pro-infective compounds, namely, chemicals that lead to a significant increase of the intracellular bacterial numbers compared to the DMSO control. Five pro-infective compounds were identified with at least twofold increase in the intracellular *M. marinum* bacterial load (Figure 5A). In contrast, the AcLp assay identified only one compound that increased the intracellular growth more than 40% when compared to the DMSO control (Figure 5B).

### Effect of Hit Compounds on Amoeba Fitness

**FIGURE 6:**
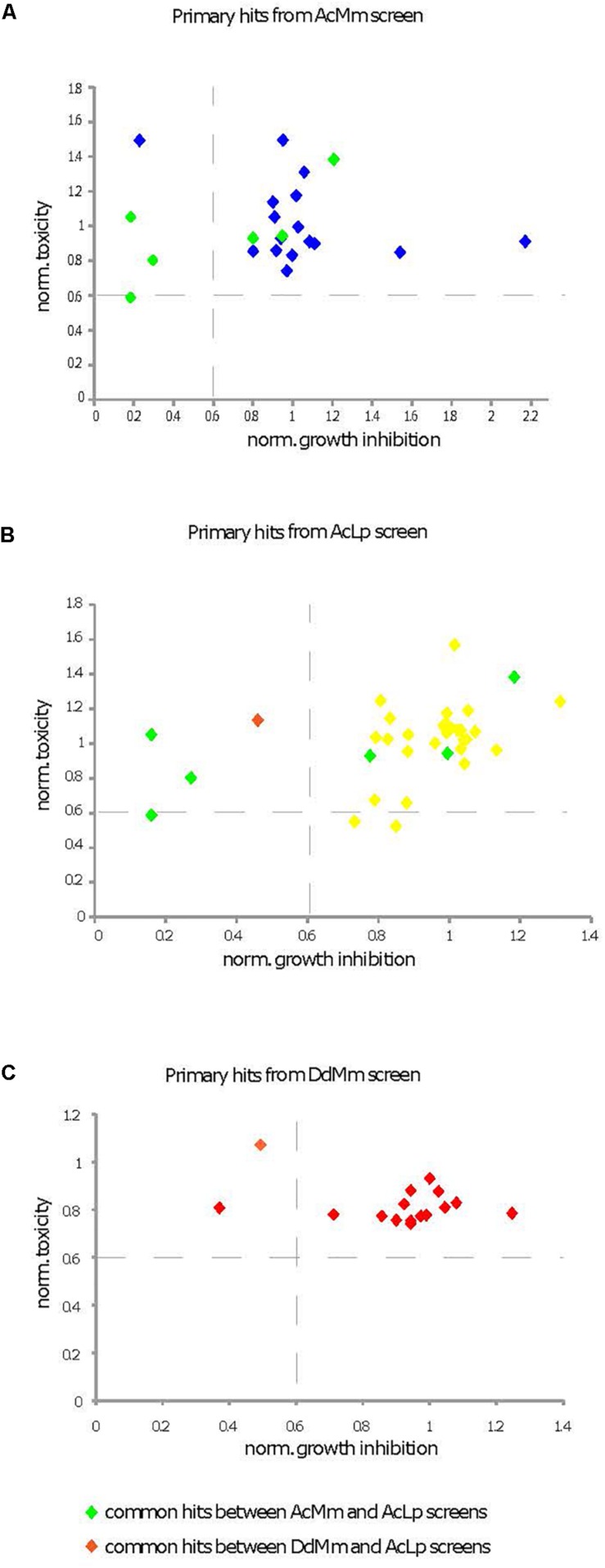
Characterization of cytotoxic and growth inhibitory activities of primary hits. Primary hits come from the three different screens [**(A)** AcMm refers to *A. castellanii–M. marinum*, **(B)** AcLp refers to *A. castellanii–L. pneumophila*, and **(C)** DdMm refers to *D. discoideum–M. marinum*]. Two different assays were compared, a cytotoxicity test in *A. castellanii* and a growth inhibition assay in *D. discoideum*. Compound cytotoxicity (*y*-axis in A–C) against *A. castellanii* was determined using the Alamar blue reagent. The corresponding data are presented in Supplementary Table S2 column K. The growth inhibitory activity of compounds on *D. discoideum* GFP-ABD was measured with a fluorescence plate reader (*x*-axis in A–C). The corresponding data are presented in Supplementary Table S2 column I. The compounds were tested at 30 mM and values were normalized to the DMSO carrier control (=1).

To evaluate the effect of hit compounds (referred to as “hits”) on host fitness, we used *D. discoideum* cells expressing a GFP construct to measure toxicity and growth inhibitory activities of compounds (Trofimov et al., 2018). In parallel, a cell viability test using Alamar blue was performed using *A. castellanii* 4 h after hit compound addition at a 30 μM concentration. For each assay, values were normalized to the DMSO carrier control (= 1). The compounds’ toxicity and growth inhibition data for each phenotypic screen are represented in Figures 6A–C.

Only one compound (ZINC01718072) was excluded from further analysis, because its strong fluorescence at the GFP emission wavelength confounded the results. At the end, five hits did not pass the set threshold (less than 40% growth inhibition or 40% toxicity when compared to DMSO control) and were rejected for this deleterious effect on host fitness.

### Properties of the Hits

**FIGURE 7:**
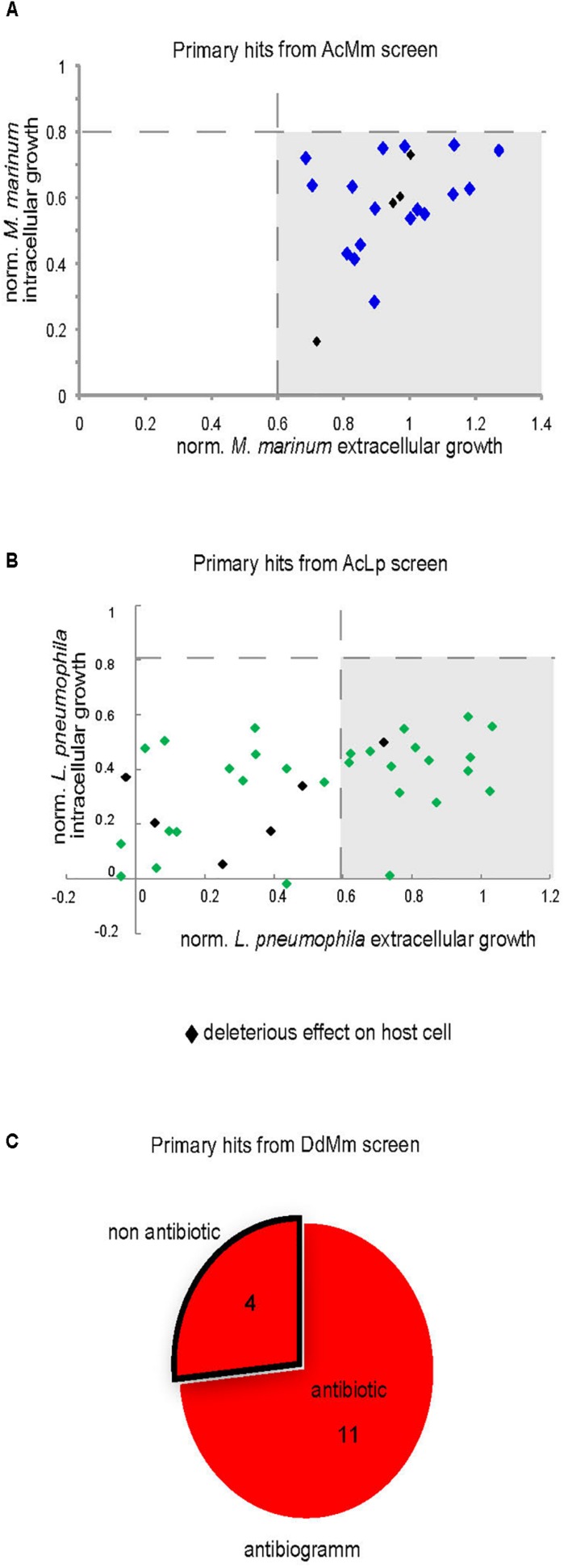
Intracellular and extracellular growth of the pathogenic bacteria in presence of the primary hits. Growth of *M. marinum* **(A)** and *L. pneumophila* **(B)** in the presence of hits (30 μM) was determined for both intracellular (*A. castellanii*) and extracellular replication (broth). The graphs indicate the fluorescence measurements normalized to 1 (vehicle control). **(C)** Antibiotic assay. Compounds (10 μM) identified in phagocytic plaques assay were added to 7H11 medium in each well and then 1000 *M*. *marinum* bacteria were deposited in the well. Growth of mycobacteria was monitored after 6 days at 30°C.

To determine whether hits had antibiotic properties, we measured their activity against *M. marinum* or *L. pneumophila* growing in broth. Compounds at 30 μM in DMSO were transferred to 96-well plates containing 10^5^ GFP-producing *M. marinum* or *L. pneumophila* per well. Growth was monitored for 48 hours, and the total fluorescence intensity was used as a proxy to quantify the bacterial numbers. In parallel, we tested the ability of hits identified by the phagocytic plaque assay to directly inhibit *M. marinum* growth on agar at 10 μM. The normalized results (DMSO = 1) of the hits detected during cell infection assays are shown in Figures 7A,B for *M. marinum* and *L. pneumophila*, respectively. In summary, none of 21 hits exhibited antibiotic properties against *M. marinum*, whereas 18 of the 35 hits against intracellular *L. pneumophila* showed an antibiotic activity. Lastly, 11 compounds out of fifteen from the anti-virulence hits against *M. marinum* exhibited mild to strong antibiotic activity when assayed for bacterial growth inhibition on agar (Figure 7C).

### The *D. discoideum–M. marinum* Host–Pathogen Model System

**FIGURE 8:**
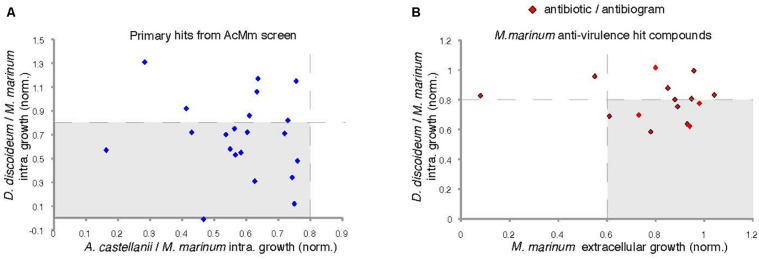
Comparison of two amoeba host models. **(A)** Comparison of anti-infective properties of primary hits on intracellular *M. marinum* growth in *D. discoideum* and *A. castellanii* host cells. **(B)** Selected anti-virulence hits plotted based on their anti-infective (20% inhibition) and anti-bacterial (40%) activity in *D. discoideum–M. marinum* model. 10^5^ *D. discoideum* cells infected with *M. marinum* msp12:GFP were transferred to the wells, hits were added at 30 μM. The fluorescence intensities were measured for 72 h every 3 h.

To validate the generality of the anti-infective screen performed using the AcMm system, we tested the 21 identified hits using the DdMm host–pathogen model. Fourteen compounds out of 21 exhibited at least a 20% inhibitory activity on intracellular mycobacteria (Figure 8A). Interestingly, from the hits identified in the “extracellular” anti-virulence plaque assay, almost 50% (7/15) of the compounds showed a growth inhibitory effect on mycobacteria when tested in the intracellular *D. discoideum* infection model. Curiously, the antibiotic potency of these hits, as detected using growth of bacteria on agar, was barely detectable in the 2-days bacteria growth assay in suspension, and only two compounds showed an inhibition intra- and extracellularly (Figure 8B—dots on the edge of quadrant 1).

### Classification and Clustering of Anti-*M. marinum* Hits, Anti-*L. pneumophila* Hits, and Anti-Mtb Drugs Using CHEM-GPS

**FIGURE 9:**
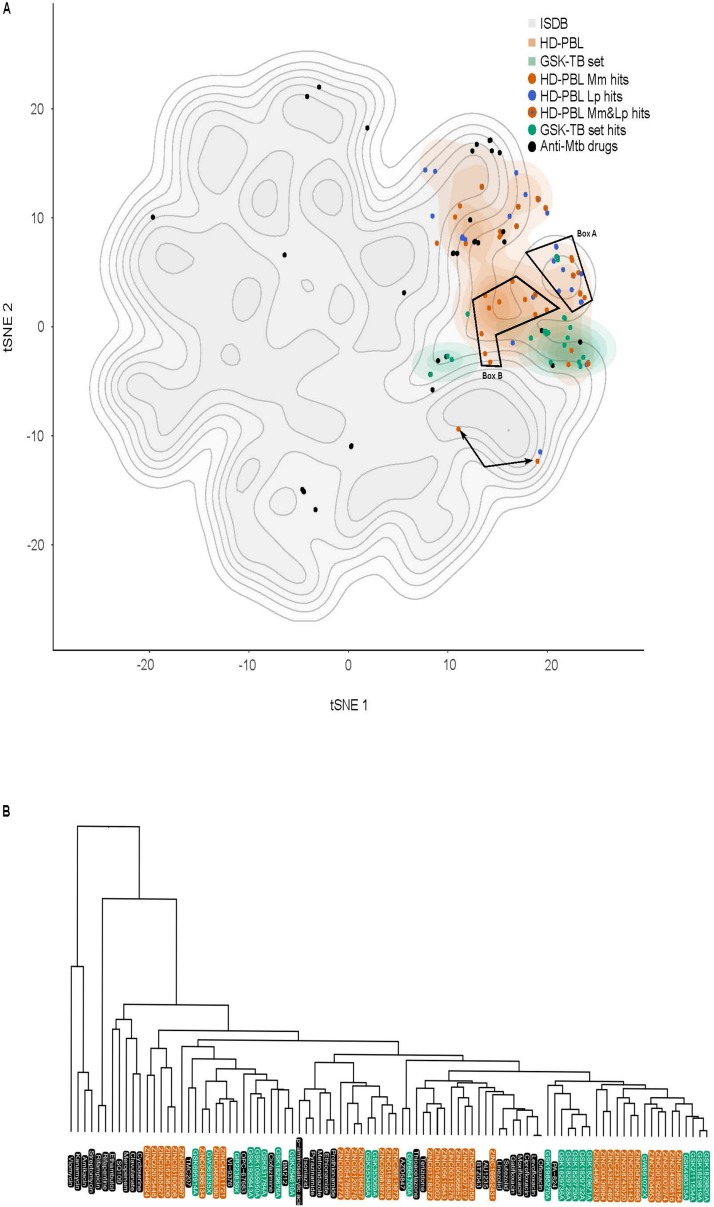
ChemGPS-NP analysis of anti-Mtb hits. **(A)** ChemGPS-NP-based analysis of the chemical space occupied by 211,000 compounds extracted from ISDB (gray background), the standard and candidate anti-Mtb drugs (black dots), the HD-PBL (orange cloud = non-hits, orange dots = anti-*M. marinum* hits, blue dots = anti-*L. pneumophila* hits, orange dots with a blue center = hits active against both *M. marinum* and *L. pneumophila*), and the GSK TB set (green cloud = non-hits, green dots = anti-*M. marinum* hits). To improve visualization, 2D t-distributed stochastic neighbor embedding (tSNE) was applied to the eight output PCs of ChemGPS-NP. **(B)** Hierarchical classification of anti-mycobacterial compounds computed using the Euclidian distance in the Chem-GPS-NP space with average linkage: standard and candidate anti-Mtb drugs (black); GSK TB set hits (green); HD-PBL hits (orange). The dendrogram illustrates the arrangement of the clusters produced by the complete linkage clustering method.

In order to evaluate the distribution of the 1255 compounds and hits from the HD-PBL in the physicochemical space, we used the chemical global positioning system of natural product (ChemGPS-NP; Larsson et al., 2007; Rosen et al., 2009). ChemGPS-NP is a global chemical positioning system that utilizes eight standardized dimensions, similar to principal components (PC) to describe the physico-chemical properties of compounds, and is specifically tuned for the exploration of the biologically relevant chemical space of natural products. We used this tool to compare the compounds described in the present study with known standard and candidate anti-Mtb drugs (including both marketed and promising compounds presently in clinical trials; Dashti et al., 2014), as well as the compounds and hits from the GSK TB set (Trofimov et al., 2018), together with the 211,000 compounds from the *in silico* MS/DS Database (ISDB). The ChemGPS-NP eight-dimensional output was explored by inspecting every pair of dimensions. Eventually, the two-dimensional representation provided by t-distributed stochastic neighbor embedding (tSNE) was utilized to visually summarize the ChemGPS-NP analysis. Figure 9A clearly shows that the drug-like properties of anti-Mtb drugs (black dots) are remarkably scattered on the full ISDB reference cloud, possibly indicating a large variety of mode of actions and targets. The 1255 compounds from the HD-PBL (orange) and the 177 from the GSK TB set (green) formed two separate clouds that occupy a much more restricted subspace compared to the anti-Mtb drugs. The hits from HD-PBL (orange dots—anti-*M. marinum* hits, blue dots—anti-*L. pneumophila* hits, and orange dots with a blue center—hits active against both *M. marinum* and *L. pneumophila*) appeared dispersed inside the cloud of the HD-PBL, and surrounded by standard and candidate anti-Mtb drugs (black dots). However, closer inspection revealed that some HD-PBL hits formed two sub-clusters (box A and B) separated from the other HD-PBL hits. In addition, 3 HD-PBL hits (arrows) stand further apart, indicating a distinct structural scaffold. A hierarchical classification of standards and candidate anti-Mtb drugs (black), together with the hits from the GSK TB set (green), and HD-PBL (orange) was computed using average linkage of Euclidian distances in the original ChemGPS-NP space (Figure 9B). This classification confirmed the structural differences existing between the various standard and candidate anti-Mtb drugs, which also appeared dispersed in the dendrogram. Overall, the hits from the GSK TB set and HD-PBL appear to have diverse structural scaffolds and are distributed along the dendrogram. Interestingly, a mixed group of hits from GSK TB set (*N* = 10) and of HD-PBL (*N* = 12) clustered on the right of the dendrogram, likely exhibiting distinctive structural properties from the known anti-Mtb drugs.

## Discussion

### Results From the Screening of the HD-PBL Reflect Pathogen Specificities and Assay Characteristics

Virtual screening is an efficient strategy to bypass the hurdle of evaluating large libraries of compounds by selecting the supposedly best candidates, and reducing the number of *in vitro* and *in vivo* experiments. *Mycobacteria* and *Legionella* infections are described as a complex and dynamic series of interactions between multiple host and bacterial components and pathways. In order to create our HD-PBL, we selected a total of 18 host and pathogen pathways as potential pharmacological targets, and an LVS of the ZINC database was launched to identify ligands/metabolites known to interfere/interact with these pathways. In our study, we tested 1255 compounds from the ZINC lead-like database derived from a unique LVS to determine their anti-infective properties. For this purpose, a combination of three phenotypic assays was used, as summarized in Table 2. Two assays monitored the intracellular growth of (1) *M. marinum* and (2) *L. pneumophila* in *A. castellanii* and identified antibiotics, potential anti-virulence, and host defense boosters. In parallel, we used a phagocytic plaque assay (3), in which a compound restoring growth of *D. discoideum* on a lawn of Klebsiella *pneumoniae* spiked with *M. marinum* has either selective antibiotic activity against *M*. *marinum*, or attenuates the virulence of infecting *M. marinum.*

We identified 64 compounds showing activity in at least one of the assays for intracellular bacteria growth inhibition, or attenuation of mycobacterial virulence (Supplementary Table S1). Although the same host was used for the two infection assays, only few hits were common against *M. marinum* and *L. pneumophila*. The number of identified hits was considerably higher for *L. pneumophila* compared to *M. marinum* (Figures 5A,B). While no strong antibiotic hit was identified using the AcMm model, almost half of the hits identified in the AcLp screen (16/35) are potent antibiotics. A possible explanation might be the difference in growth rate between the two bacteria. *L. pneumophila* grows almost unrestrictedly inside the amoeba, with a doubling time close to 3 h, whereas *M. marinum* is a considerably slower grower, with a doubling time of around 8 h. A related aspect is the temperature (*T* = 25°C) used for the intracellular assays, which is closer to the optimum for *L. pneumophila*. Another plausible reason might be that the two pathogens have a very distinct cell wall composition. In contrast to the Gram-negative *L. pneumophila* cell wall, *M. marinum* has an elaborate and highly hydrophobic structure with unique components such as arabinogalactan, a highly branched polysaccharide that connects the peptidoglycan with the outer mycolic acid layer, strongly limiting the permeability to compounds. In addition, cellular components like efflux pumps might also play an important role in the bioavailability of the compounds. Another aspect is the nature of the bacteria proliferation niche. While *L. pneumophila* resides and proliferates in an ER-associated vacuole, *M. marinum* starts proliferating in a phagosome-derived vacuole and then continues after escape to the cytosol.

Surprisingly, the two assays used to identify anti-infective or anti-virulence compounds against *M. marinum* did not identify any common hit. As presented in Table 2B (first and second column), hits hallmarks are linked to the intrinsic screen design. Indeed, in the two infection assays, the compounds are added post-infection, and therefore, compounds that hamper bacteria uptake cannot be detected. Thus, both assays detect anti-infective hits that inhibit virulence or boost host defenses. On the contrary, in the anti-virulence assay (third column, Table 2), the uptake efficiency of *M. marinum* might be directly affected by compounds that either target the host phagocytosis machinery, or modify the mycobacterial cell wall. In addition, 11 of the 15 anti-virulence hits identified in the phagocytic plaque assay had selective antibiotic activity against *M. marinum* (Figure 7C).

To better understand the contrasting results of the anti-infective and the anti-virulence screens on *M. marinum*, the hits from both primary assays were re-tested in the DdMm infection assay (Table 2, first column and Figure 8A). Satisfyingly, two-thirds of the anti-infective hits originally identified in the *A. castellanii* assay were confirmed using the *D. discoideum* infection assay, likely reflecting the overall conservation of basic metabolic pathways between these two evolutionarily close organisms. In contrast, only half of the anti-virulence hits showed mild but significant activity in the *D. discoideum* infection assay (Figure 8B). One possibility is the presence of an MCV membrane around *M. marinum* that restricts the bioavailability of the compounds. In addition, it is known that *M. marinum* undergoes drastic metabolic adaptations to different carbon sources when transitioning between its extracellular life to the intracellular environment, possibly explaining the differential sensitivity to the anti-virulence hits.

### Pro-infective Hits, Another Facet to Better Understand the *Mycobacterium*–Host Interactions

On the other hand, identification of infection enhancers in the set of compounds was quite surprising, although the same observation was already reported in the phenotypic screen of the GSK TB set of anti-mycobacterial antibiotics in the AcMm model of infection (Trofimov et al., 2018). Notably here, five compounds were identified that increased the intracellular *M. marinum* load at least twofold (Figure 5A). As discussed in the Trofimov et al.’s paper, such compounds may be targeting and disarming crucial anti-bacterial host defense pathways and therefore, might lead to a better understanding of these pathways and might ultimately lead to the design of host-directed anti-mycobacterial therapies.

### Confirmation of the Low Cytotoxicity of Compounds on *D. discoideum*

Measurement of the hits’ cytotoxicity/growth inhibition activity was performed by monitoring the growth of GFP-expressing *D. discoideum*. Four out of six compounds that are either toxic for *D. discoideum* or affect its growth are common between two screens (Figures 6A–C). This low number of toxic compounds might be explained by the fact that the pathways selected for the LVS are non-essential for host metabolism and survival. In addition, the LVS likely enriched the library for compounds with drug-like properties that are anticipated to have low toxicity. The anti-infective hits with mild cell growth inhibition activity should be further investigated to optimize their therapeutic window.

### Navigation in the Biologically-Relevant Chemical Space Identifies a Potential Structural Class of Anti-mycobacterial Compounds

The chemical entities that were prioritized for biological assessment encompassed a large chemodiversity. In order to visualize the various physico-chemical properties of the tested compounds, we computed these characteristics using the eight-dimensional standardized space of ChemGPS-NP. Although this analysis did not show a clear separation between the hits and non-hits in each library, two groups of compounds formed distinct clusters, one containing *M. marinum* and anti-*L. pneumophila* hits (Box A) and a second one containing solely anti-*M. marinum* hits with the exception of one compound which belongs to the anti-*L. pneumophila* hits (Box B). One might speculate that Box B highlights compounds that hit mycobacteria-specific targets, while Box A either contains compounds that hit targets common to both bacteria or that enhance the activity of host defenses active against both bacteria. In parallel, the hierarchical classification identified a promising group of hits clustered away from the standard and candidate anti-Mtb drugs, suggesting the existence of a new group of anti-Mtb compounds, which need to be further studied. This analysis suggests that it might be interesting to use the ChemGPS-NP analysis to instruct the initial compound selection and the prioritization steps, notably by cluster analysis and neighborhood mapping. Mapping of collections of compounds with reported bioactivities and those discovered in our studies on such a chemical space will allow to partly orient the research and can also support SAR studies.

### Combination of 3R Model Assays Increases the Chances of Identifying Potential Anti-mycobacterial Compounds

Virtual screening is an efficient traditional strategy for quick evaluation of large libraries of compounds that permits a focus on the supposedly best candidates, reducing the amount of *in vitro* and *in vivo* experiments. It has become an integral part of the drug discovery process, with proven value in several therapeutic areas. In conclusion, our data show that the LVS efficiently selected anti-bacterials from the 2.5 Mio lead-like compounds of the ZINC library, giving rise to hit rates two to three times superior to the 1% usually observed by random screening. The data also demonstrate that the validated virtual hits are chemically diverse, suggesting that they most likely target different pathways within the host pathogen system. We suggest that our combination of cost-effective, 3R compliant amoebae-based phenotypic assays to screen structurally diverse chemical libraries efficiently identifies a variety of promising non-toxic anti-infective compounds that then will be validated in more complex infection systems such as the zebrafish or mouse models.

## Materials and Methods

### Design and Characterization of the Chemically Highly Diverse Pathway-Based Library for Phenotypic Screening

In recent years, the design of diverse libraries based on the principle of functional diversity has become a major trend in library design (Shelat and Guy, 2007). This includes designing libraries containing privileged structures as well as diverse scaffolds to best cover the chemical space. In this work, we selected in total 18 host and pathogen pathways (Table 1) as potential pharmacological targets to develop pharmacophores queries based on ligands/metabolites known to interfere/intervene within these host and pathogen pathways (Table 1). These pharmacophore queries have been used in LVS of the ZINC lead-like database^3^ (Sterling and Irwin, 2015) using ROCS, a tool from the OpenEye software package^4^ (Swann et al., 2011). The ligand-based VS was performed with ROCS using previously published default settings (Kirchmair et al., 2009) and the TanimotoCombo score. To ensure chemical diversity and maximize the coverage of the chemical space of the ZINC lead-like database, we applied the Lingo method program (Vidal et al., 2005). We screened the ZINC lead-like database composed of 2.5 million compounds and finally selected 100 compounds per host and pathogen pathways for a total of 1800 compounds using the workflow described in Figure 1. Thus, the selected virtual hits correspond to 0.07% of the whole ZINC lead-like database. The number of virtual hits was tractable by the medium throughput assays described in this work.

The pathways-based library properties were characterized in term of chemical diversity and drug likeness. To assess chemical diversity, we used the Z-matrix calculated according to Tanimoto’s chemical similarity metrics (*T*_sim_) using Canvas, a tool from the Schrödinger software package (Sindhikara and Borrelli, 2018). The results have been displayed as a heat map drawn using Netwalker1.0 (Komurov et al., 2012). The drug-likeliness of compounds of the library was assessed by predicting physico-chemical descriptors using Canvas (Ghose et al., 1999) and comparing them with the different known rules in drug discovery (Lipinski et al., 1997; Lipinski and Hopkins, 2004; Lipinski, 2016).

### Bacterial and Cell Cultures

*Acanthamoeba castellanii* (ATCC 30234) was grown in PYG medium at 25°C as described (Moffat and Tompkins, 1992; Segal and Shuman, 1999) using proteose peptone (Becton Dickinson Biosciences) and yeast extract (Difco). The *D. discoideum* strain was grown in HL5c medium at 22°C.

*Legionella pneumophila* were re-suspended from plates in appropriate growth medium, ACES Yeast Extract (AYE), and diluted to a starting OD_600_ of 0.01. Compounds were added to these cultures such that the maximal DMSO concentration was 0.1%. Cultures were grown overnight and the OD_600_ was measured.

*Mycobacterium marinum* were cultured in Middlebrook 7H9 (Difco) supplemented with 10% OADC (Becton Dickinson), 0.5% glycerol, and 0.2% Tween80 (Sigma–Aldrich) at 32°C in shaking culture. The *M. marinum* strain constitutively expressing GFP was obtained by transformation with msp12:GFP, and cultivated in the presence of 30 μg/ml kanamycin.

### Intracellular Replication of *M. marinum* in *A. castellanii*

*Acanthamoeba castellanii* were cultured in PYG medium in 10 cm Petri dishes at 25°C, and passaged the day prior to infection to reach 90% confluence. *M. marinum* were cultivated in a shaking culture at 32°C to an OD_600_ of 0.8–1 in 7H9 medium. Mycobacteria were centrifuged at RT at 500 *g* for two periods of 10 min onto a monolayer of *Acanthamoeba* cells at an MOI of 10 to promote efficient and synchronous uptake, followed by an additional 20–30 min incubation. Un-ingested bacteria were washed off with PYG and infected cells re-suspended in PYG containing 10 μM amikacin; 5 × 10^4^ infected cells were transferred to each well of a 96-well plate (Cell Carrier, black, transparent bottom from Perkin Elmer) with pre-plated compounds and controls. The course of infection at 25°C was monitored by measuring fluorescence in a plate reader (Synergy H1, BioTek) for 72 h with time points taken every 3 h. Only experiments with a *Z*-factor > 0.6 (calculated from DMSO and rifabutin controls) were taken into account for analysis. Time courses were plotted and data from all time points (using cumulative curves) were used to determine the effect of compounds versus vehicle controls. The primary hit rate cut off was set at 20% inhibition for *M. marinum*.

### Intracellular Replication of *M. marinum* in *D. discoideum*

*Dictyostelium discoideum* were cultured in HL5c medium in 10 cm Petri dishes at 22°C, and passaged the day prior to infection to reach 90% confluency. Mycobacteria were grown in 7H9 medium to a density of OD_600_ = 0.8–1.0 (5 × 10^8^ bacteria ml^–1^), centrifuged and re-suspended in HL5c medium and clumps disrupted by passaging through a 25−gauge needle. GFP-expressing *M. marinum* were added at an MOI of 10 and centrifuged onto the *Dictyostelium* cells at 500 *g* twice for 10 min. The cells were left at 25°C for an additional 10–20 min before uningested bacteria were washed off by three washes with HL5c and attached cells were then re−suspended in HL5c containing 10 μM amikacin. The course of infection was monitored as described above.

### Intracellular Replication of *L. pneumophila* in *A. castellanii*

*Acanthamoeba castellanii* were cultured in PYG medium at 25°C, and passaged the day prior to infection such that 2 × 10^4^ cells were present in each well of a 96-well plate (Cell Carrier, black, transparent bottom from Perkin Elmer). Cultures of *L. pneumophila* harboring the GFP-producing plasmid pNT-28 (Tiaden et al., 2007) were re-suspended from plate to a starting OD_600_ of 0.1 in AYE medium, and grown overnight in shaking conditions at 37°C to an OD_600_ of 3. Re-suspended bacteria in LoFlo medium (ForMedium) were centrifuged onto a monolayer of *A. castellanii* cells at an MOI of 20 to promote efficient and synchronous uptake. Compounds were added to at least triplicate wells after infection, and infected cells were incubated at 30°C. GFP fluorescence was measured by a plate spectrophotometer at appropriate intervals (Optima FluoStar, BMG Labtech). Because the culture media used for *A. castellanii* do not support the growth of *L. pneumophila*, GFP fluorescence accurately reflects intracellular replication. The hit rate cut off was set at 40% inhibition for *L. pneumophila*. Time courses were constructed, and data were used to determine the effect of compounds versus vehicle control.

### Anti-virulence Assay Against *M. marinum*

To test the effect of the compounds on *M*. *marinum* virulence, 10 ml of mid-log phase mycobacterial cultures (OD_600_ around 0.8–1.2) were pelleted by centrifugation and re-suspended in 5 ml of an overnight culture of *K*. *pneumoniae* (KpGe; Lima et al., 2018) diluted to 1/10,000 in LB medium (Alibaud et al., 2011). The mixture was de-clumped by passaging through a 25-gauge blunt needle. In each well of a 24-well plate, 10 μM of each compound was added and allowed to diffuse on 2 ml of solid standard medium (SM) agar supplemented with glucose followed by the addition of 50 μl of the bacterial suspension Once dried, 1000 *D. discoideum* cells were added in the center of the well. Plates were incubated for 5–9 days at 25°C and the formation of phagocytic plaques was monitored visually, a negative control (Bacteria + *D. discoideum* + DMSO) was included in every plate.

### Antibiotic Activity Assays

Antibiograms used to monitor the inhibitory effect of compounds on mycobacterial growth were performed as described previously (Ouertatani-Sakouhi et al., 2017), each molecule was added in a 24-well plate well containing 2 ml of 7H11 agar medium at 10 μM. Once dried, 1000 bacteria were deposited in each well and plates were incubated at 32°C for 7 days to allow bacterial growth.

To monitor *M. marinum* growth, GFP-producing bacteria were cultivated in shaking at 32°C in 7H9 medium supplemented with OADC up to an OD_600_ of 0.8–1. 10^5^ GFP-producing *M. marinum* were transferred into each well of 96-well white plates. To monitor *L. pneumophila* growth, a pre-culture of GFP-producing bacteria was diluted to a starting OD_600_ of 0.01 and grown overnight. Compounds at 30 μM in DMSO were added in each well, seeded with 100 μl of pre-culture, and bacterial growth was monitored for at least 48 h by measuring the fluorescence in a plate reader (Synergy H1) every 3 h.

### *D. discoideum* Growth Inhibition Assay

10^4^ GFP-ABD-expressing *Dictyostelium* cells were transferred to each well of 96-well plates allowed to attach for 20–30 min. Cell growth at 25°C was monitored by measuring the GFP fluorescence in a fluorescent plate reader (Synergy H1, company) for 72 h with the time point every 3 h.

### Cytotoxicity Assay

Cytotoxicity of compounds against *A. castellanii* was determined using the Alamar Blue reagent (Life Technologies). To mimic the conditions found in the intracellular replication assay, 96-well plates were set up as previously described and uninfected triplicate wells were treated with compounds in 100 μl LoFlo media (Harrison et al., 2013). Plates were incubated at 30°C for 24 h, after which 10 μl Alamar Blue reagent was added, and plates incubated for a further 3 h. The fluorescence at 595 nm was measured, and data normalized between 1 (treatment with LoFlo alone) and 0 (SDS, total lysis of the cells). Means from each individual experiment were then combined for analysis.

### ChemGPS Analysis

ChemGPS-NP is a PC analysis (PCA)-based model that serves as a tool for navigation in the biologically-relevant chemical space. It is composed of eight PCs based on 35 chemical descriptors, which represent physico-chemical properties such as size, shape, flexibility, rigidity, polarizability, lipophilicity, polarity, and hydrogen bonding capacity^5^ (Larsson et al., 2007; Rosen et al., 2009). The prediction scores were calculated based on the structural information derived from SMILES using the ChemBioDraw software. All salts, hydration information, and counterions were excluded from the SMILES annotation, and differences in stereochemistry ignored, since ChemGPS-NP only uses two-dimensional descriptors. For visualization in 2D, tSNE was applied to the eight output PCs from ChemGPS-NP on the ISDB library, standard and candidate anti-Mtb drugs, the GSK TB set, and the HD-PBL. In a second approach, tSNE was applied on the above libraries, excluding the ISDB reference library. The tSNE computation was performed using a perplexity value of 50 and a maximum number of iterations of 500. To ensure reproducibility, a fixed seed was used for tSNE, as well as for random subsampling the large ISDB reference library (Maaten and Hinton, 2008). For visualization, the resulting dimensions were represented in a scatter plot, and complemented by a 2D density estimation (density_stat_2d function of package ggplot2). The following programs, packages, and versions were used: R (version 3.6.0), Rstudio (version 1.2.1335), package Rtsne (version 0.15), package dplyr (version 0.8.3), package ggplot2 (version 3.2.1), package MASS (version 7.3-51.4).

### Data Analysis

Data analysis was performed using Microsoft Excel and GraphPad Prism 7. To compare the effect of compound treatment on intracellular replication, fluorescence values were taken from the first time point following entry to stationary phase. The results were then normalized such that media-only wells (no bacteria) were “0,” while vehicle-treated wells were “1” (normal replication). The average of the replicate wells (minimum three per plate) was then plotted as dose–response curves, such that each individual point represented the average of a single experiment. Compound treatments were repeated a minimum of three times to control for the increased variability of bacteria–host cell interactions.

## Author’s Note

This manuscript has been released as a Pre-Print at BioRxiv (Kicka et al., 2018).

## Data Availability Statement

All datasets generated for this study are included in the article/Supplementary Material.

## Author Contributions

TS, LS, HH, and PC conceived, designed, and supervised the work. NH, SK, CH, HS, VT, JN, and MP performed the experiments and analyzed the data. GC and AK provided the HD-PBL compounds. NH, TS, and SK structured and drafted the manuscript. All authors reviewed and edited the manuscript.

## Conflict of Interest

MP was employed by Swiss Institute of Bioinformatics (SIB). The remaining authors declare that the research was conducted in the absence of any commercial or financial relationships that could be construed as a potential conflict of interest.

## Footnotes

1 http://zinc.docking.org/

2 http://eyesopen.com/rocs

3 http://zinc.docking.org/

4 http://eyesopen.com/rocs

5 http://chemgps.bmc.uu.se

## Supplementary Material

TABLE S1 Primary screen data of the 1255 compounds in *A. castellanii–M. marinum* and *A. castellanii–L. pneumophila* models.

TABLE S2 Hit compounds characteristics: anti-infective, antibacterial, cytotoxic, and growth inhibitory data.
