# Supplementary material for: Identification of Anti-Mycobacterium and Anti-Legionella Compounds With Potential Distinctive Structural Scaffolds From an HD-PBL Using Phenotypic Screens in Amoebae Host Models
Source: Front Microbiol. 2020 Feb 21;11:266. doi: 10.3389/fmicb.2020.00266 (PMC7047896; doi:10.3389/fmicb.2020.00266)
Supplement: TABLE S1 — Primary screen data of the 1255 compounds in A. castellanii–M. marinum and A. castellanii–L. pneumophila models. [file Data_Sheet_1.PDF]

				A. castellanii-M. marinum			*A. castellanii- L. pneumophila*	
**ZINC Number**	**Mol Weight**	**Formula**	**Pathway**	**Mean (30 mM)**	**Stdev (30 mM)**		**Mean (30 mM)**	**Stdev (30 mM)**
ZINC00001409	283.156	C12H12Cl2N4	Cations channels	1.00	0.12		0.90	0.08
ZINC00001723	269.13	C11H10Cl2N4	Cations channels	1.02	0.02		1.11	0.22
ZINC00002433	258.39	C17H24NO	Proteasome inducers	0.90	0.05		0.99	0.13
ZINC00040832	263.342	C12H21N7	Calcineurin1	0.97	0.15		0.35	0.44
ZINC00042485	177.2197	C11H13O2	CYS path	0.89	0.03		0.94	0.09
ZINC00046208	217.27	C10H9N4S	TRP path	0.88	0.04		0.62	0.09
ZINC00046618	297.355	C16H19N5O	MeC	0.80	0.14		0.89	0.04
ZINC00047506	266.2946	C16H14N2O2	Cations channels	1.01	0.04		1.02	0.09
ZINC00047507	266.2946	C16H14N2O2	Cations channels	0.96	0.02		0.99	0.10
ZINC00051426	197.2325	C13H11NO	E3 ligase	0.87	0.18		1.26	0.14
ZINC00052010	271.2117	C11H7N6O3	PI3K	0.99	0.08		0.93	0.05
ZINC00052719	341.3612	C18H19N3O4	PI3K	1.00	0.11		1.00	0.11
ZINC00054122	321.3764	C18H19N5O	MeC	1.19	0.20		0.98	0.14
ZINC00054128	307.3498	C17H17N5O	MeC	1.19	0.05		0.93	0.07
ZINC00055632	311.3815	C17H21N5O	MeC	0.96	0.12		1.12	0.13
ZINC00055641	269.3018	C14H15N5O	MeC	0.81	0.09		0.96	0.06
ZINC00057042	207.1018	C8H3F4O2	CYS path	1.02	0.09		0.78	0.17
ZINC00057065	219.3458	C14H23N2	TRP path	0.93	0.02		0.89	0.07
ZINC00057464	249.719	C12H14ClN4	Cations channels	1.60	0.21		0.97	0.17
ZINC00058285	184.2371	C12H12N2	TRP path	0.78	0.09		0.70	0.17
ZINC00058608	302.3003	C16H15FN2O3	PI3K	0.95	0.12		1.03	0.10
ZINC00058693	333.77	C16H16ClN3O3	PI3K	1.02	0.07		0.97	0.14
ZINC00058795	254.521	C7H2Cl3NOS	AKT1	1.23	0.36		0.01	0.01
ZINC00061012	280.608	C10H6ClF3NO3	TRP path	1.12	0.10		0.04	0.04
ZINC00065540	300.3125	C15H16N4O3	Proteasome inducers	0.93	0.09		1.03	0.14
ZINC00068486	276.3309	C15H20N2O3	TRP path	0.97	0.06		1.06	0.13
ZINC00068652	275.151	C12H14Cl2NO2	ATPase	0.67	0.06		0.46	0.18
ZINC00072461	328.3657	C17H20N4O3	MeC	0.94	0.05		0.95	0.09
ZINC00072896	286.3042	C15H15FN4O	MeC	0.91	0.07		1.01	0.07
ZINC00073267	314.405	C16H18N4OS	MeC	1.00	0.08		1.00	0.12
ZINC00074160	197.1895	C12H7NO2	E3 ligase	1.09	0.14		1.19	0.33
ZINC00074639	344.3552	C19H18F2N2O2	GLN path	0.79	0.13		0.95	0.10
ZINC00078723	337.76	C18H12ClN3O2	E3 ligase	0.96	0.09		0.94	0.10
ZINC00079109	283.345	C17H19N2O2	TRP path	0.93	0.13		0.90	0.05
ZINC00081250	305.2677	C16H9N4O3	PI3K	1.00	0.07		1.04	0.11
ZINC00086324	296.149	C14H11Cl2NO2	TRP path	1.03	0.11		1.02	0.10

ZINC00087028	252.743	C12H17ClN4	Calcineurin1	0.93	0.01	0.45	0.13
ZINC00087389	276.3806	C14H24N6	Calcineurin1	0.96	0.03	0.01	0.02
ZINC00087528	203.2835	C12H17N3	Calcineurin1	0.87	0.10	1.11	0.06
ZINC00087935	321.3285	C15H19N3O5	HIS path	0.95	0.10	1.07	0.15
ZINC00090518	220.579	C8H4ClF3N2	AKT1	1.04	0.06	0.80	0.03
ZINC00091288	255.122	C7H8Cl2N2O2S	Cations channels	1.00	0.04	0.98	0.07
ZINC00094788	270.31	C13H10N4OS	Calcineurin1	0.97	0.09	1.01	0.06
ZINC00097660	289.3495	C16H21N2O3	TRP path	0.93	0.18	1.05	0.05
ZINC00098993	224.346	C10H12N2S2	Autophagy	0.89	0.02	1.01	0.04
ZINC00104010	254.2839	C15H14N2O2	Lip	1.07	0.04	1.01	0.09
ZINC00107866	230.067	C10H7Cl2O2	CYS path	1.05	0.03	0.76	0.20
ZINC00109133	270.2866	C14H14N4O2	MeC	0.85	0.06	1.04	0.07
ZINC00112851	324.377	C18H20N4O2	Proteasome inducers	0.93	0.05	1.16	0.25
ZINC00120276	209.2002	C13H7NO2	AKT1	0.69	0.16	0.77	0.19
ZINC00127447	213.2319	C13H11NO2	Proteasome inducers	1.21	0.20	0.51	0.20
ZINC00128896	273.327	C16H19NO3	Proteasome I-lact-mg 132	0.99	0.09	0.93	0.27
ZINC00132651	201.2676	C12H15N3	Autophagy	0.87	0.20	1.04	0.12
ZINC00133390	210.228	C14H10O2	Lip	0.82	0.03	0.96	0.06
ZINC00136815	288.368	C14H16N4OS	MeC	0.89	0.03	1.07	0.09
ZINC00140499	343.4466	C18H27N6O	ATPase	0.95	0.06	1.03	0.13
ZINC00140791	347.3673	C20H17N3O3	PI3K	0.78	0.06	1.00	0.11
ZINC00141521	262.2182	C12H10N2O5	E3 ligase	0.96	0.06	0.86	0.33
ZINC00144184	215.2942	C13H17N3	Autophagy	1.06	0.05	0.87	0.07
ZINC00147843	314.3391	C16H18N4O3	Proteasome inducers	0.98	0.12	0.90	0.22
ZINC00153185	320.3603	C17H22NO5	CYS path	0.99	0.05	1.02	0.08
ZINC00153191	338.399	C16H20NO5S	CYS path	0.99	0.02	0.98	0.13
ZINC00153784	337.26	C13H10F3N7O	ATPase	1.03	0.03	0.97	0.10
ZINC00159307	266.508	C9H6Cl3NO2	Autophagy	1.47	0.16	0.70	0.23
ZINC00159566	332.4222	C20H22N5	HIS path	1.34	0.19	1.04	0.15
ZINC00163222	333.4219	C22H22FN2	GLN path	0.95	0.07	0.72	0.10
ZINC00164027	214.24	C12H6O2S	AKT1	0.70	0.15	0.92	0.23
ZINC00168806	254.2839	C15H14N2O2	TRP path	0.95	0.10	1.08	0.22
ZINC00173234	250.317	C12H14N2O2S	Proteasome inducers	0.99	0.02	0.97	0.11
ZINC00178917	201.2643	C13H15NO	TRP path	0.97	0.11	1.13	0.09
ZINC00179289	286.3042	C15H15FN4O	MeC	1.01	0.01	0.98	0.08
ZINC00179334	255.2752	C13H13N5O	MeC	0.77	0.09	0.90	0.05
ZINC00180129	328.3657	C17H20N4O3	MeC	1.00	0.07	0.93	0.05
ZINC00182876	288.075	C9H8BrN2O4	Proteasome I-lact-mg 132	0.92	0.06	0.91	0.08

ZINC00186403	284.3131	C15H16N4O2	MeC	0.84	0.09	0.84	0.08
ZINC00186442	284.3131	C15H16N4O2	MeC	1.12	0.08	1.14	0.11
ZINC00186461	284.3131	C15H16N4O2	MeC	1.02	0.08	1.06	0.15
ZINC00186586	312.3663	C17H20N4O2	MeC	0.91	0.08	0.88	0.10
ZINC00192269	284.3131	C15H16N4O2	MeC	2.32	0.93	0.88	0.06
ZINC00198367	325.3585	C19H19NO4	E3 ligase	1.01	0.02	1.08	0.23
ZINC00198854	248.2143	C13H13O3P	Autophagy	0.89	0.00	0.99	0.06
ZINC00204973	238.2878	C14H14N4	Calcineurin1	1.11	0.09	1.03	0.17
ZINC00208567	255.239	C12H12F3N3	Calcineurin1	0.89	0.07	1.08	0.05
ZINC00221036	327.4207	C19H25N3O2	HIS path	1.06	0.03	1.05	0.14
ZINC00222799	339.3685	C18H19N4O3	E3 ligase	0.92	0.05	0.82	0.16
ZINC00223182	331.363	C18H21NO5	Proteasome inducers	1.02	0.05	1.00	0.09
ZINC00230833	331.3663	C17H21N3O4	PI3K	1.08	0.04	1.07	0.04
ZINC00230874	301.3404	C16H19N3O3	PI3K	1.00	0.00	1.06	0.09
ZINC00231436	193.242	C10H9O2S	CYS path	0.92	0.09	1.09	0.14
ZINC00233904	269.3184	C16H17N2O2	TRP path	0.94	0.03	0.96	0.16
ZINC00241106	328.3624	C18H20N2O4	TRP path	1.00	0.07	0.88	0.09
ZINC00244312	295.2695	C16H11N2O4	TRP path	0.90	0.06	0.92	0.05
ZINC00254142	338.3324	C19H15FN2O3	Lip	0.94	0.07	0.91	0.10
ZINC00254148	338.3324	C19H15FN2O3	Lip	0.91	0.08	0.91	0.25
ZINC00259013	296.3868	C18H22N3O	ATPase	1.04	0.08	0.93	0.12
ZINC00266162	268.334	C15H12N2OS	E3 ligase	0.95	0.06	0.92	0.07
ZINC00266835	282.294	C16H14N2O3	HIS path	1.10	0.09	1.11	0.13
ZINC00266878	342.3459	C18H18N2O5	HIS path	1.04	0.07	0.85	0.39
ZINC00268590	250.2936	C13H18N2O3	Proteasome I-lact-mg 132	0.81	0.02	0.93	0.11
ZINC00272282	213.1723	C10H5N4O2	TRP path	1.24	0.13	1.30	0.23
ZINC00281753	286.0692	C9H7IN2O	Autophagy	1.12	0.04	1.15	0.07
ZINC00284706	251.3214	C14H21NO3	PI3K	0.97	0.02	1.20	0.13
ZINC00285062	339.3453	C18H17N3O4	HIS path	1.11	0.13	0.98	0.03
ZINC00297902	274.552	C7H6Cl3NO2S	TRP path	0.95	0.06	0.89	0.09
ZINC00299967	311.3782	C18H21N3O2	Calcineurin1	0.79	0.05	1.05	0.07
ZINC00300148	276.3357	C17H16N4	HIS path	1.09	0.11	0.98	0.05
ZINC00300173	260.3977	C16H26N3	Calcineurin1	1.13	0.04	0.74	0.12
ZINC00300618	290.702	C14H11ClN2O3	HIS path	1.08	0.03	1.00	0.21
ZINC00301222	324.3737	C19H20N2O3	PI3K	1.06	0.08	0.59	0.27
ZINC00305099	296.749	C14H17ClN2O3	HIS path	0.88	0.03	0.87	0.16
ZINC00308444	328.3823	C19H22NO4	Proteasome inducers	0.95	0.00	1.11	0.20
ZINC00314072	229.301	C12H11N3S	AKT1	1.61	0.07	1.01	0.15

ZINC00315276	291.3952	C14H25N7	Calcineurin1	1.03	0.10	0.77	0.24
ZINC00317276	320.3452	C18H16N4O2	E3 ligase	0.89	0.02	1.04	0.18
ZINC00317278	311.3319	C18H17NO4	GLN path	0.84	0.10	1.34	0.34
ZINC00327565	288.388	C16H24N4O	Calcineurin1	1.03	0.06	1.04	0.05
ZINC00328023	298.3116	C17H15FN2O2	TRP path	0.86	0.02	1.04	0.05
ZINC00331183	197.665	C9H12ClN3	Calcineurin1	0.91	0.03	1.10	0.15
ZINC00331466	246.3712	C15H24N3	Calcineurin1	0.89	0.02	0.88	0.09
ZINC00331468	246.3712	C15H24N3	Calcineurin1	0.91	0.04	0.44	0.17
ZINC00331977	304.3609	C17H22NO4	CYS path	0.76	0.02	0.93	0.07
ZINC00335583	232.225	C9H7F3N2S	Proteasome I-lact-mg 132	1.05	0.02	0.83	0.28
ZINC00336105	313.3113	C15H15N5O3	MeC	0.98	0.02	1.06	0.25
ZINC00336117	314.3391	C16H18N4O3	MeC	1.04	0.04	0.90	0.06
ZINC00336277	229.298	C13H11NOS	Autophagy	0.97	0.05	0.48	0.27
ZINC00338895	173.2111	C11H11NO	AKT1	0.99	0.05	0.93	0.09
ZINC00342851	339.3453	C18H17N3O4	Proteasome I-lact-mg 132	1.02	0.06	0.78	0.20
ZINC00345858	183.2674	C11H19O2	CYS path	0.99	0.09	0.92	0.07
ZINC00347774	268.397	C17H18NS	Proteasome inducers	0.73	0.13	0.18	0.27
ZINC00350678	334.39	C16H18N2O4S	PI3K	1.04	0.01	1.10	0.07
ZINC00355604	341.3282	C16H18F3N3O2	Proteasome I-lact-mg 132	0.90	0.03	0.97	0.05
ZINC00368768	296.3205	C17H16N2O3	E3 ligase	0.95	0.10	0.94	0.13
ZINC00374682	284.3098	C16H16N2O3	Lip	1.00	0.00	0.92	0.24
ZINC00378072	324.324	C14H11F3N4S	E3 ligase	1.02	0.21	0.62	0.09
ZINC00385668	254.2806	C16H14O3	Lip	1.05	0.04	0.92	0.14
ZINC00386647	223.2698	C15H13NO	Lip	0.94	0.10	0.91	0.10
ZINC00386690	295.741	C14H16ClN2O3	CYS path	1.26	0.31	1.02	0.04
ZINC00386725	275.323	C15H19N2O3	CYS path	1.04	0.05	0.98	0.11
ZINC00389849	138.1638	C8H10O2	Proteasome I-lact-mg 132	0.99	0.01	0.90	0.04
ZINC00391674	229.3175	C15H19NO	Proteasome inducers	0.97	0.10	0.91	0.07
ZINC00393308	191.2463	C12H15O2	CYS path	0.92	0.03	0.99	0.07
ZINC00393738	206.2808	C13H18O2	FASII-PDIM metabolism	1.05	0.08	1.09	0.09
ZINC00393739	206.2808	C13H18O2	FASII-PDIM metabolism	1.09	0.14	0.96	0.36
ZINC00397632	224.2546	C15H12O2	Lip	1.19	0.04	1.06	0.18
ZINC00398524	250.2506	C12H14N2O4	E3 ligase	0.99	0.18	1.01	0.14
ZINC00401596	339.385	C20H21NO4	Proteasome I-lact-mg 132	0.99	0.10	0.84	0.17
ZINC00402777	300.3722	C18H22NO3	Proteasome inducers	0.99	0.14	0.94	0.11
ZINC00403600	312.3829	C19H22NO3	Proteasome inducers	1.01	0.06	0.94	0.10
ZINC00408670	251.2832	C15H13N3O	Proteasome inducers	1.03	0.09	1.05	0.17
ZINC00409927	229.2744	C14H15NO2	TRP path	0.84	0.01	1.03	0.11

ZINC00419037	264.3217	C17H16N2O	CD36	0.96	0.00	1.08	0.12
ZINC00435382	255.2752	C13H13N5O	MeC	0.82	0.14	0.91	0.06
ZINC00435386	314.3391	C16H18N4O3	MeC	0.88	0.12	0.96	0.02
ZINC00437832	260.719	C14H13ClN2O	Lip	0.96	0.12	0.94	0.06
ZINC00439984	257.2183	C14H9O5	TRP path	0.76	0.15	1.69	0.75
ZINC00458873	346.3825	C20H18N4O2	ATPase	1.01	0.06	0.96	0.04
ZINC00464461	290.3144	C15H18N2O4	HIS path	0.88	0.10	1.20	0.22
ZINC00470274	260.3779	C15H24N4	Calcineurin1	0.88	0.08	0.85	0.03
ZINC00474617	323.3211	C18H14FN3O2	MeC	1.11	0.15	1.05	0.13
ZINC00479991	211.2591	C14H13NO	AKT1	1.05	0.09	0.78	0.07
ZINC00485519	339.776	C18H14ClN3O2	MeC	1.27	0.08	1.01	0.17
ZINC00485539	339.776	C18H14ClN3O2	MeC	1.29	0.17	0.91	0.14
ZINC00487871	288.3615	C17H22NO3	Proteasome inducers	1.01	0.11	0.96	0.13
ZINC00488396	333.3175	C19H13N2O4	CYS path	0.98	0.04	0.99	0.18
ZINC00489468	345.3945	C21H19N3O2	E3 ligase	0.83	0.11	0.87	0.02
ZINC00493635	298.3364	C17H18N2O3	PI3K	1.12	0.11	0.94	0.04
ZINC00495214	269.406	C13H23N3OS	Proteasome I-lact-mg 132	1.03	0.03	0.97	0.14
ZINC00505373	237.2566	C14H11N3O	ABC transporters	1.48	0.39	0.96	0.16
ZINC00506492	263.723	C13H14ClN3O	AKT1	1.09	0.09	1.08	0.13
ZINC00507667	271.442	C13H21NOS2	Proteasome inducers	1.02	0.10	0.97	0.09
ZINC00517836	300.38	C18H26NO8P	Proteasome inducers	1.00	0.07	1.02	0.08
ZINC00521146	337.76	C18H12ClN3O2	E3 ligase	0.85	0.04	0.98	0.08
ZINC00521574	341.724	C17H9ClFN3O2	E3 ligase	1.04	0.01	0.98	0.17
ZINC00526730	301.3437	C15H19N5O2	Calcineurin1	1.08	0.04	0.98	0.10
ZINC00530430	256.278	C10H12N2O4S	Proteasome inducers	0.87	0.03	1.06	0.12
ZINC00532032	329.3968	C17H23N5O2	Calcineurin1	1.44	0.06	0.94	0.12
ZINC00549930	334.395	C18H14N4OS	PI3K	1.01	0.08	0.87	0.01
ZINC00551737	262.2613	C13H14N2O4	CD36	1.02	0.09	0.98	0.10
ZINC00554850	318.391	C16H18N2O3S	PI3K	0.93	0.08	1.07	0.14
ZINC00586997	298.3364	C17H18N2O3	PI3K	0.88	0.02	1.03	0.08
ZINC00619511	289.2833	C15H15NO5	Proteasome inducers	0.98	0.03	1.06	0.13
ZINC00757041	260.3944	C17H26NO	Autophagy	1.21	0.32	0.99	0.04
ZINC00790529	257.3276	C16H19NO2	TRP path	0.92	0.02	0.89	0.08
ZINC00818584	262.354	C13H22N6	Calcineurin1	0.98	0.08	-0.02	0.02
ZINC00831987	207.2689	C12H17NO2	Autophagy	1.00	0.07	1.12	0.15
ZINC00843023	266.3376	C17H18N2O	Lip	1.01	0.03	0.87	0.14
ZINC00843027	270.3015	C16H15FN2O	Lip	1.07	0.05	0.82	0.16
ZINC00873712	189.2105	C11H11NO2	CD36	0.84	0.09	1.05	0.23

ZINC00899897	420.46	C25H24O6		1.08	0.21	0.69	0.19
ZINC00967524	338.4449	C24H22N2	ATPase	0.83	0.13	0.84	0.12
ZINC00983974	193.2438	C14H11N	Autophagy	1.13	0.08	0.96	0.21
ZINC00988923	261.725	C10H12ClNO3S	TRP path	0.94	0.12	0.96	0.17
ZINC00988927	275.752	C11H14ClNO3S	TRP path	1.03	0.15	1.06	0.14
ZINC01031908	294.308	C16H14N4O2	PI3K	1.01	0.12	0.98	0.21
ZINC01036768	197.2325	C13H11NO	Autophagy	1.20	0.42	0.31	0.05
ZINC01048260	260.525	C6H4Cl3NO2S	Cations channels	0.99	0.11	0.71	0.04
ZINC01064111	250.2936	C13H18N2O3	Proteasome I-lact-mg 132	0.85	0.04	0.96	0.10
ZINC01072160	322.4009	C20H22N2O2	GLN path	0.94	0.05	0.96	0.05
ZINC01076000	191.2695	C12H17NO	Proteasome inducers	0.86	0.10	0.76	0.16
ZINC01076840	310.389	C15H20NO4S	PI3K	0.90	0.06	1.03	0.04
ZINC01106203	346.406	C19H14N4OS	HIS path	1.03	0.08	1.15	0.11
ZINC01226820	159.2276	C11H13N	AKT1	0.88	0.08	0.87	0.17
ZINC01244188	302.4327	C22H24N	E3 ligase	1.20	0.03	0.91	0.09
ZINC01386415	297.3517	C17H19N3O2	Calcineurin1	0.99	0.14	0.88	0.23
ZINC01386521	182.2212	C12H10N2	Proteasome inducers	1.07	0.04	0.17	0.12
ZINC01387152	276.722	C13H13ClN4O	AKT1	1.13	0.18	0.78	0.22
ZINC01387195	185.439	C4H3Cl3N2	Calcineurin1	0.99	0.07	1.05	0.11
ZINC01388874	192.043	C7H7Cl2NO	Proteasome I-lact-mg 132	0.94	0.10	0.85	0.19
ZINC01391153	338.788	C19H15ClN2O2	Lip	0.84	0.16	0.91	0.07
ZINC01393003	300.418	C17H20N2OS	Proteasome I-lact-mg 132	1.22	0.12	0.76	0.43
ZINC01393478	290.3193	C17H14N4O	ABC transporters	0.95	0.12	1.09	0.22
ZINC01394722	269.3167	C16H16FN3	Calcineurin1	1.09	0.07	0.96	0.05
ZINC01394939	268.3137	C15H16N4O	Calcineurin1	0.94	0.09	0.93	0.18
ZINC01400202	272.2743	C15H13FN2O2	Lip	0.60	0.08	0.89	0.16
ZINC01400204	288.729	C15H13ClN2O2	Lip	0.98	0.03	1.02	0.13
ZINC01402095	303.379	C15H17N3O2S	Calcineurin1	0.99	0.08	0.92	0.35
ZINC01403289	224.111	C6H7Cl2N3S	Calcineurin1	0.99	0.07	0.96	0.08
ZINC01406325	268.483	C6H6ClIN2	AKT1	0.99	0.02	0.91	0.04
ZINC01406332	233.697	C12H12ClN3	Autophagy	1.08	0.07	0.55	0.12
ZINC01420952	206.671	C11H11ClN2	Proteasome I-lact-mg 132	0.53	0.10	0.97	0.06
ZINC01422377	264.3202	C14H20N2O3	TRP path	0.84	0.05	0.96	0.10
ZINC01424657	337.3725	C19H19N3O3	ATPase	0.96	0.08	1.05	0.16
ZINC01424788	290.2697	C14H13FN3O3	CYS path	0.87	0.24	1.06	0.18
ZINC01431470	236.3068	C14H20O3	FASII-PDIM metabolism	1.00	0.05	1.08	0.16
ZINC01450902	282.551	C10H10Cl3NO2	TRP path	0.95	0.06	0.91	0.08
ZINC01462057	302.969	C9H7Cl4NO2	TRP path	1.04	0.13	0.96	0.12

ZINC01494930	131.1745	C9H9N	AKT1	0.85	0.09	1.03	0.07
ZINC01561630	261.3178	C18H15NO	E3 ligase	0.94	0.13	1.09	0.19
ZINC01562159	146.2737	C8H22N2	Porins	0.90	0.04	0.90	0.37
ZINC01562261	156.2652	C10H20O	TRP path	0.58	0.21	1.10	0.17
ZINC01562344	262.652	C11H7ClN4O2	E3 ligase	0.97	0.14	0.97	0.09
ZINC01566231	218.3147	C14H20NO	Proteasome inducers	0.91	0.11	1.10	0.08
ZINC01569058	275.54	C6H5Cl3N2O2S	Cations channels	0.90	0.04	0.92	0.15
ZINC01569224	297.288	C8H12NO5PS2	Cations channels	0.98	0.15	0.93	0.09
ZINC01572284	263.726	C12H14ClN5	Cations channels	1.09	0.12	0.83	0.29
ZINC01572285	305.763	C14H16ClN5O	Cations channels	0.92	0.04	0.99	0.18
ZINC01577064	176.2548	C12H16O	FASII-PDIM metabolism	1.01	0.05	0.90	0.12
ZINC01577065	176.2548	C12H16O	FASII-PDIM metabolism	0.98	0.05	1.18	0.24
ZINC01577066	176.2548	C12H16O	FASII-PDIM metabolism	1.02	0.06	0.91	0.07
ZINC01577507	189.2984	C9H23N3O	Porins	0.91	0.05	1.07	0.15
ZINC01578104	270.3477	C20H16N	E3 ligase	1.36	0.06	1.14	0.39
ZINC01580458	255.2686	C15H13NO3	Proteasome inducers	1.04	0.22	0.93	0.07
ZINC01580838	145.201	C10H11N	AKT1	1.21	0.08	0.91	0.19
ZINC01580881	270.2435	C13H10N4O3	E3 ligase	0.92	0.12	0.95	0.21
ZINC01584465	200.2994	C14H18N	Proteasome inducers	0.95	0.09	1.01	0.12
ZINC01588897	259.307	C13H17N5O	Cations channels	0.97	0.09	0.75	0.31
ZINC01589731	169.2408	C10H17O2	CYS path	0.90	0.05	0.99	0.04
ZINC01594493	219.2564	C13H15O3	CYS path	0.96	0.06	0.87	0.05
ZINC01595201	288.3814	C18H24O3	Proteasome inducers	1.04	0.24	1.10	0.34
ZINC01602637	247.72	C14H14ClNO	TRP path	1.36	0.37	1.07	0.14
ZINC01605940	192.2774	C12H18NO	Proteasome inducers	1.02	0.10	0.95	0.15
ZINC01615127	240.726	C13H17ClO2	Proteasome I-lact-mg 132	0.95	0.16	1.03	0.26
ZINC01615703	152.1904	C9H12O2	Proteasome I-lact-mg 132	0.77	0.02	0.97	0.04
ZINC01622457	300.3722	C18H22NO3	Proteasome inducers	0.85	0.04	0.99	0.09
ZINC01624894	271.525	C9H9Cl3O3	ATPase	1.54	0.84	1.07	0.19
ZINC01628259	229.2744	C14H15NO2	Autophagy	1.03	0.14	1.10	0.24
ZINC01633981	197.45	C5H3Cl3N2	Calcineurin1	0.92	0.12	0.90	0.10
ZINC01633982	197.45	C5H3Cl3N2	AKT1	1.06	0.10	0.60	0.30
ZINC01637881	253.547	C6H6BrClN2S	AKT1	0.90	0.06	0.90	0.26
ZINC01637916	274.875	C4HCl2IN2	AKT1	0.94	0.04	0.83	0.38
ZINC01639218	316.1964	C12H10F6O3	TRP path	0.87	0.08	0.95	0.13
ZINC01640468	293.3597	C19H19NO2	E3 ligase	0.86	0.02	0.95	0.17
ZINC01640897	190.2814	C13H18O	FASII-PDIM metabolism	1.03	0.09	1.00	0.18
ZINC01640921	132.2471	C7H20N2	Porins	0.87	0.03	0.88	0.11

ZINC01646265	188.2655	C13H16O	FASII-PDIM metabolism	1.12	0.08	0.95	0.13
ZINC01648047	300.352	C16H14NO3S	CYS path	0.96	0.08	0.91	0.17
ZINC01655941	215.634	C9H10ClNO3	Proteasome I-lact-mg 132	1.21	0.12	1.06	0.19
ZINC01662344	186.2728	C13H16N	Proteasome inducers	0.88	0.04	1.03	0.06
ZINC01668015	322.79	C15H19ClN4O2	Cations channels	0.99	0.05	1.11	0.26
ZINC01669858	212.716	C12H17ClO	TRP path	0.81	0.08	0.98	0.13
ZINC01670914	196.2014	C13H8O2	Proteasome inducers	0.94	0.11	0.98	0.15
ZINC01672144	183.423	C4HCl3N2	AKT1	1.04	0.08	1.08	0.13
ZINC01672145	255.927	C6H5BrCl2N2	CYS path	0.76	0.06	1.00	0.08
ZINC01672296	170.639	C8H11ClN2	Calcineurin1	0.98	0.14	0.96	0.09
ZINC01676192	225.1794	C7H16NO5P	CYS path	1.13	0.21	0.92	0.04
ZINC01677195	206.306	C8H18N2O2S	Porins	0.92	0.05	0.94	0.16
ZINC01677408	204.3098	C10H24N2O2	Porins	0.89	0.08	0.87	0.25
ZINC01677603	250.2969	C12H18N4O2	Calcineurin1	0.97	0.11	1.06	0.09
ZINC01677613	246.3513	C14H22N4	Calcineurin1	1.17	0.13	0.98	0.15
ZINC01683094	188.3534	C11H28N2	Porins	0.88	0.04	0.88	0.13
ZINC01683101	274.679	C14H9ClNO3	TRP path	0.88	0.07	1.06	0.04
ZINC01683178	303.161	C13H14Cl2NO3	CYS path	0.82	0.08	1.06	0.10
ZINC01683255	229.2744	C14H15NO2	TRP path	1.20	0.04	0.96	0.12
ZINC01689041	264.2754	C17H12O3	TRP path	0.94	0.02	0.87	0.02
ZINC01689136	177.031	C6H6Cl2N2	AKT1	0.77	0.05	0.95	0.02
ZINC01689137	191.058	C7H8Cl2N2	AKT1	0.87	0.07	0.98	0.16
ZINC01689138	191.058	C7H8Cl2N2	AKT1	0.90	0.04	0.90	0.09
ZINC01689789	238.4121	C15H30N2	Porins	0.95	0.04	1.07	0.16
ZINC01692386	184.229	C6H13FO3S	CYS path	0.73	0.03	0.91	0.11
ZINC01694018	340.129	C12H10BrN3O4	E3 ligase	1.07	0.16	1.05	0.27
ZINC01698239	254.2872	C14H14N4O	Autophagy	0.79	0.06	0.98	0.07
ZINC01700031	257.2845	C15H15NO3	TRP path	0.99	0.06	0.98	0.06
ZINC01700279	276.3508	C16H22NO3	Proteasome inducers	1.25	0.19	1.07	0.09
ZINC01703515	257.2447	C13H11N3O3	E3 ligase	0.95	0.20	1.00	0.09
ZINC01707352	208.2536	C12H16O3	CYS path	1.01	0.02	0.93	0.08
ZINC01709464	254.2839	C15H14N2O2	Autophagy	1.01	0.20	1.17	0.26
ZINC01710046	198.3019	C12H22O2	CYS path	0.58	0.13	1.04	0.08
ZINC01710992	272.43	C19H28O	Proteasome I-lact-mg 132	0.99	0.01	0.82	0.07
ZINC01715810	208.32	C12H16OS	FASII-PDIM metabolism	1.00	0.09	0.98	0.23
ZINC01717751	272.2958	C16H16O4	Proteasome I-lact-mg 132	1.02	0.16	0.96	0.09
ZINC01718072	334.715	C14H11ClN4O4	E3 ligase	0.61	0.27	3.81	2.52
ZINC01723038	249.3486	C15H23NO2	Proteasome I-lact-mg 132	0.98	0.14	0.97	0.07

ZINC01723260	132.2471	C7H20N2	Porins	1.00	0.08	0.90	0.05
ZINC01728154	216.322	C14H20N2	Calcineurin1	0.98	0.14	0.97	0.20
ZINC01737769	258.51	C7H8Cl3N2O2	Proteasome I-lact-mg 132	0.15	0.06	0.57	0.26
ZINC01743197	263.315	C11H7N2O2S2	Cations channels	0.76	0.11	1.00	0.11
ZINC01743326	222.459	C6H2Cl3N3	AKT1	1.23	0.07	0.93	0.16
ZINC01744394	188.2655	C13H16O	FASII-PDIM metabolism	0.97	0.05	0.93	0.07
ZINC01746048	272.087	C10H7Cl2N3O2	E3 ligase	0.94	0.15	1.06	0.26
ZINC01751863	265.2634	C16H11NO3	E3 ligase	0.98	0.16	1.14	0.25
ZINC01765796	162.2283	C11H14O	FASII-PDIM metabolism	1.01	0.05	0.93	0.06
ZINC01787360	315.14	C12H13BrNO4	CYS path	0.75	0.13	0.91	0.08
ZINC01846079	272.321	C10H10NO4S2	CYS path	0.97	0.08	1.10	0.11
ZINC01865424	254.134	C8H9Cl2NO2S	Cations channels	1.12	0.05	0.99	0.10
ZINC01866749	167.2066	C12H9N	AKT1	0.92	0.08	0.86	0.11
ZINC01872262	348.377	C15H16N4O4S	HIS path	0.83	0.02	0.92	0.09
ZINC01873441	294.1095	C9H13INO2	Proteasome I-lact-mg 132	0.99	0.10	0.92	0.08
ZINC01972870	235.348	C12H17N3S	Autophagy	0.87	0.13	0.90	0.13
ZINC02004262	338.4202	C21H24NO3	Proteasome inducers	0.94	0.17	1.14	0.10
ZINC02009495	294.214	C9H8F6O2S	ATPase	1.44	0.30	0.94	0.12
ZINC02036274	169.2408	C10H17O2	CYS path	0.94	0.06	1.04	0.08
ZINC02042129	210.228	C14H10O2	Lip	0.95	0.05	0.90	0.15
ZINC02045475	167.2249	C10H15O2	CYS path	1.03	0.04	1.02	0.03
ZINC02048546	286.713	C15H11ClN2O2	Cations channels	0.89	0.04	0.95	0.06
ZINC02075095	258.502	C8H5BrClN3	Autophagy	1.50	0.04	0.47	0.22
ZINC02075448	252.1472	C6H4N8O4	PI3K	0.72	0.03	0.43	0.13
ZINC02096593	346.3545	C18H20NO6	CYS path	1.08	0.06	0.97	0.14
ZINC02097134	346.3545	C18H20NO6	CYS path	0.95	0.02	0.88	0.05
ZINC02146716	146.2737	C8H22N2	Porins	0.91	0.01	1.22	0.28
ZINC02159801	161.1989	C7H15NO3	Proteasome I-lact-mg 132	0.97	0.09	0.95	0.13
ZINC02168057	169.2224	C12H11N	Proteasome inducers	0.94	0.04	0.94	0.11
ZINC02238955	335.3533	C20H17NO4	Lip	1.03	0.10	1.00	0.10
ZINC02318582	293.318	C13H13N2O4S	CYS path	1.02	0.01	1.06	0.07
ZINC02365627	257.099	C8H2Cl2N4S	Calcineurin1	0.48	0.09	0.48	0.28
ZINC02382128	237.583	C9H5ClF3O2	CYS path	1.01	0.13	0.98	0.19
ZINC02395731	244.2659	C14H14NO3	TRP path	1.04	0.16	0.98	0.09
ZINC02407651	326.3896	C19H22N2O3	Proteasome I-lact-mg 132	1.06	0.09	1.08	0.17
ZINC02440825	208.1919	C8H17O4P	CYS path	0.99	0.07	1.04	0.12
ZINC02470286	262.3275	C14H20N3O2	ATPase	1.00	0.08	0.91	0.08
ZINC02472312	254.3269	C16H18N2O	Lip	0.96	0.09	0.94	0.10

ZINC02510358	252.268	C15H12N2O2	Cations channels	1.03	0.08	0.94	0.08
ZINC02516806	145.201	C10H11N	AKT1	1.35	0.05	0.88	0.06
ZINC02557704	259.2805	C14H15N2O3	CYS path	0.96	0.09	1.03	0.14
ZINC02558079	203.065	C9H8Cl2O	Cations channels	1.49	0.10	0.32	0.11
ZINC02558608	227.874	C4HBrCl2N2	Calcineurin1	0.93	0.06	0.73	0.14
ZINC02563372	188.3534	C11H28N2	Porins	0.93	0.14	1.05	0.16
ZINC02565661	256.023	C6H5BrF3N3	AKT1	0.92	0.06	1.00	0.10
ZINC02567990	146.2737	C8H22N2	Porins	0.99	0.11	0.87	0.05
ZINC02570138	191.055	C8H8Cl2O	TRP path	0.97	0.03	0.80	0.05
ZINC02571853	169.585	C8H6ClO2	CYS path	0.98	0.05	0.90	0.05
ZINC02572251	146.2737	C8H22N2	Porins	0.98	0.14	1.11	0.15
ZINC02643380	346.351	C12H9F3N4OS2	HIS path	0.87	0.10	0.92	0.24
ZINC02685033	318.368	C16H16NO4S	CYS path	1.03	0.10	0.96	0.06
ZINC02685796	268.3137	C15H16N4O	MeC	0.89	0.04	1.02	0.06
ZINC02739631	290.571	C9H14Cl3NO3	Proteasome I-lact-mg 132	1.11	0.04	1.10	0.06
ZINC02744073	233.1656	C9H7F4N3	Calcineurin1	0.96	0.07	1.17	0.12
ZINC02770044	333.3029	C15H18F3NO4	Proteasome I-lact-mg 132	1.01	0.11	0.91	0.12
ZINC02780124	278.3037	C14H18N2O4	PI3K	0.80	0.13	1.02	0.05
ZINC02835847	176.1672	C7H12O5	CYS path	0.90	0.14	1.04	0.05
ZINC02842688	294.081	C11H6BrN2O3	E3 ligase	0.84	0.03	0.92	0.34
ZINC02847150	277.239	C9H5N6O3S	PI3K	0.85	0.12	0.96	0.05
ZINC02860901	337.399	C17H15N5OS	HIS path	1.00	0.09	0.95	0.03
ZINC02927752	221.322	C11H15N3S	Autophagy	0.97	0.05	1.16	0.09
ZINC03013275	266.637	C11H7ClN2O4	E3 ligase	1.49	0.22	0.56	0.41
ZINC03040526	266.293	C13H18N2O4	Proteasome I-lact-mg 132	0.97	0.01	0.84	0.14
ZINC03057914	307.3449	C15H21N3O4	PI3K	0.92	0.06	1.16	0.35
ZINC03058094	307.3449	C15H21N3O4	PI3K	1.05	0.11	0.92	0.02
ZINC03091240	261.2301	C13H11NO5	HIS path	1.00	0.00	0.86	0.05
ZINC03124980	174.239	C12H14O	FASII-PDIM metabolism	0.83	0.07	1.23	0.13
ZINC03126171	316.3565	C19H16N4O	HIS path	1.12	0.06	0.83	0.18
ZINC03131412	317.3811	C21H19NO2	E3 ligase	1.02	0.07	0.96	0.05
ZINC03138016	291.2232	C12H12F3NO4	TRP path	0.99	0.01	1.02	0.11
ZINC03163860	245.664	C12H8ClN3O	Calcineurin1	0.90	0.11	0.28	0.10
ZINC03176776	302.3896	C21H20NO	GLN path	1.06	0.06	1.02	0.03
ZINC03180608	250.466	C8H2Cl3NO2	AKT1	0.79	0.02	0.17	0.12
ZINC03194940	273.327	C16H19NO3	TRP path	1.53	0.04	0.98	0.06
ZINC03209151	293.3232	C16H15N5O	MeC	0.83	0.08	1.03	0.15
ZINC03222749	280.3211	C17H16N2O2	Cations channels	1.40	0.05	0.95	0.10

ZINC03252794	267.665	C12H10ClNO4	HIS path	1.17	0.11
ZINC03262894	347.26	C14H16Cl2N2O2S	Proteasome I-lact-mg 132	0.95	0.16
ZINC03273208	302.779	C15H11ClN2OS	TRP path	1.00	0.04
ZINC03304614	219.67	C11H10ClN3	Autophagy	1.07	0.03
ZINC03307107	278.37	C14H18N2O2S	Proteasome I-lact-mg 132	1.06	0.25
ZINC03313212	232.083	C10H9Cl2O2	CYS path	0.97	0.03
ZINC03320009	247.359	C13H17N3S	Autophagy	0.85	0.25
ZINC03320544	251.348	C12H17N3OS	Calcineurin1	1.08	0.04
ZINC03332188	332.444	C15H16N4OS2	HIS path	0.88	0.17
ZINC03342051	280.3163	C15H20O5	PI3K	1.00	0.10
ZINC03359532	331.3233	C16H17N3O5	GLN path	0.86	0.12
ZINC03423395	348.4613	C22H26N3O	GLN path	0.92	0.09
ZINC03434615	216.2789	C13H16N2O	AKT1	0.91	0.14
ZINC03506116	312.363	C18H20N2O3	PI3K	1.08	0.13
ZINC03563045	314.405	C16H18N4OS	HIS path	1.02	0.03
ZINC03593534	189.2536	C12H15NO	Proteasome inducers	0.92	0.24
ZINC03593769	216.3419	C15H22N	Proteasome inducers	0.95	0.12
ZINC03634637	281.1703	C11H4F3N4O2	E3 ligase	0.94	0.11
ZINC03848524	287.3153	C18H13N3O	E3 ligase	1.06	0.23
ZINC03850741	223.2268	C14H9NO2	AKT1	1.11	0.01
ZINC03861620	238.2381	C15H10O3	AKT1	0.99	0.06
ZINC03874029	223.2268	C14H9NO2	AKT1	1.07	0.05
ZINC03875850	224.2115	C14H8O3	AKT1	0.85	0.20
ZINC03877943	203.2802	C13H17NO	Proteasome inducers	1.53	0.75
ZINC03882000	210.36	C14H26O	TRP path	1.00	0.10
ZINC03886933	229.2346	C12H11N3O2	TRP path	1.41	0.24
ZINC03887436	247.2251	C12H10FN3O2	HIS path	1.09	0.05
ZINC03887547	219.091	C7H4Cl2N2S	AKT1	1.24	0.08
ZINC03888794	272.733	C14H13ClN4	PI3K	1.08	0.18
ZINC03888966	221.342	C11H11NS2	Autophagy	0.77	0.17
ZINC03896048	226.2026	C14H7FO2	AKT1	0.97	0.13
ZINC03896782	224.2115	C14H8O3	AKT1	0.97	0.08
ZINC04020029	275.73	C15H14ClNO2	TRP path	0.90	0.04
ZINC04026621	344.48	C21H30NO3	Proteasome inducers	0.83	0.05
ZINC04060816	255.315	C15H17N3O	Calcineurin1	1.27	0.18
ZINC04060817	269.3416	C16H19N3O	Calcineurin1	1.04	0.06
ZINC04069859	278.284	C12H10N2O4S	HIS path	0.92	0.05
ZINC04071096	328.386	C17H16N2O3S	PI3K	0.97	0.09

1.11	0.20
1.30	0.10
1.23	0.07
0.98	0.04
0.92	0.15
1.27	0.23
1.14	0.04
0.97	0.02
1.05	0.05
0.92	0.07
0.99	0.05
0.97	0.12
1.19	0.28
1.02	0.13
0.97	0.10
1.15	0.21
1.02	0.13
1.07	0.07
0.81	0.17
0.78	0.11
0.84	0.03
0.98	0.06
0.99	0.03
0.90	0.18
0.99	0.05
0.90	0.07
1.04	0.05
0.78	0.13
1.16	0.34
0.98	0.07
0.90	0.12
0.71	0.14
1.38	0.16
0.93	0.05
0.50	0.15
0.41	0.23
0.97	0.02
0.96	0.11

ZINC04073909	340.4559	$C_{22}H_{28}O_3$	ATPase	1.10	0.21	0.94	0.09
ZINC04081983	336.3413	$C_{19}H_{16}N_2O_4$	ATPase	0.96	0.10	1.15	0.17
ZINC04088117	236.2222	$C_{15}H_8O_3$	AKT1	1.00	0.19	0.68	0.28
ZINC04099252	306.2345	$C_{13}H_{13}F_3O_5$	TRP path	0.96	0.09	0.84	0.19
ZINC04134394	291.163	$C_{14}H_{13}BrNO$	Autophagy	1.06	0.15	1.07	0.05
ZINC04166548	248.3837	$C_{16}H_{26}NO$	Proteasome inducers	1.03	0.08	1.10	0.16
ZINC04176923	333.385	$C_{14}H_{11}N_3O_3S_2$	HIS path	0.91	0.11	1.06	0.19
ZINC04180784	296.3238	$C_{16}H_{16}N_4O_2$	Proteasome inducers	0.89	0.05	0.98	0.12
ZINC04196124	306.357	$C_{15}H_{16}NO_4S$	CYS path	0.82	0.17	0.96	0.02
ZINC04196396	309.2186	$C_{11}H_{12}F_3N_2O_5$	Proteasome I-lact-mg 132	0.96	0.12	0.92	0.06
ZINC04218572	268.3088	$C_{16}H_{15}FN_3$	ABC transporters	1.58	0.12	0.93	0.08
ZINC04218988	272.685	$C_{11}H_{13}ClN_2O_4$	PI3K	1.07	0.07	1.03	0.18
ZINC04237889	243.2331	$C_{14}H_{10}FNO_2$	AKT1	1.09	0.11	0.82	0.18
ZINC04263292	256.076	$C_9H_8BrN_2O_2$	Autophagy	0.95	0.11	0.91	0.16
ZINC04281560	289.2021	$C_{11}H_6FN_6O_3$	PI3K	0.98	0.11	1.13	0.17
ZINC04298600	175.2038	$C_{11}H_{11}O_2$	CYS path	0.94	0.07	1.03	0.07
ZINC04301575	255.1	$C_{11}H_8Cl_2N_2O$	Proteasome I-lact-mg 132	1.00	0.16	1.03	0.03
ZINC04312213	258.2759	$C_{13}H_{14}N_4O_2$	MeC	1.19	0.34	0.89	0.21
ZINC04331436	287.3535	$C_{17}H_{21}NO_3$	TRP path	1.04	0.09	1.01	0.04
ZINC04360390	237.2533	$C_{15}H_{11}NO_2$	AKT1	1.12	0.15	0.98	0.09
ZINC04366006	315.3917	$C_{20}H_{19}N_4$	E3 ligase	0.89	0.15	0.13	0.18
ZINC04366134	329.4182	$C_{21}H_{21}N_4$	E3 ligase	0.36	0.04	0.59	0.30
ZINC04366461	224.2133	$C_{10}H_{12}N_2O_4$	TRP path	0.92	0.09	0.97	0.06
ZINC04374916	300.402	$C_{16}H_{24}N_6$	Calcineurin1	1.03	0.13	0.63	0.35
ZINC04375012	286.3754	$C_{15}H_{22}N_6$	Calcineurin1	1.05	0.09	0.96	0.04
ZINC04419464	239.3123	$C_{16}H_{17}NO$	Proteasome I-lact-mg 132	0.94	0.15	0.87	0.07
ZINC04427624	272.2776	$C_{14}H_{13}FN_4O$	MeC	0.75	0.10	1.03	0.10
ZINC04451200	301.244	$C_{11}H_6F_3N_3O_2S$	E3 ligase	0.91	0.07	1.07	0.19
ZINC04453439	288.3681	$C_{15}H_{22}N_5O$	Proteasome inducers	0.91	0.06	1.06	0.01
ZINC04476977	337.3294	$C_{18}H_{15}N_3O_4$	PI3K	1.01	0.05	0.93	0.03
ZINC04488253	331.3399	$C_{17}H_{17}F_2N_4O$	E3 ligase	0.78	0.07	0.85	0.04
ZINC04502525	200.2564	$C_{13}H_{14}NO$	Proteasome inducers	1.30	0.26	0.81	0.02
ZINC04507616	345.37	$C_{17}H_{15}NO_5S$	PI3K	0.80	0.04	1.03	0.10
ZINC04522397	271.3126	$C_{19}H_{13}NO$	E3 ligase	0.96	0.12	0.98	0.11
ZINC04536395	238.455	$C_7H_2Cl_3NO_2$	AKT1	0.33	0.06	0.21	0.17
ZINC04555680	342.3492	$C_{17}H_{18}N_4O_4$	ATPase	2.03	0.76	0.80	0.08
ZINC04571980	309.3591	$C_{19}H_{19}NO_3$	GLN path	0.82	0.10	1.08	0.11
ZINC04579056	294.349	$C_{12}H_{10}N_2O_3S_2$	HIS path	0.85	0.18	1.17	0.04

ZINC04579126	322.403	C14H14N2O3S2	HIS path	0.80	0.04		1.05	0.11
ZINC04588166	340.33	C18H16N2O5	PI3K	0.96	0.13		1.09	0.15
ZINC04595763	281.374	C13H19N3O2S	Calcineurin1	1.00	0.09		0.93	0.17
ZINC04610678	336.878	C22H23ClN	E3 ligase	1.18	0.02		0.88	0.28
ZINC04610887	276.291	C12H10N3O3S	CYS path	0.93	0.04		1.17	0.28
ZINC04620608	232.2304	C10H16O6	CYS path	0.82	0.05		0.91	0.07
ZINC04622505	298.3413	C19H14N4	ABC transporters	1.21	0.01		0.72	0.08
ZINC04626240	252.356	C11H12N2OS2	E3 ligase	1.05	0.02		1.05	0.10
ZINC04626243	266.382	C12H14N2OS2	E3 ligase	0.93	0.06		1.18	0.13
ZINC04626506	297.371	C17H15NO2S	CD36	1.00	0.03		1.30	0.23
ZINC04631579	274.3415	C14H20N5O	Proteasome inducers	0.91	0.00		0.88	0.09
ZINC04638824	244.289	C14H16N2O2	HIS path	0.88	0.09		1.02	0.09
ZINC04641406	296.3205	C17H16N2O3	ATPase	1.44	0.07		0.77	0.06
ZINC04646399	288.365	C15H16N2O2S	PI3K	1.07	0.14		1.04	0.13
ZINC04648755	220.2643	C13H16O3	FASII-PDIM metabolism	1.07	0.04		1.14	0.06
ZINC04648800	207.994	C7H2Cl2FO2	CYS path	0.95	0.10		0.88	0.15
ZINC04688380	299.3691	C20H17N3	GLN path	1.00	0.05		1.04	0.13
ZINC04692925	339.342	C19H17NO5	PI3K	0.77	0.02		0.99	0.08
ZINC04705780	305.3754	C15H23N5O2	Calcineurin1	1.00	0.02		0.87	0.21
ZINC04707313	258.2792	C12H14N6O	Cations channels	0.96	0.08		1.03	0.22
ZINC04741108	334.367	C16H16NO5S	CYS path	0.87	0.15		1.01	0.02
ZINC04742603	334.738	C13H7ClN4O3S	HIS path	0.80	0.09		1.02	0.09
ZINC04744175	298.3795	C18H22N2O2	Proteasome inducers	0.80	0.08		0.94	0.02
ZINC04748363	154.2493	C10H18O	TRP path	0.72	0.09		1.00	0.13
ZINC04748410	251.2369	C15H9NO3	AKT1	0.93	0.14		1.01	0.12
ZINC04748489	256.2997	C15H16N2O2	Cations channels	0.86	0.05		0.91	0.09
ZINC04754391	282.09	C11H8BrNO3	CD36	0.81	0.09		1.03	0.15
ZINC04763958	243.3672	C16H23N2	Proteasome inducers	0.97	0.07		0.99	0.08
ZINC04779402	294.3214	C15H19FN2O3	TRP path	0.82	0.03		1.01	0.11
ZINC04792462	276.3523	C19H18NO	Autophagy	1.00	0.03		0.82	0.08
ZINC04795898	328.342	C16H12N2O4S	HIS path	0.83	0.06		0.95	0.10
ZINC04824633	238.2414	C14H10N2O2	AKT1	0.95	0.13		1.06	0.14
ZINC04829851	210.3126	C13H22O2	ATPase	0.98	0.02		1.06	0.09
ZINC04831504	252.2631	C13H16O5	ATPase	0.96	0.10		0.86	0.05
ZINC04833129	288.2555	C14H12N2O5	HIS path	0.97	0.08		1.04	0.09
ZINC04859528	297.2655	C15H11N3O4	TRP path	1.26	0.13		0.80	0.14
ZINC04863124	260.353	C11H20N2O3S	Proteasome I-lact-mg 132	0.90	0.09		1.02	0.08
ZINC04866196	340.32	C15H11F3N2O2S	Proteasome I-lact-mg 132	0.76	0.04		0.98	0.08

ZINC04867578	286.349	C15H14N2O2S	TRP path	0.99	0.15	0.83	0.08
ZINC04891102	267.2793	C16H13NO3	AKT1	0.96	0.05	0.73	0.15
ZINC04896295	218.2469	C10H18O5	Proteasome I-lact-mg 132	0.96	0.01	1.03	0.08
ZINC04913274	302.732	C16H13ClNO3	CYS path	0.88	0.15	1.01	0.03
ZINC04918167	206.2808	C13H18O2	Proteasome I-lact-mg 132	0.60	0.17	1.06	0.10
ZINC04927497	292.678	C12H9ClN4O3	HIS path	1.21	0.16	0.91	0.14
ZINC04940217	338.788	C19H15ClN2O2	Lip	0.98	0.08	1.09	0.16
ZINC04940272	340.3235	C19H14F2N2O2	Lip	0.98	0.07	0.99	0.11
ZINC04940362	318.3691	C20H18N2O2	Lip	1.14	0.13	1.06	0.10
ZINC04940369	336.3596	C20H17FN2O2	Lip	1.11	0.10	1.14	0.15
ZINC04941201	320.3404	C16H20N2O5	HIS path	0.96	0.15	0.84	0.05
ZINC04946864	280.2814	C15H12N4O2	E3 ligase	1.13	0.08	0.95	0.12
ZINC04949688	344.2394	C15H11F3O6	ATPase	0.98	0.15	0.86	0.03
ZINC04962723	280.3178	C18H16O3	E3 ligase	0.89	0.10	1.18	0.25
ZINC04983843	237.3379	C14H23NO2	Proteasome I-lact-mg 132	0.96	0.10	1.08	0.23
ZINC04985087	252.697	C12H13ClN2O2	TRP path	1.22	0.25	0.99	0.07
ZINC04987648	266.319	C10H8N3O2S2	E3 ligase	1.26	0.17	0.89	0.24
ZINC04988240	263.3123	C14H19N2O3	Autophagy	0.88	0.05	1.11	0.13
ZINC04991189	264.323	C11H8N2O2S2	Cations channels	0.91	0.03	0.92	0.16
ZINC04991217	258.296	C13H10N2O2S	Cations channels	0.92	0.03	0.90	0.12
ZINC04992651	195.2582	C11H17NO2	TRP path	0.91	0.03	1.15	0.25
ZINC05001017	233.333	C12H15N3S	Autophagy	1.05	0.12	1.03	0.09
ZINC05013158	340.3333	C17H16N4O4	HIS path	0.99	0.10	0.72	0.11
ZINC05029928	153.1983	C9H13O2	CYS path	0.82	0.07	1.04	0.09
ZINC05049463	337.4354	C21H25N2O2	Proteasome inducers	0.90	0.05	1.04	0.10
ZINC05049597	272.2561	C14H12N2O4	HIS path	1.11	0.10	1.00	0.06
ZINC05120095	259.633	C7H5ClF3NO2S	Cations channels	0.81	0.05	0.91	0.14
ZINC05152004	297.459	C15H27N3OS	HIS path	0.90	0.11	0.98	0.02
ZINC05209705	269.2177	C10H14F3NO4	Proteasome I-lact-mg 132	0.97	0.10	0.99	0.13
ZINC05218034	252.3077	C17H16O2	Lip	1.00	0.07	1.05	0.20
ZINC05219192	272.3422	C16H20N2O2	CD36	0.89	0.04	0.90	0.23
ZINC05223924	330.3982	C19H24NO4	Proteasome inducers	0.95	0.05	0.98	0.07
ZINC05224786	239.2262	C14H9NO3	AKT1	1.11	0.14	0.46	0.19
ZINC05266909	256.323	C14H12N2OS	E3 ligase	0.92	0.13	1.01	0.07
ZINC05268744	340.3235	C19H14F2N2O2	Lip	0.98	0.10	1.05	0.13
ZINC05270155	336.3596	C20H17FN2O2	Lip	0.98	0.06	1.13	0.13
ZINC05273977	328.3624	C18H20N2O4	Proteasome I-lact-mg 132	0.92	0.06	1.05	0.10
ZINC05277622	289.3495	C16H21N2O3	CYS path	0.74	0.24	0.93	0.08

ZINC05286502	216.2789	C13H16N2O	TRP path	0.93	0.02	0.83	0.07
ZINC05295363	288.732	C14H13ClN4O	MeC	0.98	0.06	0.86	0.12
ZINC05295368	288.732	C14H13ClN4O	MeC	0.97	0.06	0.86	0.08
ZINC05297481	333.183	C14H13BrN4O	MeC	0.90	0.06	1.17	0.17
ZINC05310967	193.2854	C12H19NO	TRP path	1.00	0.03	0.85	0.10
ZINC05331442	213.063	C9H6Cl2N2	Proteasome I-lact-mg 132	1.13	0.15	0.71	0.24
ZINC05331770	273.3071	C15H17N2O3	CYS path	0.77	0.05	0.87	0.14
ZINC05332174	217.3068	C14H19NO	Proteasome I-lact-mg 132	1.00	0.06	0.91	0.10
ZINC05353862	328.3657	C17H20N4O3	MeC	1.09	0.20	1.00	0.11
ZINC05368627	320.813	C6H4Cl6O2	ATPase	1.39	0.05	0.76	0.09
ZINC05369382	187.2145	C12H11O2	CYS path	1.22	0.13	0.82	0.10
ZINC05370009	316.3731	C21H18NO2	E3 ligase	0.95	0.05	0.78	0.12
ZINC05377989	282.382	C12H14N2O2S2	ATPase	0.91	0.05	0.91	0.14
ZINC05378575	216.2789	C13H16N2O	Autophagy	0.91	0.06	0.88	0.11
ZINC05380703	163.2395	C10H15N2	Proteasome I-lact-mg 132	0.91	0.04	0.92	0.09
ZINC05381035	297.35	C16H12FN3S	GLN path	0.99	0.13	0.83	0.07
ZINC05385585	218.2948	C13H18N2O	TRP path	0.96	0.06	0.87	0.06
ZINC05386873	275.717	C12H12ClN6	Cations channels	2.42	0.33	0.91	0.17
ZINC05387150	176.2581	C11H16N2	Proteasome I-lact-mg 132	0.97	0.13	1.01	0.05
ZINC05389739	288.282	C12H8N4O3S	HIS path	1.01	0.05	0.94	0.04
ZINC05394129	194.2319	C13H10N2	AKT1	0.83	0.10	1.10	0.16
ZINC05403161	282.3403	C16H18N4O	MeC	0.92	0.06	0.97	0.10
ZINC05403212	328.3657	C17H20N4O3	MeC	0.89	0.03	1.01	0.03
ZINC05415246	348.458	C23H26NO2	E3 ligase	1.03	0.14	1.00	0.19
ZINC05416357	294.3462	C15H22N2O4	GLN path	1.01	0.06	0.87	0.06
ZINC05464628	226.355	C14H26O2	Proteasome I-lact-mg 132	0.99	0.10	0.92	0.11
ZINC05478569	250.707	C10H7ClN4S	Calcineurin1	0.66	0.19	0.77	0.12
ZINC05479619	288.3482	C14H20N6O	Calcineurin1	1.00	0.12	1.22	0.34
ZINC05479620	288.3482	C14H20N6O	Calcineurin1	1.00	0.16	1.14	0.24
ZINC05487917	236.2653	C16H12O2	Lip	1.10	0.21	1.25	0.12
ZINC05510798	211.476	C6H5Cl3N2	Proteasome I-lact-mg 132	1.08	0.02	1.05	0.12
ZINC05514187	253.2512	C12H15NO5	PI3K	0.99	0.04	1.21	0.15
ZINC05515149	342.704	C14H10ClF3N4O	E3 ligase	1.00	0.13	0.99	0.11
ZINC05566693	297.077	C8H10Cl2F4N2O	TRP path	0.94	0.01	0.99	0.06
ZINC05567999	294.71	C14H13ClNO4	TRP path	0.90	0.11	0.90	0.04
ZINC05569042	287.2277	C13H9N3O5	HIS path	0.96	0.06	0.64	0.21
ZINC05572865	260.2421	C14H12O5	AKT1	0.88	0.22	0.82	0.38
ZINC05586339	325.4081	C18H23N5O	E3 ligase	1.15	0.06	0.97	0.19

ZINC05603592	269.3383	C17H19NO2	Proteasome I-lact-mg 132	0.98	0.08	1.00	0.02
ZINC05603732	258.3123	C16H18O3	ATPase	1.14	0.06	1.05	0.11
ZINC05603746	348.73	C14H15ClF2N2O4	Proteasome I-lact-mg 132	0.89	0.12	1.02	0.08
ZINC05604078	212.2423	C11H16O4	ATPase	0.94	0.05	0.83	0.15
ZINC05615643	278.2607	C13H14N2O5	PI3K	0.73	0.08	1.18	0.16
ZINC05621489	226.2592	C10H19F3NO	TRP path	0.95	0.05	1.02	0.12
ZINC05625821	207.2722	C11H17N3O	Calcineurin1	0.85	0.07	0.93	0.17
ZINC05697972	283.3251	C16H17N3O2	Calcineurin1	1.00	0.03	0.84	0.13
ZINC05699716	198.2604	C14H14O	Proteasome inducers	1.20	0.17	0.98	0.19
ZINC05717049	308.2668	C13H14N3O6	PI3K	1.00	0.15	0.83	0.15
ZINC05720306	302.2821	C15H14N2O5	HIS path	0.98	0.08	0.98	0.06
ZINC05732949	316.4146	C19H26NO3	Proteasome inducers	0.95	0.08	0.93	0.08
ZINC05732955	316.4146	C19H26NO3	Proteasome inducers	0.77	0.02	1.03	0.14
ZINC05738750	325.2858	C15H14F3N3O2	PI3K	1.01	0.05	1.30	0.32
ZINC05793393	268.701	C14H9ClN4	Calcineurin1	1.00	0.03	0.91	0.12
ZINC05814054	335.421	C16H21N3O3S	HIS path	0.98	0.02	1.11	0.04
ZINC05830242	226.337	C7H16NO3S2	Proteasome I-lact-mg 132	0.89	0.02	0.96	0.04
ZINC05879226	345.4	C21H19N3O2	E3 ligase	1.02	0.07	0.98	0.05
ZINC05891973	189.2984	C9H23N3O	Porins	1.01	0.15	1.00	0.10
ZINC05938203	259.37	C14H17N3S	Autophagy	1.04	0.19	1.07	0.06
ZINC05939079	250.2952	C16H14N2O	CD36	0.97	0.07	0.90	0.10
ZINC05961188	244.12	C10H11Cl2N3	Calcineurin1	1.04	0.05	0.40	0.26
ZINC05999601	251.2799	C16H13NO2	Lip	1.03	0.09	0.87	0.04
ZINC06069162	183.2291	C12H11N2	AKT1	1.06	0.14	1.02	0.10
ZINC06070238	212.2472	C13H12N2O	AKT1	1.10	0.09	1.06	0.04
ZINC06152172	338.4003	C20H22N2O3	GLN path	0.98	0.08	1.07	0.10
ZINC06203084	307.3034	C17H13N3O3	HIS path	0.97	0.04	1.01	0.09
ZINC06253039	234.271	C13H16NO3	CYS path	0.88	0.01	1.11	0.12
ZINC06292969	269.3	C10H13N4O3S	Cations channels	1.02	0.08	0.88	0.22
ZINC06348552	262.284	C12H10N2O3S	AKT1	0.90	0.03	0.93	0.14
ZINC06393516	341.4042	C19H23N3O3	PI3K	0.97	0.06	1.13	0.15
ZINC06401380	247.742	C10H14ClNO2S	Cations channels	1.07	0.11	0.93	0.05
ZINC06401383	288.579	C8H8Cl3NO2S	Cations channels	0.98	0.05	1.06	0.19
ZINC06509614	188.2258	C11H12N2O	AKT1	1.08	0.04	1.00	0.13
ZINC06529083	270.2866	C14H14N4O2	TRP path	1.01	0.07	0.74	0.14
ZINC06543951	345.459	C18H23N3O2S	HIS path	0.90	0.03	1.07	0.02
ZINC06549877	333.3407	C19H15N3O3	ABC transporters	1.07	0.08	0.90	0.06
ZINC06556279	253.2958	C16H15NO2	Proteasome inducers	0.87	0.02	0.81	0.11

ZINC06556809	253.2991	C15H15N3O	Calcineurin1	0.81	0.09	0.80	0.06
ZINC06562810	212.63	C10H9ClO3	Proteasome I-lact-mg 132	1.04	0.05	0.80	0.17
ZINC06588888	251.226	C9H8F3NO2S	Cations channels	0.98	0.01	0.96	0.10
ZINC06690930	223.2731	C14H13N3	Calcineurin1	0.85	0.15	1.12	0.20
ZINC06727051	228.651	C10H10ClFN2O	TRP path	0.84	0.12	0.96	0.11
ZINC06741366	300.589	C9H8Cl3NO2S	Cations channels	0.81	0.06	1.06	0.12
ZINC06742607	343.447	C17H21N5OS	HIS path	0.85	0.10	1.09	0.18
ZINC06742730	268.16	C9H11Cl2NO2S	Cations channels	1.03	0.05	1.01	0.05
ZINC06746182	267.715	C11H14ClN5O	Calcineurin1	0.95	0.04	0.95	0.12
ZINC06750015	258.337	C11H18N2O3S	Proteasome inducers	0.93	0.10	1.01	0.09
ZINC06759730	280.3244	C16H16N4O	Calcineurin1	0.94	0.05	1.03	0.10
ZINC06787309	280.3211	C17H16N2O2	Cations channels	1.17	0.06	0.96	0.11
ZINC06794427	251.265	C9H9N5O2S	PI3K	1.07	0.07	0.84	0.13
ZINC06818375	330.401	C17H18N2O3S	CD36	0.95	0.13	0.97	0.15
ZINC06852338	299.392	C12H21N5O2S	HIS path	0.87	0.18	1.24	0.09
ZINC06859231	206.241	C10H8NO2S	CYS path	0.82	0.10	1.08	0.05
ZINC06938279	343.4201	C19H25N3O3	GLN path	0.75	0.32	1.02	0.10
ZINC07099450	276.3324	C18H16N2O	FASII-PDIM metabolism	1.06	0.17	0.98	0.09
ZINC07113192	306.2824	C16H13F3N2O	Lip	1.21	0.04	0.94	0.10
ZINC07150368	344.3552	C19H18F2N2O2	GLN path	0.86	0.22	1.16	0.09
ZINC07217536	340.3731	C19H20N2O4	GLN path	0.84	0.11	0.92	0.05
ZINC07375448	265.252	C10H10F3NO2S	Cations channels	1.15	0.12	1.00	0.03
ZINC07382478	340.4162	C20H24N2O3	ABC transporters	0.98	0.10	1.02	0.10
ZINC07415970	307.454	C16H25N3OS	HIS path	1.05	0.10	0.85	0.04
ZINC07416075	307.454	C16H25N3OS	HIS path	1.00	0.11	1.08	0.14
ZINC07452535	309.3624	C18H19N3O2	GLN path	0.97	0.07	0.91	0.07
ZINC07570637	339.776	C18H14ClN3O2	GLN path	0.88	0.17	0.93	0.07
ZINC07573931	336.3596	C20H17FN2O2	GLN path	1.01	0.14	1.01	0.12
ZINC07636163	246.696	C12H11ClN4	Calcineurin1	0.61	0.58	1.00	0.13
ZINC07703263	293.305	C12H14F3NO2S	Cations channels	1.33	0.06	1.02	0.19
ZINC07744890	324.3439	C20H16F2NO	GLN path	1.08	0.05	0.88	0.16
ZINC07778471	335.444	C15H17N3O2S2	GLN path	0.96	0.06	1.04	0.07
ZINC07885975	264.3217	C17H16N2O	Lip	1.00	0.03	1.11	0.16
ZINC07885993	307.3895	C19H21N3O	Lip	0.98	0.11	0.95	0.13
ZINC07886015	294.3477	C18H18N2O2	Lip	1.12	0.18	1.05	0.13
ZINC07886335	322.4009	C20H22N2O2	Lip	1.42	0.04	0.93	0.14
ZINC07946439	260.2884	C14H16N2O3	PI3K	0.91	0.08	1.00	0.05
ZINC08113123	278.327	C13H14N2O3S	HIS path	0.88	0.12	0.88	0.05

ZINC08324450	335.426	C18H17N5S	GLN path	0.89	0.08	0.98	0.04
ZINC08325649	302.3682	C17H22N2O3	HIS path	1.11	0.05	0.99	0.03
ZINC08393128	258.299	C12H10N4OS	E3 ligase	1.01	0.18	0.89	0.08
ZINC08462925	176.278	C12H18N	FASII-PDIM metabolism	0.83	0.10	0.95	0.06
ZINC08536493	331.3662	C20H16FN4	ABC transporters	1.29	0.12	0.94	0.21
ZINC08578841	284.2469	C14H10N3O4	E3 ligase	1.01	0.08	1.02	0.11
ZINC08584475	271.338	C14H13N3OS	CD36	1.06	0.05	1.05	0.09
ZINC08586750	313.3693	C18H20FN3O	GLN path	0.86	0.03	1.11	0.08
ZINC08615381	284.3562	C16H20N4O	E3 ligase	1.13	0.27	1.04	0.09
ZINC08619391	321.541	C12H7Cl3O4	TRP path	1.12	0.25	0.90	0.16
ZINC08662085	301.3834	C17H23N3O2	ATPase	1.15	0.09	0.99	0.09
ZINC08673103	221.2539	C15H11NO	CD36	0.98	0.03	0.96	0.11
ZINC08673151	270.2203	C13H8N3O4	E3 ligase	0.95	0.05	0.97	0.10
ZINC08706421	348.422	C19H16N4OS	HIS path	0.83	0.14	0.90	0.06
ZINC08736282	296.3205	C17H16N2O3	CD36	0.89	0.06	0.96	0.04
ZINC08743020	339.279	C17H11N2O6	GLN path	0.76	0.16	0.96	0.13
ZINC08743431	341.318	C16H9N2O5S	GLN path	1.02	0.11	0.82	0.09
ZINC08744114	338.3572	C19H18N2O4	ATPase	1.03	0.01	0.82	0.15
ZINC08783696	290.384	C14H18N4OS	HIS path	0.93	0.07	0.94	0.03
ZINC08803963	296.3205	C17H16N2O3	CD36	0.93	0.07	1.12	0.07
ZINC08803966	296.3205	C17H16N2O3	CD36	0.94	0.06	1.02	0.08
ZINC08816935	298.3397	C16H18N4O2	MeC	0.92	0.06	1.00	0.16
ZINC08816950	314.3391	C16H18N4O3	MeC	1.06	0.15	1.00	0.06
ZINC08828995	253.362	C16H15NS	Lip	1.09	0.13	1.05	0.23
ZINC08861086	265.2634	C16H11NO3	CD36	0.92	0.02	0.97	0.14
ZINC08873612	286.759	C15H15ClN4	Calcineurin1	0.96	0.05	1.03	0.14
ZINC08877550	258.294	C10H14N2O4S	CYS path	1.02	0.02	0.93	0.08
ZINC08903697	270.2866	C14H14N4O2	MeC	1.04	0.11	0.81	0.14
ZINC08918980	310.3471	C18H18N2O3	CD36	1.05	0.02	1.04	0.09
ZINC08939401	296.3205	C17H16N2O3	CD36	1.02	0.07	0.91	0.02
ZINC08973079	340.33	C18H16N2O5	ATPase	1.60	0.32	0.79	0.08
ZINC08982828	270.133	C8H9Cl2NO3S	Cations channels	1.71	0.17	0.92	0.22
ZINC08983659	210.488	C7H6Cl3N	Calcineurin1	1.02	0.07	0.91	0.03
ZINC08983758	203.186	C8H5F2O2S	CYS path	1.00	0.03	1.08	0.06
ZINC09007754	269.3018	C14H15N5O	MeC	0.79	0.12	1.14	0.05
ZINC09007974	324.377	C18H20N4O2	MeC	1.35	0.18	0.71	0.08
ZINC09041977	298.3397	C16H18N4O2	MeC	1.01	0.08	0.97	0.03
ZINC09061925	268.16	C9H11Cl2NO2S	Cations channels	1.04	0.09	0.94	0.11

ZINC09089920	282.2741	C15H12N3O3	E3 ligase	0.95	0.09	0.92	0.12
ZINC09142721	302.33	C18H14N4O	ATPase	0.94	0.13	1.07	0.16
ZINC09190342	321.33	C18H15N3O3	HIS path	0.85	0.06	1.05	0.07
ZINC09202654	255.661	C9H10ClN5O2	Calcineurin1	0.96	0.06	0.95	0.15
ZINC09233160	298.3397	C16H18N4O2	MeC	1.01	0.06	1.10	0.07
ZINC09271185	270.2866	C14H14N4O2	MeC	1.00	0.03	0.93	0.13
ZINC09275714	254.2872	C14H14N4O	MeC	1.04	0.09	1.03	0.11
ZINC09303426	272.3422	C16H20N2O2	CD36	0.86	0.09	1.03	0.08
ZINC09306182	301.57	C10H10BrClN4	Calcineurin1	1.05	0.15	0.41	0.06
ZINC09423548	306.3617	C18H18N4O	PI3K	0.93	0.04	1.02	0.06
ZINC09450710	318.303	C15H15FN4O3	HIS path	0.98	0.07	1.10	0.17
ZINC09530919	332.397	C16H18N3O3S	CYS path	0.92	0.18	0.79	0.14
ZINC09533404	275.326	C13H13N3O2S	Autophagy	0.98	0.11	0.97	0.02
ZINC09695761	270.122	C11H12BrNO2	Autophagy	1.26	0.22	1.13	0.05
ZINC09695795	258.112	C10H12BrNO2	Autophagy	1.04	0.07	0.96	0.12
ZINC09711183	302.391	C16H18N2O2S	FASII-PDIM metabolism	1.14	0.11	1.28	0.32
ZINC09721393	314.362	C15H14N4O2S	PI3K	0.81	0.25	1.12	0.07
ZINC09896899	310.413	C18H18N2OS	FASII-PDIM metabolism	1.09	0.05	1.01	0.13
ZINC09949563	298.2951	C12H18N4O5	Proteasome I-lact-mg 132	1.01	0.07	1.08	0.13
ZINC09952766	296.3205	C17H16N2O3	CD36	1.05	0.07	0.96	0.08
ZINC09961270	294.3908	C19H22N2O	Lip	1.02	0.03	1.19	0.22
ZINC10030690	314.4252	C18H26N4O	Calcineurin1	1.09	0.03	0.97	0.12
ZINC10053396	349.443	C18H22FN2O2S	Autophagy	0.89	0.14	1.17	0.03
ZINC10187848	323.3211	C18H14FN3O2	GLN path	0.94	0.10	0.96	0.12
ZINC10187894	319.3572	C19H17N3O2	GLN path	1.00	0.10	0.89	0.05
ZINC10220537	340.759	C15H17ClN2O5	HIS path	0.98	0.14	0.87	0.07
ZINC10335826	285.341	C16H19N3O2	HIS path	0.96	0.05	0.97	0.15
ZINC10509267	289.3048	C15H16FN3O2	HIS path	0.88	0.08	0.83	0.23
ZINC10860306	266.508	C9H6Cl3NO2	AKT1	0.89	0.16	1.19	0.20
ZINC10922095	310.413	C18H18N2OS	FASII-PDIM metabolism	0.98	0.06	1.17	0.14
ZINC10923707	342.412	C18H18N2O3S	FASII-PDIM metabolism	1.12	0.08	0.94	0.15
ZINC10924807	338.423	C19H18N2O2S	FASII-PDIM metabolism	1.10	0.02	0.93	0.03
ZINC11304625	321.438	C16H23N3O2S	HIS path	0.78	0.22	0.91	0.04
ZINC11566232	328.212	C11H15Cl2NO4S	ATPase	1.04	0.10	0.97	0.09
ZINC11631047	191.2463	C12H15O2	CYS path	0.96	0.11	0.87	0.10
ZINC11631048	205.2729	C13H17O2	CYS path	1.09	0.13	0.90	0.43
ZINC11750559	344.428	C18H20N2O3S	Lip	1.14	0.05	1.00	0.13
ZINC11754146	348.3984	C20H20N4O2	GLN path	1.01	0.20	0.96	0.08

ZINC11813584	349.815	C16H20ClN5O2	Proteasome I-lact-mg 132	0.79	0.05	0.93	0.07
ZINC11818405	331.4078	C18H24FN4O	GLN path	1.10	0.13	0.98	0.04
ZINC11847627	274.3349	C16H20NO3	CYS path	1.28	0.27	0.93	0.06
ZINC11865129	268.2641	C16H12O4	Autophagy	1.00	0.04	1.03	0.08
ZINC11874364	320.513	C19H30NOS	Proteasome I-lact-mg 132	0.93	0.06	1.14	0.05
ZINC11885310	184.257	C13H14N	Proteasome inducers	1.02	0.14	0.81	0.12
ZINC11899274	311.3749	C19H21NO3	Lip	1.03	0.14	0.90	0.10
ZINC11899821	300.3275	C17H17FN2O2	Lip	1.00	0.20	0.95	0.10
ZINC11919811	262.263	C11H7FN4OS	E3 ligase	0.96	0.05	0.86	0.06
ZINC11990412	254.2375	C15H10O4	Autophagy	1.14	0.10	0.36	0.29
ZINC12146595	340.4	C17H16N4O2S	Proteasome I-lact-mg 132	0.90	0.03	1.18	0.22
ZINC12146636	330.3816	C17H22N4O3	Proteasome I-lact-mg 132	1.05	0.16	0.92	0.20
ZINC12181217	304.367	C14H16N4O2S	Proteasome I-lact-mg 132	1.17	0.06	1.03	0.13
ZINC12181273	346.447	C17H22N4O2S	Proteasome I-lact-mg 132	1.18	0.06	1.06	0.10
ZINC12182870	302.3284	C15H18N4O3	Proteasome I-lact-mg 132	1.12	0.11	1.14	0.26
ZINC12215833	328.452	C17H16N2OS2	FASII-PDIM metabolism	1.34	0.05	0.89	0.12
ZINC12216622	308.419	C14H16N2O2S2	FASII-PDIM metabolism	0.99	0.09	0.94	0.07
ZINC12297686	320.3586	C17H21FN2O3	GLN path	0.94	0.02	1.02	0.04
ZINC12321303	166.217	C10H14O2	Proteasome I-lact-mg 132	0.96	0.03	0.99	0.11
ZINC12324403	320.385	C20H20N2O2	FASII-PDIM metabolism	1.03	0.01	1.00	0.10
ZINC12324747	344.4049	C19H24N2O4	FASII-PDIM metabolism	0.88	0.07	1.01	0.14
ZINC12343803	280.171	C10H11Cl2NO2S	Cations channels	0.94	0.04	1.30	0.17
ZINC12359287	212.554	C7H4ClF3O2	Proteasome I-lact-mg 132	1.02	0.13	0.92	0.08
ZINC12360025	224.054	C9H6BrNO	Autophagy	1.28	0.11	0.67	0.20
ZINC12375300	240.2341	C14H10NO3	TRP path	0.79	0.09	0.91	0.06
ZINC12376632	346.3825	C20H18N4O2	GLN path	0.80	0.07	1.10	0.04
ZINC12381611	304.341	C16H20N2O4	PI3K	1.03	0.03	1.00	0.06
ZINC12382296	296.213	C11H15Cl2NO2S	Cations channels	0.83	0.09	1.03	0.05
ZINC12382299	261.768	C11H16ClNO2S	Cations channels	0.82	0.13	0.95	0.07
ZINC12382642	282.187	C10H13Cl2NO2S	Cations channels	0.77	0.08	0.94	0.12
ZINC12406878	339.173	C15H12Cl2N2O3	PI3K	1.08	0.13	0.95	0.12
ZINC12417527	167.2249	C10H15O2	CYS path	0.65	0.20	0.94	0.09
ZINC12430509	336.813	C17H21ClN2O3	GLN path	0.97	0.06	0.97	0.04
ZINC12478058	314.3988	C19H24NO3	Proteasome inducers	0.88	0.12	1.07	0.09
ZINC12504647	340.328	C17H9FN2O3S	HIS path	0.79	0.11	1.10	0.05
ZINC12505623	210.614	C10H7ClO3	Proteasome I-lact-mg 132	0.98	0.39	1.19	0.03
ZINC12506052	214.048	C9H5Cl2NO	CD36	0.79	0.06	0.96	0.05
ZINC12523263	320.385	C20H20N2O2	Lip	1.18	0.06	0.81	0.05

ZINC12552457	331.4109	C21H21N3O	PI3K	1.06	0.03	0.94	0.11
ZINC12559775	248.241	C9H6N5O2S	TRP path	0.83	0.13	1.05	0.18
ZINC12575487	349.453	C19H19N5S	ATPase	1.09	0.05	0.88	0.09
ZINC12578258	343.3839	C18H24F3NO2	Proteasome I-lact-mg 132	1.44	0.26	0.88	0.05
ZINC12580863	296.3868	C18H22N3O	Proteasome I-lact-mg 132	1.06	0.11	0.91	0.01
ZINC12590690	330.42	C18H19FN2OS	FASII-PDIM metabolism	1.15	0.06	0.86	0.09
ZINC12604496	331.3712	C19H17N5O	HIS path	0.90	0.09	0.90	0.02
ZINC12618949	290.3144	C15H18N2O4	E3 ligase	0.86	0.15	1.01	0.04
ZINC12627228	328.3672	C20H16N4O	HIS path	0.87	0.11	0.86	0.04
ZINC12704026	297.3914	C19H23NO2	GLN path	2.00	0.65	1.13	0.12
ZINC12726078	220.2478	C11H14N3O2	CYS path	1.02	0.01	0.88	0.09
ZINC12749721	312.3812	C19H21FN2O	ABC transporters	1.01	0.20	0.93	0.13
ZINC12771242	332.417	C17H20N2O3S	PI3K	1.22	0.14	0.96	0.10
ZINC12787622	290.404	C14H14N2OS2	FASII-PDIM metabolism	1.00	0.03	1.05	0.14
ZINC12808196	326.777	C18H15ClN2O2	CD36	1.03	0.01	1.07	0.22
ZINC12902715	314.359	C16H14N2O3S	E3 ligase	0.85	0.23	1.12	0.07
ZINC12911732	302.3715	C16H22N4O2	Calcineurin1	1.01	0.07	1.19	0.22
ZINC12919118	287.377	C16H17NO2S	FASII-PDIM metabolism	1.22	0.03	0.83	0.14
ZINC12919598	330.4677	C19H30N4O	Calcineurin1	0.97	0.01	0.99	0.08
ZINC12933380	192.645	C10H9ClN2	AKT1	1.03	0.05	1.09	0.26
ZINC12940181	259.3052	C17H13N3	AKT1	1.30	0.14	0.90	0.33
ZINC12972099	331.429	C18H21NO3S	FASII-PDIM metabolism	1.13	0.12	1.19	0.26
ZINC12972807	299.3675	C17H21N3O2	ATPase	0.94	0.06	0.93	0.05
ZINC12993734	325.4048	C19H23N3O2	GLN path	0.92	0.10	0.89	0.03
ZINC13004621	302.4145	C17H26N4O	Calcineurin1	1.04	0.06	0.93	0.01
ZINC13005325	340.3731	C19H20N2O4	CD36	1.06	0.02	0.88	0.10
ZINC13022844	342.458	C18H22N4OS	Calcineurin1	1.13	0.13	0.91	0.07
ZINC13023074	296.3205	C17H16N2O3	CD36	1.11	0.07	1.33	0.44
ZINC13114384	308.277	C11H11F3N2O3S	Proteasome I-lact-mg 132	0.94	0.03	1.03	0.08
ZINC13125630	278.3053	C17H14N2O2	ABC transporters	1.06	0.13	1.10	0.21
ZINC13135348	349.494	C15H15N3OS3	GLN path	0.89	0.07	0.89	0.03
ZINC13137661	327.3959	C19H22FN3O	ABC transporters	0.79	0.09	0.81	0.16
ZINC13170396	276.2829	C15H15FNO3	CYS path	1.03	0.28	1.21	0.05
ZINC13212469	289.3793	C14H23N7	Cations channels	1.08	0.06	1.01	0.28
ZINC13283456	278.737	C13H15ClN4O	Cations channels	0.96	0.07	0.93	0.24
ZINC13284722	303.547	C9H11Cl3NO4	Proteasome I-lact-mg 132	0.82	0.01	0.92	0.05
ZINC13284927	278.737	C13H15ClN4O	Cations channels	0.94	0.08	1.04	0.15
ZINC13284972	313.182	C13H14Cl2N4O	Cations channels	0.88	0.05	0.93	0.16

ZINC13395297	299.371	C14H9N3OS2	E3 ligase	0.82	0.08	1.07	0.20
ZINC13439996	282.3138	C17H16NO3	CYS path	0.91	0.04	1.17	0.06
ZINC13469949	316.3716	C18H22NO4	TRP path	1.01	0.08	1.01	0.05
ZINC13495675	190.3046	C13H20N	FASII-PDIM metabolism	1.15	0.12	0.96	0.02
ZINC13522232	335.3533	C20H17NO4	CD36	1.04	0.02	0.98	0.12
ZINC13556356	236.2719	C14H12N4	ABC transporters	1.01	0.11	0.89	0.15
ZINC13573701	265.2634	C16H11NO3	Autophagy	1.00	0.09	1.14	0.15
ZINC13631900	277.791	C11H18ClN2O2S	Cations channels	0.81	0.12	1.02	0.17
ZINC13635946	274.341	C13H14N4OS	MeC	0.99	0.06	0.99	0.09
ZINC13647306	312.236	C11H17Cl2N2O2S	Cations channels	0.76	0.13	1.02	0.20
ZINC13689006	299.3245	C16H17N3O3	Proteasome I-lact-mg 132	0.84	0.06	1.03	0.12
ZINC13719676	341.4473	C20H27N3O2	ATPase	0.99	0.11	0.95	0.11
ZINC13725782	287.38	C15H17N3OS	CD36	0.89	0.04	0.95	0.14
ZINC13725877	287.38	C15H17N3OS	CD36	0.91	0.05	0.88	0.17
ZINC13727007	305.395	C15H19N3O2S	CD36	0.90	0.08	0.90	0.09
ZINC13943902	285.3	C10H13N4O4S	Cations channels	1.01	0.08	1.05	0.11
ZINC14291486	247.742	C10H14ClNO2S	Cations channels	0.83	0.10	1.06	0.07
ZINC14559906	295.3358	C17H17N3O2	GLN path	0.94	0.04	1.05	0.04
ZINC14953906	341.828	C17H23ClFN2O2	TRP path	1.02	0.09	1.03	0.04
ZINC15065999	331.3415	C17H18FN3O3	GLN path	0.89	0.04	1.00	0.05
ZINC15071801	345.4343	C19H26FN4O	GLN path	0.78	0.10	0.96	0.13
ZINC15162202	312.041	C8H10Cl4O2S	CYS path	0.90	0.06	0.88	0.16
ZINC15303951	297.137	C13H10Cl2N2O2	CD36	1.09	0.12	1.04	0.15
ZINC15306899	338.4433	C21H26N2O2	Autophagy	1.07	0.06	0.96	0.06
ZINC15306918	289.3926	C17H25N2O2	Autophagy	0.73	0.12	0.96	0.23
ZINC15306941	298.421	C15H24NO3S	Autophagy	0.87	0.13	1.11	0.02
ZINC15440761	176.2548	C12H16O	FASII-PDIM metabolism	0.79	0.05	0.86	0.15
ZINC15613697	280.3211	C17H16N2O2	CD36	1.16	0.07	1.06	0.13
ZINC15613699	280.3211	C17H16N2O2	CD36	1.04	0.07	0.98	0.02
ZINC1562944,	283.3251	C16H17N3O2	E3 ligase	1.00	0.16	1.07	0.25
ZINC15642747	255.697	C12H14ClNO3	TRP path	0.96	0.03	0.99	0.03
ZINC15774119	279.3348	C14H21N3O3	ATPase	0.96	0.01	1.05	0.09
ZINC15782434	213.2319	C13H11NO2	Lip	0.99	0.08	0.83	0.09
ZINC15879105	313.3941	C18H23N3O2	HIS path	0.98	0.11	1.16	0.14
ZINC16024434	262.692	C13H11ClN2O2	CD36	0.87	0.03	1.12	0.23
ZINC16112885	344.337	C18H17FN2O4	CD36	1.09	0.06	1.07	0.21
ZINC16448855	256.056	C8H6BrN3O2	ATPase	1.13	0.29	0.73	0.07
ZINC16473860	341.4225	C20H24FN3O	ABC transporters	0.76	0.09	0.95	0.12

ZINC16491727	306.3798	C23H16N	E3 ligase	1.00	0.07	1.06	0.17
ZINC16545364	294.328	C16H10N2O2S	ATPase	2.11	1.41	1.31	0.07
ZINC16577733	282.187	C10H13Cl2NO2S	Cations channels	0.86	0.04	1.20	0.32
ZINC16578353	268.16	C9H11Cl2NO2S	Cations channels	0.86	0.05	1.13	0.20
ZINC16578472	268.16	C9H11Cl2NO2S	Cations channels	0.86	0.08	1.06	0.11
ZINC16578857	296.213	C11H15Cl2NO2S	Cations channels	0.81	0.08	1.03	0.05
ZINC16640296	260.2636	C14H13FN2O2	CD36	1.11	0.08	0.92	0.04
ZINC16649304	333.831	C14H20ClNO4S	TRP path	1.06	0.16	0.97	0.08
ZINC16652469	311.44	C16H25NO3S	TRP path	1.19	0.19	0.94	0.05
ZINC16678216	252.376	C13H20N2OS	Proteasome I-lact-mg 132	0.96	0.08	0.93	0.23
ZINC16736406	253.467	C8H3Cl3O3	AKT1	1.09	0.06	0.98	0.15
ZINC16891583	216.041	C9H5Cl2O2	CYS path	0.78	0.06	0.97	0.08
ZINC16916510	268.334	C15H12N2OS	Cations channels	0.89	0.26	1.03	0.13
ZINC16917769	241.3282	C16H19NO	Proteasome inducers	0.96	0.15	0.97	0.06
ZINC16930431	255.2719	C14H13N3O2	Autophagy	1.58	0.11	0.78	0.13
ZINC16930459	275.69	C13H10ClN3O2	Autophagy	1.52	0.13	0.89	0.17
ZINC16943069	258.2693	C15H14O4	AKT1	1.07	0.26	0.55	0.47
ZINC16951570	272.3389	C17H20O3	Proteasome inducers	0.92	0.08	1.01	0.20
ZINC16951840	314.3358	C17H18N2O4	Proteasome inducers	0.98	0.17	1.11	0.26
ZINC16952013	332.477	C21H32O3	ATPase	1.05	0.19	0.86	0.17
ZINC16952431	304.2946	C16H16O6	AKT1	0.47	0.18	0.68	0.19
ZINC16968596	289.3941	C20H21N2	GLN path	1.50	0.44	0.97	0.05
ZINC16969950	273.37	C17H23NO2	Proteasome inducers	0.97	0.07	0.95	0.12
ZINC16990833	306.4397	C19H30O3	Proteasome inducers	1.00	0.10	1.01	0.16
ZINC17021129	192.1946	C9H10N3O2	CYS path	1.08	0.01	0.96	0.04
ZINC17027349	327.2933	C12H17N5O6	ATPase	0.95	0.17	0.98	0.09
ZINC17060316	307.56	C11H9Cl3N2O2	TRP path	1.00	0.04	1.06	0.06
ZINC17145720	298.3397	C16H18N4O2	MeC	0.97	0.11	1.01	0.08
ZINC17148901	334.3703	C16H22N4O4	Proteasome I-lact-mg 132	0.78	0.01	0.91	0.03
ZINC17159011	314.3988	C19H24NO3	Proteasome inducers	0.90	0.11	1.07	0.08
ZINC17198140	286.372	C14H10N2OS2	E3 ligase	0.93	0.06	0.94	0.04
ZINC17209504	273.2872	C14H15N3O3	E3 ligase	0.88	0.04	1.04	0.10
ZINC17224323	209.2432	C14H11NO	E3 ligase	0.98	0.11	0.94	0.23
ZINC17256022	242.2301	C13H10N2O3	TRP path	0.99	0.10	0.97	0.03
ZINC17256799	319.2678	C18H9NO5	HIS path	1.06	0.12	0.96	0.07
ZINC17307469	310.3471	C18H18N2O3	CD36	0.92	0.08	1.10	0.11
ZINC17315857	310.3471	C18H18N2O3	CD36	0.94	0.11	1.03	0.17
ZINC17315872	290.3127	C19H14O3	TRP path	0.86	0.01	0.82	0.24

ZINC17316716	236.2686	C15H12N2O	CD36	1.15	0.13	0.78	0.17
ZINC17324694	228.2896	C14H16N2O	Proteasome inducers	0.98	0.04	1.03	0.06
ZINC17352663	342.4089	C20H24NO4	Proteasome inducers	0.60	0.19	1.00	0.06
ZINC17824005	311.312	C17H15N2O4	ATPase	1.18	0.18	0.83	0.08
ZINC17919572	278.2854	C16H12N3O2	GLN path	1.14	0.13	0.97	0.18
ZINC18046806	331.389	C15H11N2O3S2	E3 ligase	1.01	0.04	0.99	0.05
ZINC18060909	284.29	C15H14N3O3	E3 ligase	0.89	0.06	1.05	0.10
ZINC18066566	282.3403	C16H18N4O	MeC	0.98	0.09	0.92	0.25
ZINC18126402	282.294	C16H14N2O3	ATPase	1.38	0.18	0.75	0.07
ZINC18126759	287.38	C15H17N3OS	E3 ligase	0.88	0.06	1.05	0.06
ZINC18148554	284.3131	C15H16N4O2	MeC	0.99	0.06	1.02	0.05
ZINC18148632	269.3018	C14H15N5O	MeC	1.08	0.05	1.11	0.15
ZINC18148698	310.3504	C17H18N4O2	MeC	0.95	0.24	1.00	0.15
ZINC18182025	263.316	C12H13N3O2S	E3 ligase	0.98	0.03	1.02	0.11
ZINC18188334	323.369	C17H13N3O2S	ATPase	1.89	0.23	0.86	0.14
ZINC18191055	310.3504	C17H18N4O2	Proteasome inducers	0.87	0.05	1.01	0.08
ZINC18199487	329.374	C16H15N3O3S	E3 ligase	0.89	0.03	1.03	0.04
ZINC18219412	299.3228	C16H16FN4O	E3 ligase	0.80	0.07	1.08	0.07
ZINC18324877	312.3663	C17H20N4O2	MeC	1.18	0.12	1.11	0.14
ZINC18847046	197.2557	C13H13N2	AKT1	1.04	0.15	0.96	0.19
ZINC19088942	193.63	C10H8ClNO	CD36	0.87	0.08	0.91	0.01
ZINC19094215	222.2173	C11H12NO4	TRP path	0.82	0.14	0.97	0.12
ZINC19118134	315.3421	C17H18FN3O2	ATPase	1.03	0.13	1.11	0.18
ZINC19275542	229.2744	C14H15NO2	Autophagy	1.40	0.07	1.10	0.30
ZINC19364225	148.2498	C6H20N4	Porins	0.94	0.05	0.93	0.09
ZINC19366504	161.2883	C8H23N3	Porins	1.03	0.04	1.12	0.12
ZINC19388912	268.2873	C16H14NO3	CYS path	0.96	0.04	0.95	0.07
ZINC19419604	259.3037	C14H17N3O2	Cations channels	0.99	0.04	0.62	0.26
ZINC19535208	324.4168	C20H24N2O2	ABC transporters	0.84	0.08	1.07	0.15
ZINC19594398	267.2826	C15H13N3O2	Proteasome inducers	1.02	0.03	1.02	0.17
ZINC19597031	330.444	C18H22N2O2S	ABC transporters	0.83	0.06	1.05	0.17
ZINC19597033	344.471	C19H24N2O2S	ABC transporters	1.02	0.03	0.94	0.16
ZINC19637456	243.4319	C14H33N3	Porins	0.80	0.12	0.84	0.11
ZINC19701792	247.3129	C14H19N2O2	CD36	0.87	0.10	1.07	0.12
ZINC19702027	265.3297	C17H17N2O	CD36	0.89	0.11	1.07	0.16
ZINC19726474	209.649	C11H10ClO2	CYS path	0.99	0.08	0.83	0.17
ZINC19792324	324.4168	C20H24N2O2	ABC transporters	1.07	0.05	0.96	0.11
ZINC19793781	268.312	C11H14N3O3S	TRP path	0.98	0.11	1.07	0.30

ZINC19793833	260.696	C9H8ClFN3OS	TRP path	1.06	0.11	0.73	0.28
ZINC19801713	253.362	C16H15NS	Proteasome inducers	1.05	0.12	0.34	0.31
ZINC19816655	288.3681	C15H22N5O	Proteasome inducers	0.90	0.06	0.83	0.06
ZINC19823301	294.3908	C19H22N2O	ABC transporters	0.96	0.06	0.96	0.07
ZINC19837230	207.2689	C12H17NO2	Autophagy	1.03	0.09	0.89	0.16
ZINC19862693	323.432	C20H25N3O	ABC transporters	1.07	0.13	0.95	0.19
ZINC20031356	259.668	C13H8ClN2O2	Proteasome I-lact-mg 132	0.93	0.13	1.13	0.07
ZINC20054269	201.3522	C11H27N3	Porins	0.81	0.09	1.17	0.12
ZINC20168500	342.4072	C20H23FN2O2	ABC transporters	1.13	0.12	1.02	0.16
ZINC20220558	310.3902	C19H22N2O2	ABC transporters	1.39	0.05	1.00	0.14
ZINC20265141	234.3803	C15H26N2	Porins	0.80	0.07	1.16	0.12
ZINC20446024	290.1755	C10H7F3N3O4	TRP path	0.02	0.01	0.05	0.19
ZINC20457666	321.711	C15H11ClFN2O3	CYS path	1.00	0.13	0.96	0.12
ZINC20639327	409.5643	C25H35N3O2	adamantane scaffold	0.98	0.16	1.05	0.13
ZINC20722689	300.402	C16H24N6	Calcineurin1	1.08	0.05	1.06	0.14
ZINC20727282	329.374	C16H15N3O3S	Proteasome I-lact-mg 132	1.19	0.28	0.97	0.11
ZINC20738939	338.3572	C19H18N2O4	GLN path	0.87	0.03	1.14	0.11
ZINC20855643	234.685	C11H11ClN4	Calcineurin1	0.95	0.13	1.03	0.13
ZINC20865587	345.459	C18H23N3O2S	GLN path	1.01	0.06	0.99	0.16
ZINC20865618	331.433	C17H21N3O2S	GLN path	0.98	0.04	0.95	0.16
ZINC20941386	339.4513	C21H27N2O2	Autophagy	1.12	0.07	0.95	0.06
ZINC21216205	319.334	C14H12FN4O2S	GLN path	0.85	0.09	0.92	0.01
ZINC21298412	205.064	C6H2Cl2N2S	AKT1	1.02	0.07	0.88	0.07
ZINC21595884	307.3034	C17H13N3O3	HIS path	0.97	0.12	0.88	0.08
ZINC21627879	328.386	C17H16N2O3S	FASII-PDIM metabolism	0.82	0.08	0.87	0.07
ZINC21695672	312.34	C18H17FN2O2	HIS path	0.99	0.07	0.94	0.01
ZINC21822271	324.4399	C20H26N3O	ABC transporters	0.76	0.12	1.06	0.10
ZINC21889348	312.3199	C17H16N2O4	CD36	1.02	0.01	1.04	0.09
ZINC22035501	133.2352	C6H19N3	Porins	0.98	0.03	0.81	0.08
ZINC22057034	199.037	C8H4Cl2N2	AKT1	1.12	0.04	0.89	0.08
ZINC22204687	260.312	C13H12N2O2S	Autophagy	0.79	0.02	1.01	0.03
ZINC22285501	310.413	C18H18N2OS	FASII-PDIM metabolism	0.80	0.12	0.85	0.06
ZINC22333955	324.4168	C20H24N2O2	ABC transporters	1.03	0.04	0.89	0.09
ZINC22339830	229.3175	C15H19NO	Proteasome inducers	0.89	0.11	0.97	0.10
ZINC22384970	349.4262	C21H23N3O2	ABC transporters	1.09	0.08	1.31	0.34
ZINC22385002	330.401	C17H18N2O3S	FASII-PDIM metabolism	1.22	0.06	0.89	0.21
ZINC22506998	311.3352	C17H17N3O3	CD36	1.17	0.10	0.95	0.09
ZINC22507003	325.3618	C18H19N3O3	CD36	0.97	0.07	0.89	0.06

ZINC22585748	338.4665	C21H28N3O	ABC transporters	0.90	0.04	1.11	0.21
ZINC22589796	282.294	C16H14N2O3	CD36	1.04	0.01	1.04	0.13
ZINC22597502	290.381	C15H18N2O2S	FASII-PDIM metabolism	0.95	0.09	0.97	0.03
ZINC22597948	313.371	C17H15NO3S	FASII-PDIM metabolism	0.76	0.02	0.87	0.10
ZINC22679873	272.365	C15H16N2OS	Lip	0.88	0.06	0.96	0.10
ZINC22680085	275.341	C15H14FNOS	Lip	1.08	0.08	1.00	0.05
ZINC22682599	266.359	C13H18N2O2S	Lip	1.03	0.02	1.01	0.03
ZINC22795870	324.4399	C20H26N3O	ABC transporters	1.01	0.03	1.02	0.08
ZINC22803485	298.4027	C18H24N3O	ABC transporters	1.00	0.15	1.12	0.14
ZINC22803490	298.4027	C18H24N3O	ABC transporters	0.86	0.15	0.82	0.16
ZINC22832298	307.3895	C19H21N3O	PI3K	1.06	0.11	0.97	0.12
ZINC22861291	338.4665	C21H28N3O	ABC transporters	0.79	0.09	1.14	0.09
ZINC22916039	338.4665	C21H28N3O	ABC transporters	1.00	0.07	0.87	0.08
ZINC22968205	266.3376	C17H18N2O	Lip	1.15	0.10	0.92	0.02
ZINC23135661	326.3648	C19H19FN2O2	Lip	1.04	0.06	0.92	0.04
ZINC23280910	277.425	C17H29N2O	TRP path	0.85	0.05	0.88	0.11
ZINC23709262	298.745	C12H11ClN2O3S	HIS path	1.24	0.19	0.77	0.16
ZINC23715248	308.3743	C19H20N2O2	Lip	1.17	0.12	0.84	0.16
ZINC23749793	349.4494	C21H25N4O	ABC transporters	1.00	0.02	0.97	0.13
ZINC24032070	295.3788	C18H21N3O	ABC transporters	1.05	0.08	1.05	0.11
ZINC24052419	334.456	C16H18N2O2S2	FASII-PDIM metabolism	0.98	0.10	0.97	0.13
ZINC24115542	338.4036	C19H22N4O2	GLN path	0.80	0.12	0.98	0.05
ZINC24325591	322.4009	C20H22N2O2	CD36	1.19	0.06	0.96	0.06
ZINC24341830	329.3984	C20H19N5	GLN path	0.99	0.19	0.95	0.03
ZINC24471158	334.436	C16H22N4O2S	PI3K	1.04	0.03	1.00	0.08
ZINC24576920	241.2851	C15H15NO2	Autophagy	0.87	0.10	0.92	0.01
ZINC24639222	346.3858	C19H18N6O	ATPase	0.91	0.05	0.92	0.09
ZINC24745520	280.3642	C18H20N2O	Lip	1.12	0.02	0.95	0.05
ZINC24830200	330.332	C16H10F2N3OS	GLN path	0.91	0.11	0.99	0.06
ZINC25612041	305.3291	C15H19N3O4	HIS path	0.87	0.09	0.92	0.06
ZINC25824395	262.3076	C13H18N4O2	Calcineurin1	1.04	0.08	0.93	0.04
ZINC25898566	338.3572	C19H18N2O4	CD36	1.00	0.01	0.89	0.07
ZINC25913279	329.42	C16H19N5OS	Calcineurin1	1.06	0.02	0.86	0.07
ZINC26161323	323.3889	C19H21N3O2	ABC transporters	0.79	0.06	1.06	0.08
ZINC26475886	273.2441	C13H11N3O4	E3 ligase	0.92	0.02	0.98	0.05
ZINC26513872	210.071	C9H8BrN	AKT1	0.86	0.08	0.73	0.08
ZINC26523025	288.388	C16H24N4O	Calcineurin1	1.00	0.02	0.93	0.09
ZINC26571805	325.3187	C17H15N3O4	ATPase	0.98	0.06	0.99	0.16

ZINC26903089	255.337	$C_{11}H_{17}N_3O_2S$	Autophagy	0.88	0.12	0.98	0.05
ZINC27288222	345.3499	$C_{17}H_{19}N_3O_5$	GLN path	0.95	0.04	0.94	0.05
ZINC27663145	319.442	$C_{16}H_{17}NO_2S_2$	FASII-PDIM metabolism	1.06	0.10	0.82	0.06
ZINC27788872	305.2875	$C_{17}H_{11}N_3O_3$	E3 ligase	0.88	0.01	0.99	0.07
ZINC27904853	348.458	$C_{18}H_{23}FN_3OS$	ABC transporters	0.93	0.11	1.02	0.12
ZINC28162270	233.333	$C_{12}H_{15}N_3S$	Autophagy	1.01	0.00	1.14	0.13
ZINC28162708	271.338	$C_{14}H_{13}N_3OS$	Autophagy	1.08	0.14	1.09	0.20
ZINC28220828	308.3345	$C_{17}H_{16}N_4O_2$	GLN path	0.89	0.02	0.99	0.17
ZINC28238510	347.775	$C_{16}H_{15}ClFN_5O$	GLN path	0.93	0.07	0.92	0.04
ZINC28245834	323.3922	$C_{18}H_{21}N_5O$	GLN path	1.28	0.08	1.02	0.11
ZINC28463490	227.323	$C_{11}H_{17}NO_2S$	Cations channels	1.07	0.03	0.99	0.09
ZINC28517371	319.4002	$C_{20}H_{21}N_3O$	FASII-PDIM metabolism	0.87	0.08	0.93	0.01
ZINC28645985	274.3614	$C_{15}H_{22}N_4O$	Calcineurin1	0.98	0.09	0.93	0.08
ZINC28962120	288.388	$C_{14}H_{12}N_2OS_2$	TRP path	1.05	0.03	1.13	0.03
ZINC28968007	323.4088	$C_{20}H_{23}N_2O_2$	Autophagy	1.02	0.11	1.01	0.04
ZINC29078308	332.3559	$C_{19}H_{16}N_4O_2$	HIS path	0.90	0.06	0.91	0.05
ZINC29597329	330.444	$C_{18}H_{22}N_2O_2S$	Autophagy	0.96	0.04	0.95	0.08
ZINC29634790	324.4399	$C_{20}H_{26}N_3O$	ABC transporters	0.83	0.10	1.05	0.04
ZINC29748127	326.3896	$C_{19}H_{22}N_2O_3$	GLN path	0.89	0.23	1.11	0.02
ZINC29912862	274.3349	$C_{16}H_{20}NO_3$	Proteasome inducers	1.06	0.10	0.91	0.02
ZINC30335231	303.35	$C_{13}H_{18}FNO_4S$	PI3K	1.03	0.04	0.99	0.10
ZINC30678411	249.515	$C_6H_2BrClN_2S$	Autophagy	1.02	0.01	1.00	0.04
ZINC31255544	323.131	$C_{14}H_8Cl_2N_2O_3$	AKT1	0.96	0.01	1.01	0.12
ZINC313218	238.2812	$C_{16}H_{14}O_2$	E3 ligase	0.89	0.09	0.71	0.08
ZINC31627888	281.331	$C_{12}H_{15}N_3O_3S$	Autophagy	1.04	0.03	0.92	0.13
ZINC31716598	316.4411	$C_{18}H_{28}N_4O$	Calcineurin1	0.75	0.03	1.01	0.18
ZINC31775965	280.298	$C_{17}H_{14}NO_3$	CYS path	0.85	0.02	0.72	0.11
ZINC32064144	324.4168	$C_{20}H_{24}N_2O_2$	ATPase	0.85	0.20	0.86	0.13
ZINC32096020	242.3162	$C_{15}H_{18}N_2O$	FASII-PDIM metabolism	0.87	0.10	1.02	0.17
ZINC32103660	324.3306	$C_{18}H_{16}N_2O_4$	GLN path	0.85	0.08	1.06	0.06
ZINC32107862	254.3204	$C_{14}H_{21}FNO_2$	Autophagy	0.95	0.07	0.91	0.05
ZINC32558440	272.347	$C_{10}H_{18}N_5O_2S$	PI3K	1.09	0.11	1.02	0.06
ZINC32598899	252.68	$C_9H_5ClN_4OS$	E3 ligase	0.85	0.06	0.99	0.11
ZINC32610058	346.3065	$C_{17}H_{13}F_3N_4O$	GLN path	0.91	0.03	0.92	0.12
ZINC32619703	349.449	$C_{20}H_{19}N_3OS$	PI3K	1.25	0.15	0.84	0.08
ZINC32627551	192.645	$C_{10}H_9ClN_2$	Proteasome I-lact-mg 132	0.93	0.11	0.88	0.10
ZINC32648393	262.3242	$C_{15}H_{20}NO_3$	CYS path	0.86	0.06	0.96	0.08
ZINC32679759	339.4513	$C_{21}H_{27}N_2O_2$	ABC transporters	1.04	0.01	0.94	0.11

ZINC32754667	317.3844	C20H19N3O	PI3K	1.08	0.10	0.89	0.06
ZINC32836638	300.375	C16H16N2O2S	CD36	1.13	0.10	0.94	0.05
ZINC32836662	308.3743	C19H20N2O2	CD36	1.11	0.04	1.01	0.12
ZINC32836687	284.3098	C16H16N2O3	CD36	1.03	0.05	0.87	0.08
ZINC32836750	314.402	C17H18N2O2S	CD36	1.03	0.01	1.06	0.07
ZINC32836861	300.375	C16H16N2O2S	CD36	1.02	0.05	1.04	0.12
ZINC32863530	297.416	C14H23N3O2S	HIS path	0.90	0.12	0.92	0.06
ZINC32891503	283.3681	C17H21N3O	PI3K	1.05	0.09	0.94	0.06
ZINC32905311	320.385	C20H20N2O2	CD36	0.83	0.06	1.06	0.29
ZINC32908302	334.4116	C21H22N2O2	CD36	1.04	0.03	0.94	0.06
ZINC32909802	269.115	C11H11BrNO2	Autophagy	0.83	0.11	0.88	0.06
ZINC32911348	269.448	C5H3ClIN2O	Calcineurin1	1.04	0.12	0.91	0.31
ZINC32911391	251.0483	C5H8IN4	AKT1	0.87	0.15	1.03	0.14
ZINC32911392	269.471	C5H5ClIN3	AKT1	0.96	0.12	0.67	0.19
ZINC32914405	340.4	C17H16N4O2S	FASII-PDIM metabolism	0.96	0.02	0.90	0.13
ZINC32974093	316.3948	C18H24N2O3	GLN path	0.92	0.07	1.07	0.11
ZINC33127202	329.3008	C17H13F2N3O2	CD36	0.99	0.08	1.15	0.14
ZINC33127215	316.714	C16H10ClFN2O2	CD36	0.88	0.08	0.82	0.36
ZINC33184726	329.3935	C18H23N3O3	ATPase	0.92	0.08	1.04	0.15
ZINC33662987	302.3649	C18H22O4	Proteasome I-lact-mg 132	0.92	0.09	0.90	0.17
ZINC33821657	329.4565	C20H29N2O2	Proteasome inducers	0.80	0.08	0.92	0.07
ZINC33891765	258.1734	C11H7F3NO3	TRP path	1.04	0.03	1.06	0.07
ZINC33971762	276.3541	C15H22N3O2	Proteasome inducers	0.99	0.15	0.91	0.13
ZINC34493133	211.691	C10H14ClN3	Calcineurin1	0.89	0.04	0.89	0.13
ZINC35053122	319.359	C14H15N4O3S	E3 ligase	0.90	0.01	1.00	0.05
ZINC35622355	251.2784	C13H17NO4	TRP path	0.87	0.06	0.98	0.09
ZINC35853669	338.4665	C21H28N3O	ABC transporters	0.80	0.08	1.07	0.03
ZINC36095658	344.385	C18H22N3O4	Proteasome I-lact-mg 132	0.92	0.06	1.01	0.12
ZINC36101987	279.3315	C15H21NO4	PI3K	0.80	0.18	1.17	0.05
ZINC36235782	336.3877	C19H20N4O2	GLN path	0.93	0.01	0.85	0.06
ZINC36359834	281.374	C13H19N3O2S	HIS path	0.81	0.05	0.96	0.12
ZINC36367263	337.3956	C19H21N4O2	ATPase	0.98	0.02	0.93	0.05
ZINC36390249	338.4036	C19H22N4O2	ABC transporters	1.08	0.09	1.11	0.19
ZINC36390765	334.4116	C21H22N2O2	CD36	1.10	0.06	0.96	0.05
ZINC36395303	272.2991	C15H16N2O3	HIS path	0.94	0.15	0.91	0.07
ZINC36610855	283.3251	C16H17N3O2	Proteasome inducers	0.96	0.09	1.07	0.14
ZINC36709040	340.4	C17H16N4O2S	Proteasome I-lact-mg 132	0.95	0.12	0.92	0.07
ZINC36729401	344.38	C19H21FN2O3	GLN path	1.04	0.01	1.06	0.06

ZINC37181216	329.824	C18H20ClN3O	ABC transporters	1.04	0.01	0.95	0.14
ZINC37207185	261.3196	C14H19N3O2	ABC transporters	1.00	0.03	0.89	0.10
ZINC37380915	230.067	C10H7Cl2O2	CYS path	1.26	0.30	0.78	0.15
ZINC37868918	285.2731	C15H12FN3O2	E3 ligase	0.92	0.10	1.04	0.04
ZINC38140758	302.3881	C18H24NO3	CYS path	1.04	0.05	0.87	0.08
ZINC38339237	238.071	C6H3Cl2N2O2S	Calcineurin1	0.88	0.02	1.00	0.15
ZINC38489656	266.3113	C14H19FN2O2	Lip	0.97	0.07	0.97	0.10
ZINC38489658	290.356	C15H15FN2OS	Lip	0.98	0.15	1.03	0.27
ZINC38489659	304.382	C16H17FN2OS	Lip	1.09	0.26	0.85	0.09
ZINC38489669	328.3806	C19H21FN2O2	Lip	1.01	0.18	0.90	0.10
ZINC38591529	298.3547	C18H19FN2O	Lip	0.96	0.13	0.86	0.14
ZINC38593140	286.392	C16H18N2OS	Lip	1.01	0.22	0.90	0.08
ZINC38593154	302.391	C16H18N2O2S	Lip	0.78	0.19	0.91	0.06
ZINC39162211	317.3596	C17H21N2O4	CYS path	0.95	0.04	1.08	0.10
ZINC39419150	349.4262	C21H23N3O2	CD36	1.03	0.04	1.01	0.13
ZINC40034706	311.401	C17H17N3OS	FASII-PDIM metabolism	1.07	0.09	1.02	0.10
ZINC40068659	327.397	C18H17NO3S	CD36	1.08	0.05	0.92	0.02
ZINC40119706	329.3406	C19H17F2NO2	GLN path	0.71	0.30	1.04	0.10
ZINC40122418	329.3406	C19H17F2NO2	GLN path	0.93	0.09	0.95	0.04
ZINC40153822	339.817	C16H22ClN3O3	HIS path	0.75	0.18	1.06	0.16
ZINC40159308	271.2103	C13H9F4NO	GLN path	0.83	0.17	1.08	0.05
ZINC40266810	313.3941	C18H23N3O2	FASII-PDIM metabolism	0.84	0.12	1.09	0.20
ZINC40288424	299.3444	C17H19N2O3	FASII-PDIM metabolism	0.86	0.09	0.93	0.19
ZINC40313364	261.3196	C14H19N3O2	Autophagy	0.95	0.18	0.98	0.16
ZINC40466211	306.4231	C17H28N3O2	Autophagy	0.88	0.06	0.92	0.06
ZINC40473631	291.392	C14H19N4OS	ABC transporters	1.22	0.03	0.91	0.06
ZINC40475356	324.3737	C19H20N2O3	CD36	0.96	0.02	1.00	0.06
ZINC40476545	299.2997	C16H14FN3O2	GLN path	0.71	0.33	1.09	0.04
ZINC40478932	309.384	C14H19N3O3S	Calcineurin1	0.90	0.09	0.98	0.11
ZINC40534033	331.433	C17H21N3O2S	GLN path	0.91	0.12	0.94	0.05
ZINC40540524	300.3011	C14H18F2N2O3	GLN path	0.94	0.18	1.12	0.13
ZINC40553290	342.389	C19H22N2O4	CD36	0.82	0.04	1.31	0.16
ZINC40553527	331.436	C16H21N5OS	HIS path	0.95	0.10	0.90	0.03
ZINC40555099	341.3546	C19H17F2N3O	PI3K	1.24	0.08	0.93	0.07
ZINC40564582	288.901	C5H3Cl2IN2	AKT1	0.94	0.05	1.09	0.24
ZINC40758713	285.384	C17H23N3O	FASII-PDIM metabolism	1.09	0.04	1.09	0.09
ZINC41074114	348.3322	C19H14N3O4	E3 ligase	0.91	0.05	0.82	0.10
ZINC41122925	338.4036	C19H22N4O2	GLN path	0.99	0.04	0.91	0.14

ZINC41122963	339.3916	$C_{18}H_{21}N_5O_2$	GLN path	0.85	0.10	1.00	0.19
ZINC41220323	338.4003	$C_{20}H_{22}N_2O_3$	Lip	1.04	0.09	1.09	0.19
ZINC41417063	332.3182	$C_{15}H_{19}F_3N_2O_3$	Proteasome I-lact-mg 132	0.88	0.07	0.93	0.06
ZINC41470719	264.121	$C_{11}H_{10}BrN_3$	Autophagy	1.10	0.10	0.96	0.15
ZINC41470837	252.111	$C_{10}H_{10}BrN_3$	Autophagy	1.02	0.07	0.40	0.05
ZINC41471029	271.2136	$C_{12}H_9F_4N_3$	Autophagy	0.92	0.15	0.81	0.16
ZINC41471124	219.2581	$C_{12}H_{14}FN_3$	Autophagy	1.04	0.14	0.85	0.19
ZINC41474560	257.2663	$C_{13}H_{12}FN_5$	PI3K	0.92	0.08	1.18	0.23
ZINC41474727	251.122	$C_{11}H_{11}BrN_2$	Autophagy	0.82	0.23	1.14	0.16
ZINC41474783	206.671	$C_{11}H_{11}ClN_2$	Autophagy	1.13	0.17	0.99	0.08
ZINC41475133	303.3562	$C_{16}H_{21}N_3O_3$	PI3K	0.99	0.17	1.08	0.29
ZINC41510815	281.735	$C_{14}H_{16}ClNO_3$	Proteasome inducers	0.85	0.09	1.05	0.04
ZINC41534355	237.725	$C_{13}H_{16}ClNO$	Lip	0.82	0.07	0.98	0.07
ZINC41534386	221.2706	$C_{13}H_{16}FNO$	Lip	0.97	0.11	0.90	0.11
ZINC41535169	235.2972	$C_{14}H_{18}FNO$	Lip	1.04	0.12	0.88	0.13
ZINC41564693	245.249	$C_{14}H_{12}FNO_2$	Lip	0.93	0.10	0.96	0.09
ZINC41566291	238.713	$C_{12}H_{15}ClN_2O$	Lip	0.95	0.10	0.95	0.06
ZINC41566825	255.2869	$C_{16}H_{14}FNO$	Lip	0.86	0.11	1.36	0.23
ZINC41567494	286.756	$C_{16}H_{15}ClN_2O$	Lip	1.24	0.11	0.93	0.28
ZINC41569497	249.3056	$C_{14}H_{19}NO_3$	Lip	0.99	0.08	0.85	0.23
ZINC41569525	281.3474	$C_{15}H_{23}NO_4$	PI3K	0.98	0.12	1.13	0.27
ZINC41569589	239.698	$C_{12}H_{14}ClNO_2$	Lip	1.03	0.14	1.13	0.27
ZINC41569851	250.3119	$C_{14}H_{19}FN_2O$	Lip	0.97	0.15	1.12	0.28
ZINC42177448	315.39	$C_{16}H_{17}N_3O_2S$	GLN path	0.90	0.07	1.03	0.02
ZINC42196603	343.4863	$C_{20}H_{31}N_4O$	ATPase	1.07	0.08	0.95	0.11
ZINC42196617	339.4961	$C_{18}H_{35}N_4O_2$	ATPase	1.41	0.45	1.14	0.12
ZINC42231108	286.3257	$C_{16}H_{18}N_2O_3$	HIS path	0.91	0.09	0.91	0.03
ZINC42311721	348.3553	$C_{19}H_{16}N_4O_3$	ATPase	0.79	0.21	0.92	0.06
ZINC42985608	247.742	$C_{10}H_{14}ClNO_2S$	Cations channels	1.33	0.08	0.84	0.11
ZINC43266510	266.2864	$C_{14}H_{16}F_2N_2O$	TRP path	0.97	0.07	0.99	0.05
ZINC43302496	263.3553	$C_{15}H_{23}N_2O_2$	Autophagy	0.70	0.11	1.01	0.12
ZINC43532188	272.3289	$C_{13}H_{18}N_7$	PI3K	0.98	0.03	1.08	0.07
ZINC43710509	313.378	$C_{15}H_{15}N_5OS$	FASII-PDIM metabolism	0.97	0.05	1.03	0.15
ZINC43827469	263.676	$C_{13}H_{10}ClNO_3$	ATPase	0.65	0.13	0.68	0.25
ZINC44695731	338.3572	$C_{19}H_{18}N_2O_4$	CD36	0.76	0.09	1.04	0.19
ZINC44717703	313.3229	$C_{18}H_{16}FNO_3$	GLN path	1.08	0.04	0.94	0.04
ZINC44836360	271.3541	$C_{17}H_{21}NO_2$	TRP path	0.73	0.10	0.94	0.10
ZINC44839708	324.3737	$C_{19}H_{20}N_2O_3$	CD36	0.84	0.14	1.01	0.13

ZINC44845789	275.3263	C14H19N4O2	Calcineurin1	0.99	0.07	1.16	0.13
ZINC44845813	289.3528	C15H21N4O2	Calcineurin1	1.02	0.07	1.08	0.08
ZINC44889411	340.3731	C19H20N2O4	CD36	1.01	0.05	1.05	0.12
ZINC44894494	273.353	C14H15N3OS	FASII-PDIM metabolism	0.81	0.04	0.96	0.14
ZINC44909316	298.3364	C17H18N2O3	CD36	0.78	0.08	1.05	0.08
ZINC44912580	316.3981	C17H24N4O2	Calcineurin1	0.83	0.14	1.05	0.12
ZINC44912636	297.734	C14H16ClNO4	PI3K	0.92	0.10	0.92	0.05
ZINC44920831	299.3675	C17H21N3O2	FASII-PDIM metabolism	0.83	0.10	0.97	0.18
ZINC44936948	333.409	C15H19N5O2S	HIS path	0.79	0.31	1.08	0.04
ZINC44954921	333.381	C16H16FN3O2S	Lip	0.88	0.01	0.88	0.05
ZINC44967378	340.825	C15H17ClN2O3S	Proteasome I-lact-mg 132	0.70	0.28	0.86	0.19
ZINC45051482	332.57	C12H8Cl3N3O2	CD36	0.94	0.20	1.17	0.17
ZINC45248701	266.3376	C17H18N2O	Lip	1.06	0.14	1.03	0.11
ZINC45405652	296.792	C15H21ClN2O2	Autophagy	0.86	0.11	1.23	0.22
ZINC45496588	290.3144	C15H18N2O4	HIS path	1.02	0.13	0.98	0.03
ZINC45737099	349.3999	C18H24FN3O3	GLN path	0.94	0.06	0.91	0.13
ZINC46025851	338.427	C18H18N4OS	PI3K	1.08	0.09	0.93	0.10
ZINC46087064	332.3975	C17H24N4O3	GLN path	0.87	0.04	0.96	0.20
ZINC46087981	324.377	C18H20N4O2	GLN path	0.90	0.08	1.00	0.13
ZINC46156834	316.418	C17H20N2O2S	FASII-PDIM metabolism	1.09	0.09	0.82	0.10
ZINC46189491	344.385	C17H16N2O4S	CD36	0.81	0.13	1.25	0.23
ZINC46223699	299.2964	C17H14FNO3	GLN path	0.99	0.04	0.85	0.02
ZINC47510436	344.3337	C18H15F3N4	PI3K	1.73	0.49	0.70	0.35
ZINC47555458	318.394	C15H18N4O2S	GLN path	0.97	0.04	0.94	0.02
ZINC47615501	331.3895	C17H23N4O3	ATPase	1.04	0.06	1.03	0.31
ZINC47629469	339.3883	C19H21N3O3	CD36	0.76	0.11	1.01	0.12
ZINC47717893	250.3119	C14H19FN2O	Lip	1.01	0.06	0.96	0.11
ZINC47816097	267.099	C11H9BrNO2	Autophagy	1.01	0.10	0.96	0.10
ZINC47822249	255.767	C11H14ClN3S	HIS path	0.97	0.03	0.93	0.11
ZINC47843755	251.3064	C15H15N4	ABC transporters	0.86	0.07	0.80	0.22
ZINC47871828	262.3474	C15H22N2O2	HIS path	0.98	0.03	1.03	0.05
ZINC48262069	344.471	C19H24N2O2S	GLN path	0.83	0.20	0.96	0.05
ZINC48301260	306.3602	C15H22N4O3	HIS path	0.89	0.09	1.04	0.06
ZINC48301736	317.4259	C18H27N3O2	GLN path	0.92	0.17	0.93	0.05
ZINC48302374	332.421	C16H20N4O2S	GLN path	0.92	0.12	0.97	0.05
ZINC48398021	281.761	C12H12ClN3OS	HIS path	1.07	0.04	0.88	0.02
ZINC48439120	267.3257	C16H17N3O	PI3K	1.02	0.07	0.87	0.10
ZINC48543337	294.3644	C16H23FN2O2	Lip	1.09	0.12	0.94	0.12

ZINC48606732	261.339	C14H15NO2S	Autophagy	0.75	0.07	1.02	0.11
ZINC48627925	289.373	C15H17N2O2S	PI3K	1.06	0.11	0.88	0.06
ZINC48645485	256.303	C14H16N4O	PI3K	1.09	0.10	0.95	0.11
ZINC48732410	311.217	C14H19BrN2O	Lip	1.11	0.16	1.15	0.15
ZINC48735952	289.757	C16H16ClNO2	Autophagy	0.78	0.21	0.93	0.13
ZINC48921568	261.3179	C14H18FN4	Autophagy	1.16	0.19	1.09	0.07
ZINC49102197	319.207	C12H12Cl2N2O2S	Cations channels	0.74	0.10	0.90	0.09
ZINC49164724	330.335	C16H12NO5S	HIS path	1.09	0.03	1.08	0.04
ZINC49166352	297.2424	C15H9N2O5	HIS path	1.05	0.02	0.95	0.08
ZINC49337795	302.35	C11H18N4O4S	PI3K	1.01	0.04	1.12	0.18
ZINC49517723	269.138	C11H13BrN2O	Lip	1.07	0.02	1.03	0.13
ZINC49521031	288.365	C15H16N2O2S	Lip	1.20	0.19	0.97	0.15
ZINC49967885	277.3819	C16H25N2O2	Autophagy	0.69	0.11	0.96	0.06
ZINC50002921	295.3755	C19H21NO2	Autophagy	0.76	0.08	0.90	0.19
ZINC50682026	337.414	C17H27N3O4	Proteasome I-lact-mg 132	0.87	0.12	0.96	0.05
ZINC51654985	329.37	C17H15NO4S	CD36	0.84	0.12	0.90	0.07
ZINC52041717	339.4314	C20H25N3O2	GLN path	1.03	0.01	0.87	0.15
ZINC53018979	345.3681	C18H20FN3O3	GLN path	1.01	0.01	0.97	0.04
ZINC53095976	275.4091	C17H27N2O	Autophagy	0.82	0.17	1.08	0.04
ZINC53140302	317.2901	C16H13F2N3O2	GLN path	1.14	0.12	0.96	0.04
ZINC53324211	302.4112	C18H26N2O2	HIS path	1.00	0.01	1.15	0.10
ZINC53548588	305.399	C14H19N5OS	Proteasome I-lact-mg 132	0.82	0.10	1.09	0.11
ZINC53602235	319.4433	C21H25N3	ABC transporters	1.03	0.10	1.00	0.09
ZINC53811127	325.428	C19H25N4O	ABC transporters	0.76	0.10	0.99	0.06
ZINC53897486	316.3188	C15H19F3N2O2	Autophagy	0.93	0.04	1.13	0.04
ZINC53956503	324.4168	C20H24N2O2	ABC transporters	0.99	0.08	0.93	0.05
ZINC54281351	274.341	C13H14N4OS	Autophagy	0.72	0.22	0.95	0.05
ZINC54426346	296.3636	C18H20N2O2	Lip	0.83	0.08	0.90	0.15
ZINC54460091	316.4179	C18H26N3O2	ATPase	0.98	0.09	0.94	0.09
ZINC54701996	306.3584	C19H18N2O2	Lip	0.81	0.11	1.08	0.06
ZINC54916934	311.2673	C16H10FN3O3	HIS path	1.06	0.04	0.99	0.04
ZINC55068114	333.445	C18H23NO3S	PI3K	0.76	0.12	0.93	0.05
ZINC55119167	304.407	C16H20N2O2S	Proteasome I-lact-mg 132	1.14	0.27	0.89	0.10
ZINC55180444	262.331	C12H14N4OS	Proteasome inducers	0.85	0.54	0.92	0.19
ZINC55180527	276.357	C13H16N4OS	Proteasome inducers	0.78	0.08	1.04	0.07
ZINC55199689	328.857	C15H21ClN2O2S	TRP path	1.09	0.04	0.94	0.07
ZINC55207648	314.379	C17H16NO3S	CYS path	0.83	0.15	0.99	0.09
ZINC55393491	343.4249	C21H21N5	ATPase	1.00	0.05	0.88	0.03

ZINC55553157	337.803	C19H16ClN3O	PI3K	1.27	0.17	1.14	0.39
ZINC55553332	335.423	C19H17N3OS	PI3K	1.25	0.18	0.85	0.09
ZINC55564249	309.4054	C19H23N3O	ABC transporters	0.97	0.06	1.15	0.13
ZINC55742777	343.443	C18H21N3O2S	GLN path	0.86	0.08	1.08	0.07
ZINC55752973	337.4155	C20H23N3O2	GLN path	0.92	0.10	0.84	0.06
ZINC5586339	325.4081	C18H23N5O	E3 ligase	1.02	0.08	0.89	0.12
ZINC55866946	335.3996	C20H21N3O2	GLN path	0.85	0.11	1.04	0.07
ZINC56227017	330.3286	C18H16F2N2O2	GLN path	0.79	0.17	0.98	0.03
ZINC56499765	324.377	C18H20N4O2	GLN path	1.02	0.02	1.04	0.10
ZINC56679264	339.4314	C20H25N3O2	GLN path	0.97	0.27	0.88	0.13
ZINC57024255	323.432	C20H25N3O	ABC transporters	1.00	0.04	1.00	0.03
ZINC57024340	327.3959	C19H22FN3O	ABC transporters	1.15	0.02	0.86	0.03
ZINC57217532	193.046	C8H7Cl2F	Calcineurin1	1.02	0.09	1.11	0.07
ZINC57219381	320.3075	C14H19F3N2O3	Proteasome I-lact-mg 132	0.99	0.08	0.95	0.04
ZINC57220388	280.3627	C15H24N2O3	Proteasome I-lact-mg 132	0.78	0.08	0.96	0.06
ZINC57290201	251.2999	C13H18FN3O	ABC transporters	0.98	0.04	0.83	0.08
ZINC57315319	328.362	C17H14NO4S	CYS path	1.07	0.08	0.92	0.11
ZINC57322009	263.3354	C14H21N3O2	ABC transporters	0.77	0.05	0.98	0.21
ZINC57470883	264.3271			0.96	0.05	0.93	0.06
ZINC57508696	289.419	C15H21N4S	HIS path	0.91	0.11	1.09	0.03
ZINC57624716	286.392	C16H18N2OS	FASII-PDIM metabolism	1.12	0.04	0.88	0.12
ZINC57755227	246.3679	C16H24NO	Autophagy	0.70	0.08	0.99	0.16
ZINC57855340	318.434	C17H22N2O2S	Proteasome I-lact-mg 132	0.85	0.10	0.95	0.02
ZINC57899493	263.355	C14H17NO2S	GLN path	0.97	0.04	0.92	0.06
ZINC58016428	349.4262	C21H23N3O2	GLN path	0.92	0.07	0.93	0.06
ZINC58058562	310.3902	C19H22N2O2	Lip	0.93	0.07	0.90	0.18
ZINC58105060	318.3691	C20H18N2O2	GLN path	0.96	0.08	1.08	0.04
ZINC58203051	269.2607	C11H18F3NO3	TRP path	0.78	0.03	1.11	0.05
ZINC58244306	349.3832	C20H19N3O3	ATPase	1.23	0.13	0.87	0.08
ZINC58245568	319.442	C16H17NO2S2	FASII-PDIM metabolism	1.00	0.09	0.86	0.08
ZINC58278026	315.3125	C15H19F2NO4	PI3K	0.81	0.10	0.95	0.14
ZINC58323244	280.361	C15H23FN3O	ABC transporters	1.00	0.10	0.96	0.08
ZINC58325081	318.4371	C17H28N5O	PI3K	0.99	0.13	0.97	0.12
ZINC58356348	345.3433	C18H17F2N3O2	Proteasome I-lact-mg 132	0.91	0.14	1.01	0.06
ZINC58424110	330.4644	C20H30N2O2	GLN path	0.83	0.14	0.98	0.05
ZINC58527351	271.377	C16H17NOS	FASII-PDIM metabolism	1.05	0.09	0.82	0.06
ZINC58593284	253.146	C9H10Cl2O2S	Cations channels	0.98	0.08	0.82	0.09
ZINC58733284	329.3935	C18H23N3O3	HIS path	1.33	0.03	0.90	0.09

ZINC58759056	289.3694	C17H23NO3	PI3K	0.70	0.24
ZINC58759412	275.3428	C16H21NO3	PI3K	1.03	0.12
ZINC6383023	257.311	C14H17N4O	E3 ligase	0.95	0.05

1.01	0.15
0.84	0.06
1.07	0.09
