# Supplementary material for: Identification of Anti-Mycobacterium and Anti-Legionella Compounds With Potential Distinctive Structural Scaffolds From an HD-PBL Using Phenotypic Screens in Amoebae Host Models
Source: Front Microbiol. 2020 Feb 21;11:266. doi: 10.3389/fmicb.2020.00266 (PMC7047896; doi:10.3389/fmicb.2020.00266)
Supplement: TABLE S2 — Hit compounds characteristics: anti-infective, antibacterial, cytotoxic, and growth inhibitory data. [file Data_Sheet_2.PDF]

		Infection assay						Growth assay		Cytotoxicity		Antibiotic Assay				anti-virulence assay	Antibiotic Assay
		A. castellanii / *M. marinum* model		*A. castellanii* / *L. pneumophila*		*D. discoideum* / *M. marinum* model		*D. discoideum*		*A. castellanii*		*M. marinum*		*L. pneumophila*		*D. discoideum* / *M. marinum* model	*M. marinum*
Zinc Number	Hit from screens	30µM DMSO=1	stdev	30µM DMSO=1	stdev	30µM DMSO=1	stdev	30µM DMSO=1	stdev	30µM DMSO=1	stdev	30µM DMSO=1	stdev	30µM DMSO=1	stdev	phagocytic plaque assay 10µM	
ZINC00192269	*D. discoideum - M. marinum*					0.88	0.09	1.25	0.10	0.85	0.24	0.85	0.04			y	y
ZINC00254142	*D. discoideum - M. marinum*					0.81	0.08	0.94	0.11	0.95	0.21	0.95	0.06			y	y
ZINC00315276	*D. discoideum - M. marinum*					0.70	0.07	0.86	0.06	0.84	0.21	0.73	0.03			y	n
ZINC00899897	*D. discoideum - M. marinum*					1.00	0.13	0.97	0.06	0.84	0.21	0.96	0.02			y	y
ZINC00983974	*D. discoideum - M. marinum*					0.62	0.04	0.94	0.09	0.82	0.17	0.67	0.04			y	n
ZINC01580838	*D. discoideum - M. marinum*					0.83	0.14	0.92	0.05	0.89	0.12	1.04	0.04			y	y
ZINC03307107	*D. discoideum - M. marinum*					0.82	0.13	0.37	0.27	0.88	0.28	0.89	0.04			y	y
ZINC04688380	*D. discoideum - M. marinum*					0.59	0.16	0.99	0.03	0.85	0.18	0.94	0.10			y	n
ZINC04795898	*D. discoideum - M. marinum*					0.64	0.05	1.05	0.15	0.88	0.43	0.93	0.10			y	y
ZINC09007974	*D. discoideum - M. marinum*					0.80	0.06	0.94	0.11	0.81	0.17	0.88	0.05			y	y
ZINC16736406	*D. discoideum - M. marinum*					0.96	0.13	0.90	0.16	0.83	0.25	0.81	0.02			y	y
ZINC23709262	*D. discoideum - M. marinum*					0.55	0.15	1.08	0.10	0.90	0.18	1.07	0.06			y	y
ZINC26513872	*D. discoideum - M. marinum*					1.02	0.18	0.72	0.13	0.85	0.20	0.55	0.09			y	y
ZINC27904853	*D. discoideum - M. marinum*					0.77	0.05	1.03	0.09	0.95	0.26	0.88	0.04			y	n
ZINC04366006	*A. castellanii - L. pneumophila*			0.13	0.18	0.16	0.09	1.02	0.07	1.57	0.46			-0.04	0.07		
ZINC00058795	*A. castellanii - L. pneumophila*			0.01	0.01	1.11	0.26	0.88	0.07	0.66	0.20			-0.04	0.14		
ZINC00061012	*A. castellanii - L. pneumophila*			0.04	0.04	0.85	0.09	1.03	0.06	1.08	0.14			0.06	0.03		
ZINC04536395	*A. castellanii - L. pneumophila*			0.21	0.17	0.46	0.16	0.85	0.08	0.53	0.27			0.05	0.20		
ZINC00127447	*A. castellanii - L. pneumophila*			0.51	0.09	1.25	0.10	1.31	0.15	1.24	0.26			0.08	0.07		
ZINC01386521	*A. castellanii - L. pneumophila*			0.17	0.12	1.10	0.11	0.99	0.05	1.06	0.13			0.10	0.06		
ZINC00818584	*A. castellanii - L. pneumophila*			-0.02	0.02	0.75	0.12	0.88	0.08	0.96	0.12			0.44	0.15		
ZINC11990412	*A. castellanii - L. pneumophila*			0.36	0.13	1.06	0.16	1.02	0.04	1.08	0.18			0.31	0.16		
ZINC09306182	*A. castellanii - L. pneumophila*			0.41	0.04	1.17	0.15	1.13	0.05	0.96	0.33			0.27	0.13		
ZINC16943069	*A. castellanii - L. pneumophila*			0.55	0.21	0.54	0.07	0.81	0.03	1.25	0.38			0.35	0.22		
ZINC41470837	*A. castellanii - L. pneumophila*			0.41	0.02	0.30	0.36	0.79	0.03	1.04	0.38			0.44	0.20		
ZINC00087389	*A. castellanii - L. pneumophila*			0.01	0.02	0.68	0.12	1.05	0.09	1.03	0.05			0.74	0.25		
ZINC19801713	*A. castellanii - L. pneumophila*			0.34	0.18	0.22	0.21	0.73	0.08	0.55	0.08			0.48	0.12		
ZINC00040832	*A. castellanii - L. pneumophila*			0.35	0.31	0.65	0.07	1.07	0.03	1.07	0.27			0.55	0.13		
ZINC03163860	*A. castellanii - L. pneumophila*			0.28	0.10	0.27	0.16	0.79	0.06	0.68	0.13			0.87	0.26		
ZINC04060817	*A. castellanii - L. pneumophila*			0.41	0.11	1.09	0.09	1.04	0.05	1.02	0.15			0.74	0.15		
ZINC05224786	*A. castellanii - L. pneumophila*			0.46	0.08	1.09	0.18	0.96	0.08	1.00	0.19			0.62	0.06		
ZINC05961188	*A. castellanii - L. pneumophila*			0.40	0.12	0.95	0.16	0.83	0.08	1.15	0.05			0.96	0.05		
ZINC02075095	*A. castellanii - L. pneumophila*			0.47	0.10	0.89	0.07	1.00	0.09	0.94	0.15			0.68	0.18		
ZINC00331468	*A. castellanii - L. pneumophila*			0.44	0.10	1.02	0.14	0.99	0.06	1.08	0.22			0.85	0.12		
ZINC00087028	*A. castellanii - L. pneumophila*			0.45	0.07	0.88	0.07	1.05	0.07	1.19	0.32			0.97	0.17		
ZINC01036768	*A. castellanii - L. pneumophila*			0.31	0.05	0.95	0.05	1.03	0.06	0.97	0.41			0.76	0.04		
ZINC01406332	*A. castellanii - L. pneumophila*			0.55	0.06	0.82	0.14	0.98	0.10	1.10	0.09			0.78	0.02		
ZINC00336277	*A. castellanii - L. pneumophila*			0.48	0.12	0.08	0.21	1.01	0.06	1.09	0.06			0.81	0.16		
ZINC00301222	*A. castellanii - L. pneumophila*			0.59	0.12	0.81	0.06	0.99	0.06	1.12	0.21			0.96	0.22		
ZINC02558079	*A. castellanii - L. pneumophila*			0.32	0.05	0.90	0.05	0.83	0.06	1.03	0.08			1.03	0.04		
ZINC03013275	*A. castellanii - L. pneumophila*			0.56	0.19	0.96	0.03	1.04	0.06	0.89	0.06			1.03	0.17		
ZINC04060816	*A. castellanii - L. pneumophila and D. discoideum - M. marinum*			0.50	0.07	0.83	0.05	0.46	0.03	1.03	0.02	0.35	0.08	0.72	0.06	y	y
ZINC04366134	*A. castellanii - L. pneumophila and A. castellanii - M. marinum*	0.58	0.09	0.37	0.13	0.42	0.19	0.16	0.14	0.59	0.01	0.95	0.05	-0.03	0.14		
ZINC03180608	*A. castellanii - L. pneumophila and A. castellanii - M. marinum*	0.61	0.08	0.17	0.12	1.19	0.07	1.18	0.11	1.38	0.05	0.90	0.03	0.12	0.04		
ZINC20446024	*A. castellanii - L. pneumophila and A. castellanii - M. marinum*	0.16	0.17	0.05	0.19	0.57	0.08	0.16	0.07	1.05	0.04	0.72	0.03	0.25	0.07		
ZINC02365627	*A. castellanii - L. pneumophila and A. castellanii - M. marinum*	0.57	0.07	0.48	0.12	0.53	0.10	0.99	0.07	1.18	0.05	0.90	0.09	0.03	0.05		
ZINC00068652	*A. castellanii - L. pneumophila and A. castellanii - M. marinum*	0.56	0.13	0.46	0.08	0.75	0.66	0.78	0.13	0.93	0.05	1.02	0.04	0.35	0.02		
ZINC00347774	*A. castellanii - L. pneumophila and A. castellanii - M. marinum*	0.74	0.05	0.18	0.27	0.34	0.22	0.27	0.17	0.80	0.36	1.27	0.25	0.39	0.05		
ZINC02075448	*A. castellanii - L. pneumophila and A. castellanii - M. marinum*	0.75	0.04	0.43	0.07	0.12	0.08	0.88	0.06	1.05	0.03	0.92	0.04	0.62	0.04		
ZINC00120276	*A. castellanii - M. marinum*	0.72	0.06			0.72	0.15	0.93	0.07	1.50	0.03	0.69	0.06				
ZINC00164027	*A. castellanii - M. marinum*	0.76	0.05			0.48	0.12	0.88	0.04	1.14	0.05	1.14	0.08				
ZINC00439984	*A. castellanii - M. marinum*	0.28	0.03			1.32	0.23	1.52	0.18	0.85	0.03	0.89	0.03				
ZINC01400202	*A. castellanii - M. marinum*	0.54	0.11			0.70	0.04	0.92	0.09	0.94	0.06	1.00	0.04				
ZINC01642343	*A. castellanii - M. marinum*	0.61	0.18			0.86	0.08	1.00	0.07	0.99	0.04	1.13	0.06				
ZINC01718072	*A. castellanii - M. marinum*	0.46	0.10			1.02	0.52	2.15	0.66	0.91	0.06	0.85	0.03				
ZINC05478569	*A. castellanii - M. marinum*	0.44	0.06			0.72	0.03	0.20	0.10	1.49	0.10	0.97	0.07				
ZINC05615643	*A. castellanii - M. marinum*	0.44	0.06			1.17	0.21	0.95	0.04	0.74	0.06	0.71	0.02				
ZINC08743020	*A. castellanii - M. marinum*	0.52	0.06			0.82	0.07	1.09	0.11	0.90	0.09	1.00	0.06				
ZINC16952431	*A. castellanii - M. marinum*	0.28	0.05			0.92	0.09	0.92	0.08	0.93	0.02	0.83	0.06				
ZINC38593154	*A. castellanii - M. marinum*	0.46	0.14			1.06	0.21	0.89	0.08	0.86	0.02	0.83	0.06				
ZINC42311721	*A. castellanii - M. marinum*	0.55	0.08			1.15	0.12	1.06	0.06	0.91	0.05	0.99	0.03				
ZINC43827469	*A. castellanii - M. marinum*	0.48	0.10			0.31	0.10	1.03	0.06	1.31	0.10	1.18	0.06				
ZINC44967378	*A. castellanii - M. marinum*	0.29	0.05			0.72	0.23	0.97	0.05	0.83	0.04	0.81	0.05				
ZINC58759056	*A. castellanii - M. marinum*	0.38	0.07			0.58	0.12	0.78	0.13	0.86	0.10	1.05	0.04				
